# *Vibrio natriegens* as a superior host for the production of c-type cytochromes and difficult-to-express redox proteins

**DOI:** 10.1038/s41598-024-54097-7

**Published:** 2024-03-13

**Authors:** Helena Fuchs, Sophie R. Ullrich, Sabrina Hedrich

**Affiliations:** TU Bergakademie Freiberg, Institute of Biosciences, Leipziger Straße 29, 09599 Freiberg, Germany

**Keywords:** Expression systems, Molecular engineering

## Abstract

C-type cytochromes fulfil many essential roles in both aerobic and anaerobic respiration. Their characterization requires large quantities of protein which can be obtained through heterologous production. Heterologous production of c-type cytochromes in *Escherichia coli* is hindered since the *ccmABCDEFGH* genes necessary for incorporation of heme c are only expressed under anaerobic conditions. Different strategies were devised to bypass this obstacle, such as co-expressing the *ccm* genes from the pEC86 vector. However, co-expression methods restrict the choice of expression host and vector. Here we describe the first use of *Vibrio natriegens* V_max_ X2 for the recombinant production of difficult-to-express redox proteins from the extreme acidophile *Acidithiobacillus ferrooxidans* CCM4253, including three c-type cytochromes. Co-expression of the *ccm* genes was not required to produce holo-c-type cytochromes in V_max_ X2. *E. coli* T7 Express only produced holo-c-type cytochromes during co-expression of the *ccm* genes and was not able to produce the inner membrane cytochrome CycA. Additionally, V_max_ X2 cell extracts contained higher portions of recombinant holo-proteins than T7 Express cell extracts. All redox proteins were translocated to the intended cell compartment in both hosts. In conclusion, *V. natriegens* represents a promising alternative for the production of c-type cytochromes and difficult-to-express redox proteins.

## Introduction

Cytochromes play essential roles as electron carriers during both aerobic and anaerobic respiration in all organisms. They all contain heme as a co-factor. The heme c co-factor in c-type cytochromes (Cytc) is covalently bound to the protein. The consensus motif, CXXCH, of Cytc contains two highly conserved cysteine residues which form thioether bonds with the heme while the histidine acts as an axial ligand to the iron^1^.

**Figure 1:**
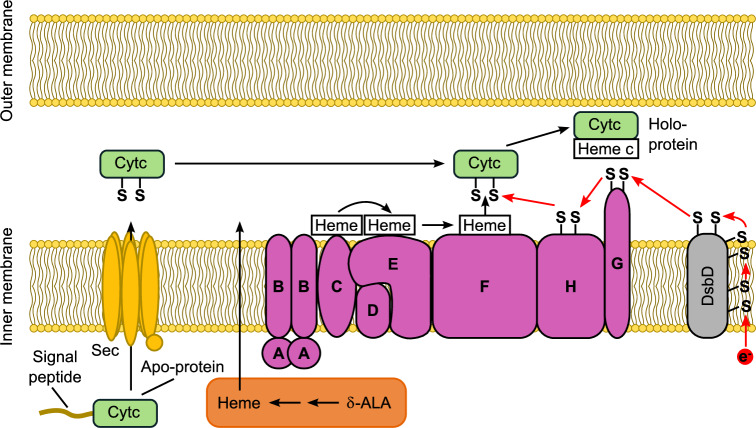
Post-translational modifications required for the production of holo-c-type cytochromes (Cytc) in *E. coli*^3–5^. Transcription and translation of the Cytc encoding gene and mRNA take place in the cytoplasm while co-factor integration and folding occur in the periplasm. The signal peptide of the apo-protein leads to translocation into the periplasm via the general secretory (Sec) translocation pathway^29^. Heme is synthesized in the cytoplasm from δ-aminolevulinic acid (δ-ALA), transported into the periplasm and delivered to the apo-Cytc via CcmC, CcmE, and CcmF. CcmF catalyzes the covalent attachment of heme to the cysteine residues of the Cytc binding motif. CcmA and CcmB are predicted to be transporter proteins. However, it is unclear what substrate they transport. CcmD stabilizes CcmE in the membrane. Electrons required for the formation of the thioether bonds are delivered to the apo-Cytc via CcmH, CcmG, and DsbD. After integration of heme c and folding, holo-Cytc remains soluble in the periplasm or is integrated into a membrane.

The ability to produce large quantities of protein during heterologous expression is essential for the characterization and study of novel as well as known proteins. C-type cytochromes pose one of the greatest challenges for heterologous production due to the extensive post-translational modifications required to produce holo-Cytc (Fig. 1). While gene transcription and translation take place in the cytoplasm, c-type cytochromes are known to function as soluble periplasmic proteins (or soluble proteins in the intermembrane space of mitochondria) or membrane-anchored proteins^2^. Therefore, the apo-protein needs to be translocated across the inner membrane into the periplasm. Heme synthesis also has a resource demand on host cells, especially during over-expression. After heme is transported into the periplasmic space, a set of eight proteins is required to deliver the heme to the apo-Cytc and facilitate covalent attachment in *Escherichia coli*. These proteins are termed CcmABCDEFGH for cytochrome c maturation and are located on the periplasmic site of the inner membrane^3–5^. *E. coli* expression strains, such as BL21(DE3) and T7 Express, possess chromosomal copies of the corresponding genes. They are part of the *aeg46.5* operon which is not active under aerobic conditions^6^. As a consequence, the production of holo-Cytc is not possible in aerobically grown *E. coli* cells. To circumvent this, a strategy for the co-expression of the *ccmA-H* genes from an additional plasmid, termed pEC86, was developed. This enables the production of holo-Cytc under aerobic conditions^2,7^. Additionally, heme pre-cursors, such as δ-aminolevulinic acid (δ-ALA), can be supplemented to overcome bottlenecks in heme biosynthesis^8^. Heme can also be directly supplemented to the culture medium when heme uptake systems are co-expressed in *E. coli*^9,10^. However, these co-expression methods restrict the choice of expression host, vector, selection marker, and can also negatively impact growth and yield.

Here, we describe a different expression host, the marine bacterium *Vibrio natriegens*, for the production of difficult-to-express redox proteins, including three c-type cytochromes from the extreme acidophile *Acidithiobacillus ferrooxidans* CCM4253. *V. natriegens* (initially referred to as *Pseudomonas natriegens*) is an emerging host for heterologous protein production with a remarkable doubling time of under ten minutes^11^, making it the fastest growing bacterium known to date. It’s greater biomass synthesis rate and stronger protein expression ability^12^ are partially caused by the 20–40% higher ribosome number per cell compared to *E. coli*^13^. *V. natriegens* grows well on various industrially relevant substrates and exhibits a higher feedstock flexibility compared to *E. coli*^14,15^. Additionally, there is already a wide array of genetic tools available for *V. natriegens,* such as expression vectors with various backbones, promoters, and tags^16–18^. Protocols for DNA transformation based on electroporation and heat shock^16,19^, methods to manipulate gene expression via CRISPR interference (CRISPRi)^20^, as well as genome editing techniques based on multiplex genome editing by natural transformation (MuGENT)^21^ and natural transformation CRISPR (NT-CRISPR)^22^ have also been established*.* The strain V_max_ X2 was engineered from the type strain ATCC 14048 by inserting a T7 RNA polymerase cassette under the control of a *lacUV5* promotor in the *dns* locus^16^. *V. natriegens* has been shown to produce soluble periplasmic^17^ as well as the functional multi-subunit membrane protein complex NADH:quinone oxidoreducase (NQR) and the secondary transport system Mrp from *Vibrio cholerae*^23^. Eichmann et al.^17^ showed that production of the difficult-to-express proteins lucimycin and uricase was higher in *V. natriegens* than *E. coli* BL21(DE3) and Becker et al.^24^ demonstrated higher production levels for isotopically labelled FK506 binding protein (FKBP) as well as enhanced yellow fluorescent protein (EYFP) in *V. natriegens* compared to *E. coli* BL21(DE3). Xu et al.^25^ also demonstrated that *V. natriegens* has a complementary expression spectrum to *E. coli*. Proteins that *E. coli* failed to produce in a soluble state were produced as soluble proteins in *V. natriegens* with up to 12 times higher expression. In addition, *V. natriegens* seems to have an extremely broad over-expression spectrum encompassing more than six enzyme families from bacterial, fungal, and plant origin. *V. natriegens* also produced higher amounts of an archaeal catalase-peroxidase (AfKatG) compared to *E. coli* BL21(DE3)^26^. A particular advantage is the production of smaller soluble proteins, which might be helpful in avoiding bottlenecks (i.e. translocation and folding) during the production of soluble periplasmic proteins with *E. coli*^25^.

In this study, we compared the production of three c-type cytochromes, the inner membrane-anchored CycA, the soluble periplasmic Cytc Cyc1, and the outer-membrane Cytc Cyc2, as well as the soluble blue copper protein rusticyanin (Rus) in *V. natriegens* V_max_ X2 and *E. coli* T7 Express. V_max_ X2 proved to be a promising expression host for the production of difficult-to-express Cytc. In contrast to T7 Express, V_max_ X2 did not require co-expression of the *ccm* genes from a second plasmid to produce holo-Cytc.

## Results and discussion

Genes encoding the c-type cytochromes CycA, Cyc1, and Cyc2 were co-expressed with the Rus encoding gene from *At. ferrooxidans* CCM4253 in order to compare the performance of V_max_ X2 and T7 Express regarding their ability to produce recombinant redox proteins. All gene sequences were codon optimized for *E. coli* K-12. Gene and protein sequences are listed in Supplementary Note 1. The strains and plasmids used in this study are listed in Supplementary Table 1. All proteins are part of the electron transfer chain of *At. ferrooxidans* for the reduction of ferric iron under anaerobic conditions^27^. CycA is anchored to the inner membrane on the periplasmic site, while Cyc1 and Rus are soluble periplasmic proteins. Cyc2 is a typical β-barrel protein^28^ that is fully integrated into the outer membrane. N-terminal signal peptides for translocation across the inner membrane via the general secretory (Sec) pathway^29^ were predicted for all proteins. Gene sequences used for the heterologous expression contained the unmodified native signal peptide sequences.

### *V. natriegens* produces higher amounts of holo-rusticyanin

**Figure 2:**
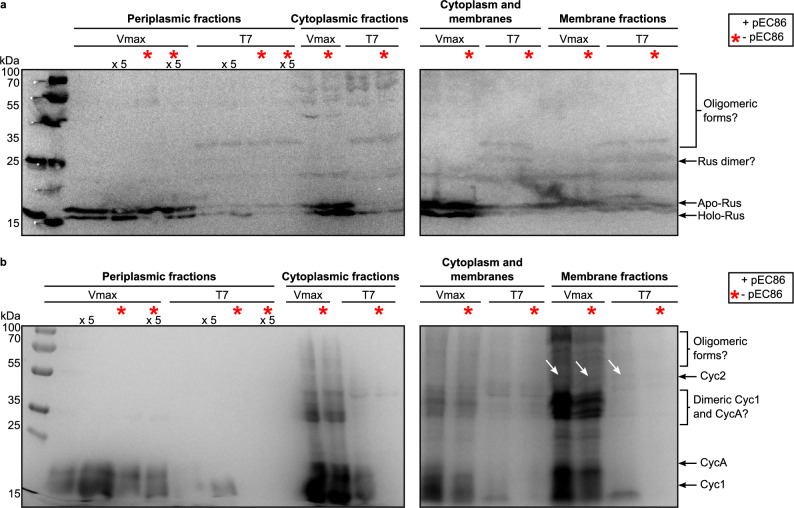
Immunodetection of rusticyanin (Rus) and 3,3ʹ,5,5ʹ-tetramethylbenzidine (TMBZ) stain for the detection of holo-c-type cytochromes. Fresh cells were fractionated according to Petiti et al.^50^. Protein concentrations of each fraction were determined with a BCA assay in triplicates. For unconcentrated fractions, 150 μg total protein was loaded onto SDS-PAGE gels, 300 μg total protein was loaded for the concentrated periplasmic fractions (x 5). All samples, gels, Western blots, and immunodetections were processed in parallel. Samples from cells without the pEC86 plasmid are marked with a red star. (**a**) Immunodetection of Rus in protein extracts from different cell compartments. A holo-Rus positive control (0.33 μg), isolated from *At. ferrooxidans*, was loaded left to the molecular weight marker. Holo-Rus possesses a molecular weight of 16.6 kDa and apo-Rus 19.9 kDa. (**b**) TMBZ-stain^34^ of holo-c-type cytochromes in protein extracts from different cell compartments. The cytochromes possess the following molecular weights: (1) holo-Cyc1 20.0 kDa, (2) holo-CycA 22.3 kDa, (3) holo-Cyc2 49.3 kDa. The bands corresponding to Cyc2 are marked with white arrows.

*Vibrio natriegens* V_max_ X2 and *E. coli* T7 Express containing either both the pEC86 and pET16bP_cycA_cyc1_rus_cyc2 vectors or only the pET16bP_cycA_cyc1_rus_cyc2 vector (Supplementary Table 1) were used to heterologously produce rusticyanin from *At. ferrooxidans* CCM4253. The periplasm, cytoplasm, and membranes were separated to determine correct translocation. Rus was detected via immunodetection with specific antibodies after separation by SDS-PAGE and subsequent western blotting. Apo-Rus with the attached signal peptide has a size of 19.9 kDa, while holo-Rus, from which the signal peptide was cleaved, was detected at the expected weight (16.6 kDa) (Fig. 2a).

While both strains were able to produce holo-Rus, it seems to comprise a greater relative portion of the total protein in V_max_ X2 cell extracts. The natural signal peptide was recognized and cleaved in both strains, evident from the holo-Rus detected in their periplasm. Apart from the periplasm, holo-Rus was also detected in all other cell compartments. Since the signal peptide is cleaved during translocation into the periplasm, holo-Rus should not be present in the cytoplasm. However, Rus tends to associate itself with the membranes of the neutrophilic hosts and can be resolubilized by washing with an acidic buffer (Supplementary Fig. 1). Therefore, membrane association likely occurs via weak electrostatic interactions due to a different surface charge at neutral pH. This corresponds to the very high predicted pI of 8.0 for holo-Rus (Supplementary Note 2). Bengrine et al.^30^ also described weak attachment of Rus to the cell membranes of *E. coli* expression hosts combined with a lack of soluble Rus in the periplasmic fraction. Native Rus was also associated to the membranes of *At. ferrooxidans* in different studies^31,32^ rather than remaining soluble in the periplasm when cells were lysed by sonication and French press at pH 7 instead of pH 2. This corroborates our assumption that membrane association is caused by a different surface charge at neutral pH. Fully matured periplasmic proteins could have also been detected in the cytoplasmic fraction because some cells remained intact during the osmotic shock. This is especially relevant for V_max_ X2 since Rus seems to comprise a greater relative portion of the total protein in the samples. In order to minimize the portion of intact cells, dissociation of the outer membrane was monitored microscopically by observing cell morphology and movement. Membrane-associated Rus also may have been detached from the membranes and solubilized during sonication of the spheroplasts with some remaining associated to the membranes.

Rus from *At. ferrooxidans* seems to occur as a monomer and dimer, judging from the size of the higher molecular weight band. Rus is predicted to function as a monomer and may form a respiratory super-complex with Cyc1 and Cyc2^28,33^. Therefore, it is unclear whether the dimer also fulfils a role in electron transfer or if it is just a result of sample preparation before SDS-PAGE. Oligomeric forms were also detected in the neutrophilic hosts V_max_ X2 and T7 Express, with T7 Express forming more oligomers, especially supposed dimers of the apo- and holo-protein.

### Holo-c-type cytochromes are produced in *V. natriegens* without co-expression of the *ccm* genes

The periplasm, cytoplasm, and membranes were separated to determine correct translocation. Holo-Cytc was detected via TMBZ-staining^34^ after separation by SDS-PAGE. Since SDS-PAGE was performed under denaturing conditions, only covalently bound hemes, i.e. heme c, were detected. Holo-Cyc1 had a size of 20.0 kDa, Holo-CycA possessed a molecular weight of 22.3 kDa, and Holo-Cyc2 had a size of 49.3 kDa (Fig. 2b).

**Figure 3:**
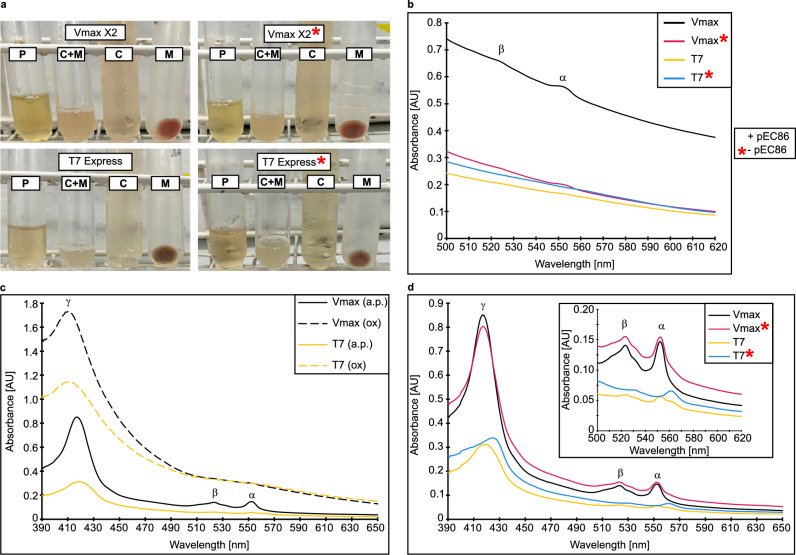
UV/Vis spectra of protein extracts from different cell compartments of V_max_ X2 and T7 Express. Fresh cells were fractionated according to Petiti et al.^50^. A 100 μL sample was used for each measurement. Measurements were performed in TES buffer diluted 1:2 in water at pH 8.0. The periplasmic fractions were concentrated five times in centricons with a molecular weight cut-off of 10 kDa. Strains without the pEC86 plasmid are labelled with a red star. V_max_ X2 carrying the pEC86 plasmid is coloured in black, V_max_ X2 without pEC86 in red, T7 Express with pEC86 in yellow, and T7 Express without pEC86 in blue. Spectra of reduced samples are displayed with solid lines and spectra of oxidized samples with dashed lines. The three characteristic absorbance maxima of reduced heme c are labelled α (550 nm), β (525 nm), and γ (410–420 nm) respectively^1,2,35^. Oxidation was achieved by the addition of up to 10 mM Na_2_[IrCl_6_], and reduction by adding Na-dithionite. (**a**) Pictures of cell compartment fractions after preparation. Legend: P—periplasmic fraction; C + M—cytoplasm and membranes; C—cytoplasmic fraction; M—membrane fraction. (**b**) Comparison of the α- and β-peaks of heme in the periplasmic fractions. (**c**) Comparison of spectra of reduced (a.p.) and oxidized (ox) heme c in the cytoplasm of V_max_ X2 and T7 Express with pEC86. (**d**) Comparison of UV/Vis spectra of the cytoplasmic fractions of V_max_ X2 and T7 Express with and without pEC86. The absorbance maxima in the cytoplasmic fraction of T7 Express without pEC86 are shifted to slightly longer wavelengths, indicating the presence of heme b instead of heme c^1,2,35^.

V_max_ X2 produced all holo-c-type cytochromes under all conditions, while holo-CycA was not detected in T7 Express. Additionally, T7 Express was not able to produce any of the tested Holo-Cytc when the *ccmA-H* genes were not co-expressed (marked with red star in Fig. 2). The soluble periplasmic Cytc, Cyc1, was also detected in other compartments besides the periplasm, similar to Rus. Since an even higher pI of 8.86 was predicted for holo-Cyc1 (Supplementary Note 2), the protein was likely also weakly associated to the membranes and was washed off the membranes with an acidic buffer (Supplementary Fig. 1). The other two membrane-anchored or bound cytochromes were only detected in the membrane fractions. The band intensity of Holo-Cyc2 (marked with white arrows in Fig. 2b) after heme-staining is very low since it harbours only one heme c binding site^28^ while CycA and Cyc1 are diheme cytochromes. As with Rus, oligomeric forms of the cytochromes were also detected in the TMBZ-stained extracts of V_max_ X2 and T7 Express, with the most prominent bands presumably representing dimeric forms of Cyc1 and CycA just above the 35 kDa marker band (Fig. 2b). High-molecular weight aggregates of the cytochromes were also detected. The band intensities in Fig. 2b suggest a greater relative portion of holo-Cytc in V_max_ X2 cell extracts, which is supported by UV/Vis spectra recorded from the soluble fractions (Fig. 3).

Cell extracts and membrane pellets obtained from V_max_ X2 during fractionation appeared reddish, indicating the presence of holo-Cytc (Fig. 3a). UV/Vis spectra of Na-dithionite treated fractions from V_max_ X2 (Fig. 3b–d) revealed higher absorbance intensities, corresponding to a higher Cytc content. The absorbance maxima of the α- and β-peak of T7 Express without the pEC86 plasmid are different than those of the other extracts (Fig. 3d). Typically, heme c exhibits absorbance maxima at 410 nm (γ-peak), 525 nm (β-peak), and 550 nm (α-peak) in its reduced state. These maxima are shifted to slightly longer wavelengths in heme b^1,2,35^. Such heme b specific peaks were detected in fractions of T7 Express without the pEC86 vector, suggesting that only host-derived b-type cytochromes were enriched. This can be attributed to the heme b co-factor of the cytochrome *bo*_*3*_^36^ and cytochrome *bd-I*^37,38^ ubiquinol oxidases of *E. coli*, demonstrating that T7 Express produced no detectable amount of heme c under aerobic conditions, as described previously^2,7^. Absorbance maxima corresponding to heme c were only detected when the *ccm* genes were co-expressed. However, expression levels in T7 Express were very low. In contrast, heme c was also detected in extracts from aerobically grown V_max_ X2 cells which did not carry the pEC86 plasmid. Peak shoulders corresponding to heme b were also detected in V_max_ X2 protein extracts. This strain most likely also natively produces b-type cytochromes since genes predicted to encode subunits of a cytochrome *bd-I* (*cydABX*) and cytochrome *bo*_*3*_ (*cyoA-D*) oxidase are located on chromosome 1 and 2 of *V. natriegens* ATCC 14048 respectively.

The difference in expression levels of holo-Cytc in V_max_ X2 cells carrying the pEC86 plasmid to cells without the pEC86 plasmid seems to be negligible, while the presence of the pEC86 vector in T7 Express cells only leads to low expression levels of holo-Cytc. This makes *V. natriegens* an interesting production host for recombinant c-type cytochromes.

The spectra in Fig. 3c demonstrate that recombinant Cytc produced by V_max_ X2 and T7 Express was redox-active. Upon sample preparation, heme c was completely reduced and could be oxidized by adding up to 10 mM Na_2_[IrCl_6_]. The α- and β-peaks disappeared upon oxidation and were replaced by a broad plateau. Additionally, the γ-peak shifted to slightly shorter wavelengths^1,2,35^. Oxidized heme c was re-reduced upon addition of sodium dithionite (Supplementary Fig. 2). This redox activity suggests that heme c was integrated in the heterologous c-type cytochromes despite the difference in extracellular and periplasmic pH. Considering that holo-CycA was not detected in *E. coli* protein extracts (Fig. 2b), *V. natriegens* seems to be a more suitable host for the production of difficult-to-express redox proteins from extreme acidophiles.

Additionally, *V. natriegens* V_max_ X2 exhibited faster growth than *E. coli* T7 Express, especially in the early exponential growth phase (Supplementary Fig. 3), resulting in a time saving of several hours during experiments. Fast growth of *V. natriegens* was observed by us in traditional *E. coli* media (e.g. LB, 2xYT, e2xYT) at temperatures of 20–37 °C, while the fastest growth was achieved by supplementing 15 g L^-1^ NaCl^14^ or a mixture of NaCl, KCl, and MgCl_2_^16^ at 30 °C and 37 °C. *V. natriegens* also produced 35% more biomass (cell wet weight, Supplementary Table 4) than *E. coli* under the same starting conditions. The higher band intensities of target proteins in samples of *V. natriegens* suggest a higher proportion of target protein per μg loaded protein in comparison to *E. coli*.

### A different *ccm* operon structure enables *V. natriegens* to produce holo-c-type cytochromes under aerobic conditions

**Figure 4:**
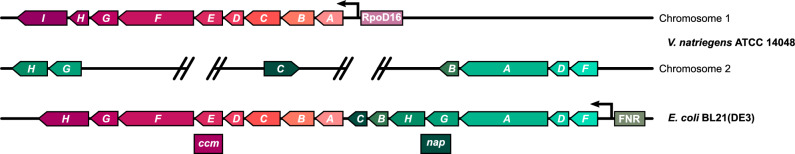
Comparison of the chromosomal *ccm* operon organization in *V. natriegens* and *E. coli*. The *ccm* genes (cytochrome c maturation; red) in *V. natriegens* form a separate operon on chromosome 1, while the *nap* genes (green), encoding a periplasmic nitrate reductase^42^, are distributed across chromosome 2. Chromosomal promoter prediction revealed a potential promoter region upstream of *ccmA*. A RpoD16 binding site was predicted in this region, suggesting that the *ccm* genes are expressed during exponential aerobic growth. In *E. coli* the *ccm* genes are part of the *aeg46.5* operon together with the *nap* genes. Expression of all genes in this operon is only activated under anaerobic conditions^6^. A fumarate nitrate regulator (FNR)^43^ binding site was predicted in the promoter region upstream of *napF*.

Both *V. natriegens* and *E. coli* possess chromosomal copies of the *ccm* genes. The *E. coli* Cytc maturation has been intensely studied, although exact functions of some of the eight proteins are still not entirely clear^3–5^. While CcmA and CcmB are predicted to be transporter proteins it is unclear what substrate they transport. CcmC, CcmE, as well as CcmF deliver heme to the apo-Cytc while CcmD seems to stabilize CcmE in the membrane. Electrons for the thioether bond formation between the cysteines and heme are delivered from a cytoplasmic thioreductase to DsbD, which transfers the electrons to CcmG and CcmH (Fig. 1). In *V. natriegens,* CcmH is a much smaller protein while the CcmI encoding gene presents an additional gene in the *ccm* operon. The CcmH from *E. coli* seems to be a fusion protein consisting of two domains. The N-terminal domain is homologous to Ccl2/CycL, a heme biogenesis protein present in *Rhodobacter capsulatus* and *Bradyrhizobium japonicum*^39^. The C-terminal domain is homologous to CcmI/CycH from other microorganisms, such as *R. capsulatus*^39^ and *V. cholerae*^40^. Interestingly, the C-terminal CcmH domain of *E. coli* lacks the N-terminal portion of CcmI/CycH which is required for the biogenesis of c_1_ cytochromes^39^ since *E. coli* does not possess a cytochrome *bc*_*1*_ complex. The C-terminal domain seems also not essential for Cytc maturation in *E. coli*^41^. The chromosomal *ccm* operon structures of *V. natriegens* ATCC 14048 (original strain from which V_max_ X2 was derived) and *E. coli* BL21(DE3) (T7 Express is a BL21 derivative) are depicted in Fig. 4.

In *E. coli*, the *ccm* genes are part of the *aeg46.5* operon together with the *nap* genes, encoding a periplasmic nitrate reductase^42^. Expression of all genes in this operon is only activated under anaerobic conditions^6^. A fumarate nitrate regulator (FNR)^43^ binding site was predicted in the promoter region upstream of *napF* (Supplementary Note 3). FNR is a global positive regulator for the expression of genes required for anaerobic metabolism in *E. coli*^43,44^. The protein contains an oxygen-sensitive [4Fe-4S]^2+^ cluster in its functional state under anaerobic conditions, increasing dimerization and site-specific DNA-binding. Under oxygen-limited conditions and in the presence of nitrate as a terminal electron acceptor, transcription of the *aeg46.5* operon in *E. coli* is activated. In addition to the *napABCDFGH* genes, the *ccmABCDEFGH* genes are also encoded on this operon since the *E. coli* nitrate reductase complex contains heme c which must be synthesized accordingly^6^. Contrarily, the *ccm* genes in *V. natriegens* form a separate operon on chromosome 1, while the *nap* genes are distributed across chromosome 2. Chromosomal promoter prediction revealed a potential promoter region upstream of *ccmA*. A RpoD16 binding site was predicted in this region (Supplementary Note 3). The *rpoD* gene product is the σ^70^ factor which is responsible for the transcription of genes during exponential aerobic growth^45^.

## Conclusion

Our study demonstrates that *V. natriegens* is a promising host for the production of c-type cytochromes for several reasons: (1) it can produce holo-c-type cytochromes without co-expressing additional genes from a second plasmid, (2) the supplementation of heme or its pre-cursors is not necessary to obtain holo-c-type cytochromes, (3) the proportion of target protein per μg loaded protein in *V. natriegens* was higher than in *E. coli*, (4) it exhibits faster growth and higher biomass production than *E. coli*. Our results demonstrate that expression vectors and strategies as well as codon-optimized gene sequences for *E. coli* can be easily transferred to *V. natriegens*. We were able to show that a switch can be beneficial for the heterologous production of proteins with complex co-factors, such as heme c, that *E. coli* struggles to integrate and especially for the production of membrane-anchored proteins. *V. natriegens* was capable of producing an electron transfer chain from an extremely acidophilic bacterium spanning both membranes and the periplasm. In future studies we hope to demonstrate the in vivo functionality of this electron transfer chain in *V. natriegens*.

## Methods

### Strains and plasmids

*E. coli* NEB5α (New England BioLabs) was used for plasmid propagation and purification. *E. coli* T7 Express (New England BioLabs) and *V. natriegens* V_max_ X2 (TelesisBio) were used for heterologous gene expression. Coding sequences of the four redox proteins CycA2, Cyc1A, Rus, and Cyc2A were obtained from GenBank (accession number QKQP01000005.1; locus-tags DN052_09955, DN052_11970, DN052_11940, and DN052_11975). All gene sequences were codon optimized for *E. coli* K-12 with JCat^46^ (http://www.jcat.de/Start.jsp). Signal peptides were predicted with SignalP6.0^47^ (https://services.healthtech.dtu.dk/services/SignalP-6.0/). Protein properties for apo- and holo-proteins alike were predicted with ProtParam^48^ (https://web.expasy.org/protparam/). Original and optimized gene and protein sequences are listed in Supplementary Note 1. Prediction results are available in Supplementary Note 2. Optimized gene sequences were synthesized, cloned into a pUC57 plasmid, and sequenced by GeneCust (Boynes, France). Vector maps are available in Supplementary Figs. 4–7. Plasmid sequences are available in Supplementary Data 1. The pEC86 vector encoding the *ccmABCDEFGH* genes from *E. coli* was obtained from the Culture Collection of Switzerland (CCOS891). The pET16bP vector was obtained from U. Wehemeyer (unpublished). The plasmid sequence is available in Supplementary Data 1.

The pET16bP_cycA_cyc1_rus_cyc2 expression vector was obtained by cloning restriction digestion fragments of all genes into the *Nco*I/*Not*I restriction site of the pET16bP vector. The ribosome binding site (RBS) and linker of the pET16b vector was cloned upstream of each gene. Successful cloning of all genes was confirmed by sequencing (Eurofins). A vector map is available in Supplementary Fig. 8. The plasmid sequence is available in Supplementary Data 1. The pEC86 and pET16bP_cycA_cyc1_rus_cyc2 vectors were successively transformed into *E. coli* T7 Express and *V. natriegens* V_max_ X2 via heat shock transformation, according to the manufacturer’s instructions. Chemically competent cells containing the pEC86 vector were prepared as described below. All strains, primers, and plasmids used in this study are listed in Supplementary Table 1. Antibiotic concentrations used for selection and media recipes are listed in Supplementary Tables 2 and 3.

### Preparation of chemically competent cells

After heat shock transformation of the pEC86 vector, *E. coli* T7 Express and *V. natriegens* V_max_ X2 were cultivated on Luria–Bertani (LB) plates (+ v2 salts) with appropriate antibiotics at 37 °C overnight. Pre-cultures (20 mL, LB [+ v2 salts]) containing appropriate antibiotics were inoculated from a single colony and incubated at 37 °C (30 °C for V_max_ X2) and 120 rpm overnight. 50 mL of LB (+ v2 salts) medium in a 250 mL baffled flask with appropriate antibiotics was inoculated to an OD_600_ ≈ 0.05 and incubated at 37 °C (30 °C for V_max_ X2) and 120 rpm. Cells were harvested at 4 °C, 5000 *xg* for 10 min when the OD_600_ reached 0.5. Cells were subsequently handled on ice and all buffers and reaction tubes were pre-cooled on ice. Cells from a 50 mL culture were carefully resuspended in 16 mL CCMB80 buffer (10 mM potassium acetate, 80 mM CaCl_2_⋅2H_2_O, 20 mM MnCl_2_⋅2H_2_O, 10 mM MgCl_2_⋅6H_2_O, 25% [v/v] glycerol, pH 6.4) and left on ice for 20 min. Cells were centrifuged (4 °C, 5000 x*g*, 10 min) and gently resuspended in 16 mL (or 650 μL for V_max_ X2) CCMB80 buffer. Chemically competent cells were either used directly for heat shock transformation according to the manufacturer’s instructions or frozen in liquid N_2_ and stored at − 80 °C.

### Heterologous protein production

Cells from a cryo-stock were streaked on a LB (+ v2 salts) agar plate containing appropriate antibiotics. Plates were incubated at 37 °C (30 °C for V_max_ X2) overnight or at room temperature over two days. Pre-cultures were inoculated from a single colony and incubated at 37 °C (30 °C for V_max_) and 120 rpm overnight. Enhanced 2xYT medium (e2xYT), supplemented with v2 salts when necessary, was used for pre-cultures. For V_max_ X2, e2xYT based ZYM-5052 + v2 salts autoinduction medium according to Studier^49^ was used for expression, while *E. coli* T7 Express was cultivated in e2xYT medium. Expressions were carried out in 250 mL baffled flasks containing 50 mL medium and appropriate antibiotics. Main cultures were inoculated to an OD_600_ of 0.05 and incubated at 30 °C and 120 rpm. When the OD_600_ reached 0.6, 10 μM IPTG was added to *E. coli* cultures and all cultures were transferred to 25 °C and shaken at 120 rpm overnight. Since highly concentrated complex media were used for expression, heme or its pre-cursors were not supplied to the medium. Growth curves are available in Supplementary Fig. 3.

### Cell fractionation

Fractionation of periplasmic, cytoplasmic, and membrane proteins was performed following the protocol of Petiti et al.^50^. All steps were performed at 4 °C or on ice, all buffers were pre-cooled on ice. Cells from a 50 mL culture were harvested at 5000 x*g* for 5 min. The cell wet weight is available in Supplementary Table 4. Pellets were carefully resuspended in 500 μL TES buffer (200 mM Tris–HCl pH 8.0, 0.5 mM EDTA, 0.5 M sucrose). 120 U lysozyme (in TES buffer) and 1.8 mL TES diluted 1:2 in water were added. Cells were shaken horizontally on ice for 30 min and centrifuged at 5000 x*g* for 5 min (8 min for V_max_ X2). The supernatant was kept as the periplasmic fraction (P). Spheroplasts were resuspended in 10 mL TES diluted 1:2 in water. One Pierce Protease Inhibitor Tablet (Thermo Scientific), 2 mM MgCl_2_⋅6H_2_O, and 40 U DNaseI were added. Spheroplasts were disrupted by sonication at 70% intensity twice for 30 s and centrifuged at 2000 x*g* for 5 min (8 min for V_max_). The supernatant was labelled as the cytoplasmic and membrane fraction (C + M) and centrifuged at 40,000 x*g* for 2 h to pellet membranes. The membranes (M) were resuspended in 2.5 mL TES diluted 1:2 in water overnight and the supernatant was kept as the cytoplasmic fraction (C). The total protein concentration of each fraction was determined in a 96-well plate with the Pierce BCA protein assay kit (Thermo Scientific) using triplicates according to the manufacturer’s instructions. Calibration was performed according to the manufacturer’s instructions with bovine serum albumin (BSA) in 1:2 diluted TES buffer in a 96-well plate using triplicates. TES buffer diluted 1:2 with water was used as blank for all measurements. The BCA assay results available in Supplementary Table 5. Samples for SDS-PAGE analysis were prepared for each fraction by mixing 200 μL sample (diluted with TES when necessary) with 100 μL 6× Laemmli buffer (375 mM Tris–HCl [pH 6.4], 9% [w/v] SDS, 0.03% [w/v] bromophenol blue, 50% [v/v] glycerol) and denatured at 95 °C for 5 min. The periplasmic fractions were concentrated 5× in a VivaSpin 500 centricon (molecular weight cut-off 10,000 Da) and prepared for SDS-PAGE analysis accordingly.

### SDS-PAGE and Western blotting

SDS-PAGE was performed with Novex WedgeWell 10–20% Tris–Glycine gels (Thermo Scientific). Frozen denatured samples were thawed at 40 °C and centrifuged for 2 min at full speed. 150 μg total protein was loaded onto the gel. For concentrated periplasmic fractions 300 μg total protein was used. PageRuler Plus Prestained Protein Ladder (Thermo Scientific) was used as size marker. When necessary, 0.33 μg of purified rusticyanin from *At. ferrooxidans*, kindly provided by Marianne Ilbert (Bioenergetic and Protein Engineering Laboratory, BIP, Institute of Microbiology of the Mediterranean, IMM), was used as positive control for immunodetection. Separation was performed in a Tris–glycine running buffer (25 mM Tris, 192 mM glycine, 0.1% [w/v] SDS, pH 8.3) at 125 V. Western blotting of gels was performed with the Mini Gel Tank and Blot Module Set (Thermo Scientific). Proteins were transferred to a PVDF membrane (pore size 0.2 μm) for 1 h at 20 V in a Tris–glycine transfer buffer (12 mM Tris–HCl [pH 8.3], 96 mM glycine).

#### TMBZ-staining of SDS-PAGE gels

TMBZ-staining was performed according to Thomas et al.^34^. This staining method relies on the peroxidase activity of heme. SDS-PAGE gels containing heme proteins are incubated in a TMBZ solution. Oxidation of TMBZ is catalysed by the peroxidase activity of the heme group upon addition of H_2_O_2_, leading to a blue precipitate. SDS-PAGE gels were incubated in seven parts 250 mM sodium acetate pH 5.0 and three parts 6.3 mM TMBZ in methanol for 2 h in the dark without shaking. 50–150 μL 30% hydrogen peroxide was added for colorimetric detection of covalently bound heme c. Gels were incubated in seven parts 250 mM sodium acetate pH 5.0 and three parts 2-propanol immediately after staining and kept in this solution in the dark without shaking overnight. Gels were visualized with a ChemiDoc XRS + gel imaging system (Bio-Rad) running ImageLab software (Bio-Rad). Unprocessed gel images are available in Supplementary Figs. 10 and 12.

#### Immunodetection

Membranes were blocked by incubation in TBS-T (19.3 mM Tris, 150 mM NaCl, 0.1% [v/v] Tween 20, pH 7.6) with 5% (w/v) non-fat dried milk powder for 60 min under gentle shaking. The primary rabbit anti-Rus antibody was diluted 1:1000 in TBS-T containing 5% (w/v) non-fat dried milk powder. Rabbit anti-Rus antibodies were kindly provided by Marianne Ilbert (Bioenergetic and Protein Engineering Laboratory, BIP, Institute of Microbiology of the Mediterranean, IMM). Membranes were incubated with primary antibodies for 1 h under gentle shaking. Afterwards, membranes were washed for a total of 30 min with TBS-T and incubated with Pierce goat anti-rabbit IgG-HRP secondary antibodies (Thermo Scientific) for 1 h under gentle shaking. Secondary antibodies were diluted 1:5000 with TBS-T containing 5% (w/v) non-fat dried milk powder. After a second washing step with TBS-T, 1-Step Ultra TMB-Blotting Solution (Thermo Scientific) was added for colorimetric detection. Membranes were visualized with a ChemiDoc XRS + gel imaging system (Bio-Rad) running ImageLab software (Bio-Rad). Unprocessed images are available in Supplementary Figs. 9 and 11.

### UV/Vis spectroscopy

UV/Vis spectroscopy was performed with a Specord 50 Plus spectrometer (Analytik Jena) running WinAspect Plus (Analytik Jena). Absorbance was measured from 390 to 750 nm in increments of 0.5 nm. Measurements were carried out in TES buffer diluted 1:2 in water (as prepared). Samples were incubated for 30 min on ice with up to 10 mM Na_2_[IrCl_6_] or a spatula tip of Na-dithionite for oxidation or reduction respectively. Buffer absorbance was used as a reference. All recorded UV/Vis spectra are available in Supplementary Figs. 13 and 14.

### Prediction of chromosomal promoters

Bacterial promoters were predicted with BPROM^51^ (http://www.softberry.com/berry.phtml?topic=bprom&group=programs&subgroup=gfindb) using only the operon sequence with its upstream region as input. Genomic sequences for *V. natriegens* ATCC 14048 (accession numbers NZ_CP016345 and NZ_CP016346) and *E. coli* BL21(DE3) (accession number NZ_CP053602) were retrieved from GenBank. Input sequences and results are listed in Supplementary Note 3.

### Supplementary Information

Supplementary Information.

## Data Availability

Accession numbers and locus-tags for existing gene and genome sequences retrieved from GenBank are listed in the appropriate Methods sections and in Supplementary Notes 1 and Note 3. The plasmid sequences and unprocessed gel images are available in Supplementary Data 1 and Supplementary Figs. 9–12 respectively. BCA assay results and UV/Vis spectra are available in Supplementary Table 5 and Supplementary Figs. 13 and 14. Sequencing results and raw data (BCA assay, UV/Vis spectra) that support the findings of this study are available from the corresponding author upon reasonable request. The authors declare that all other data supporting the findings of this study are available within the paper and its supplementary information files.
