# Supplementary material for: Vibrio natriegens as a superior host for the production of c-type cytochromes and difficult-to-express redox proteins
Source: Sci Rep. 2024 Mar 13;14:6093. doi: 10.1038/s41598-024-54097-7 (PMC10937671; doi:10.1038/s41598-024-54097-7)
Supplement: Supplementary file 1 — Supplementary Information. [file 41598_2024_54097_MOESM1_ESM.pdf]

Supplementary Information

Vibrio natriegens as a superior host for the production of c-type cytochromes and difficult-to-express redox proteins

Helena Fuchs[1]*, Sophie R. Ullrich[1], Sabrina Hedrich[1]**

[1] TU Bergakademie Freiberg, Institute of Biosciences, Leipziger Straße 29, 09599 Freiberg, Germany

* helena.fuchs@student.tu-freiberg.de

** sabrina.hedrich@bio.tu-freiberg.de

Contents

Supplementary Figures 4

Supplementary Figure 1: Membrane association of rusticyanin and Cytc in expression hosts 4

Supplementary Figure 2: UV/Vis spectra of re-reduced Cytc in the cytoplasmic fractions .. 5

Supplementary Figure 3: Growth curves 6

Supplementary Figure 4: pUC57_cycA vector map 7

Supplementary Figure 5: pUC57_cyc1 vector map 8

Supplementary Figure 6: pUC57_rus vector map 9

Supplementary Figure 7: pUC57_cyc2 vector map 10

Supplementary Figure 8: pET16bP_cycA_cyc1_rus_cyc2 vector map 11

Supplementary Figure 9: Unprocessed images of the immunodetection of rusticyanin in cell compartments (Figure 2 a) 12

Supplementary Figure 10: Unprocessed images of the TMBZ-stain for the detection of holo-c-type cytochromes in cell compartments (Figure 2 b) 13

Supplementary Figure 11: Unprocessed images of the immunodetection of membrane-associated rusticyanin (Supplementary Figure 1 a) 14

Supplementary Figure 12: Unprocessed images of the TMBZ-stain of membrane-associated holo-c-type cytochromes (Supplementary Figure 1 b) 15

Supplementary Figure 13: All UV/Vis spectra of the periplasmic fractions 16

Supplementary Figure 14: All UV/Vis spectra of the cytoplasmic fractions 17

Supplementary Notes 18

Supplementary Note 1: Original and optimized gene and protein sequences 18

CycA2 18

Cyc1A 18

Rus 19

Cyc2A 19

Supplementary Note 2: Prediction results from SignalP6.0 and ProtParam 20

CycA2 20

Cyc1A 20

Rus 20

Cyc2A 21

Supplementary Note 3: Input sequences and results from chromosomal promoter predictions 22

E. coli BL21(DE3) *ccmA-H, napFDAGHBC* complete genome bottom strand (2,186,096 .. 2,198,852) 22

V. natriegens ATCC 14048 *ccmA-I* chromosome1 bottom strand (2,315,980 .. 2,323,459) 24

V. natriegens ATCC 14048 *napFDAB* chromosome 2 bottom strand (1,611,395 ... 1,616,456) 26

V. natriegens ATCC 14048 *napGH* chromosome 2 bottom strand (975,950 .. 978,054) 27
V. natriegens ATCC 14048 *napC* chromosome 2 top strand (1,303,543 .. 1,304,928)...28
Supplementary Tables 29
Supplementary Table 1: Strains, plasmids, and primers 29
Supplementary Table 2: Antibiotic concentrations used for selection 31
Supplementary Table 3: Media recipes 32
20x v2salts 33
25x M 33
50x 5052 33
500x trace metals 33
Supplementary Table 4: Biomass production in 50 mL cultures (cell wet weight)..............34
Supplementary Table 5: BCA assay results 35
Supplementary Data..36
Supplementary Data 1: Plasmid sequences..36
pUC57_cycA sequence 36
pUC57_cyc1 sequence 37
pUC57_rus sequence...38
pUC57_cyc2 sequence 39
pET16bP sequence..41
pET16bP_cycA_cyc1_rus_cyc2 sequence..43
References…………………………………………………………………………………….46

Supplementary Figures

Supplementary Figure 1: Membrane association of rusticyanin and Cytc in expression hosts

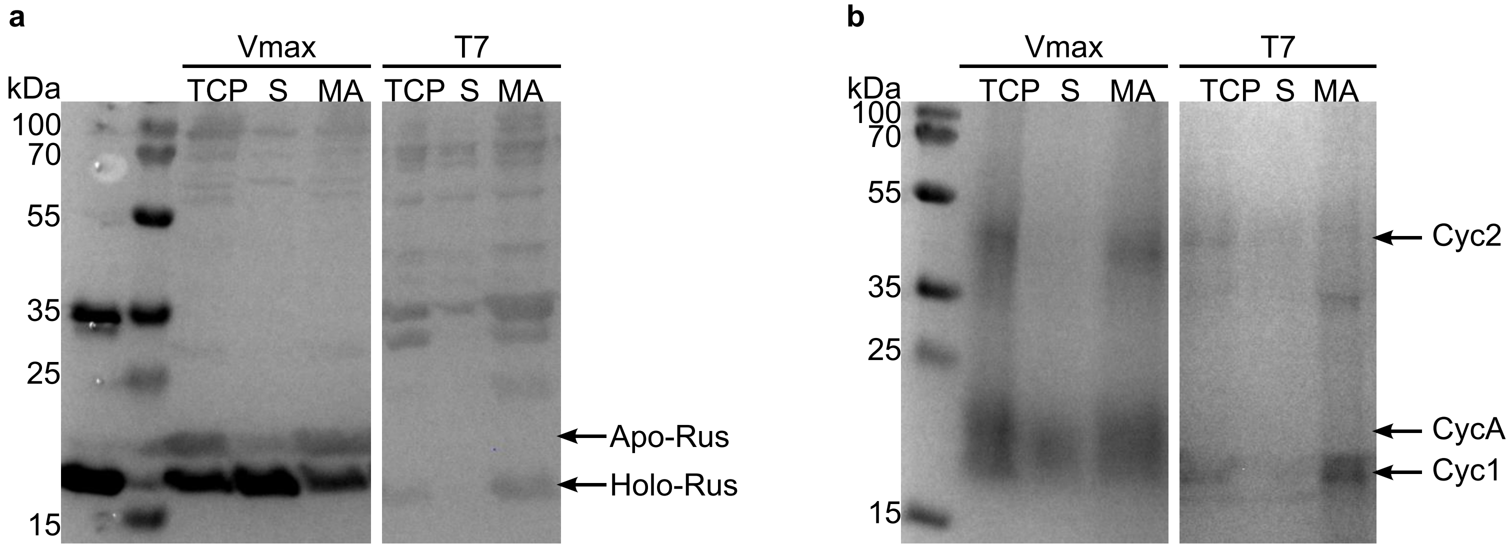

Supplementary Fig. 1. Membrane association of rusticyanin (Rus) and Cytc in expression hosts. Fresh cells were pelleted to an OD_{600} of 10 and disrupted in a vibration mill after lyosozyme treatment (total volume 400 μL). 100 μL were collected immediately after cell disruption (total cellular proteins, TCP), mixed with 50 μL 6x Laemmli buffer and boiled at 95 °C for 5 min. After centrifugation (10,000 xg, 5 min, 4 °C) 100 μL were collected for SDS-PAGE and prepared accordingly (soluble proteins, S). Membrane associated proteins (MA) were collected by washing with 120 μL 1 M Na-acetate (pH 3.5) and prepared for SDS-PAGE accordingly. Samples used for immunodetection of Rus were diluted 1:3 with Laemmli buffer. Samples from Vmax X2 and T7 Express were detected on different membranes/gels. All samples, gels, Western blots, and immunodetections were processed in parallel. **a)** Immunodetection of Rus in protein extracts. A holo-Rus positive control (0.33 μg), isolated from *At. ferrooxidans*, was loaded left to the molecular weight marker. Holo-Rus possesses a molecular weight of 16.6 kDa and apo-Rus 19.9 kDa. **b)** TMBZ-stain according to Thomas et al. [1] of holo-c-type cytochromes in protein extracts. The cytochromes possess the following molecular weights: (i) holo-Cyc1 20.0 kDa, (ii) holo-CycA 22.3 kDa, (iii) holo-Cyc2 49.3 kDa.

Supplementary Figure 2: UV/Vis spectra of re-reduced Cytc in the cytoplasmic fractions

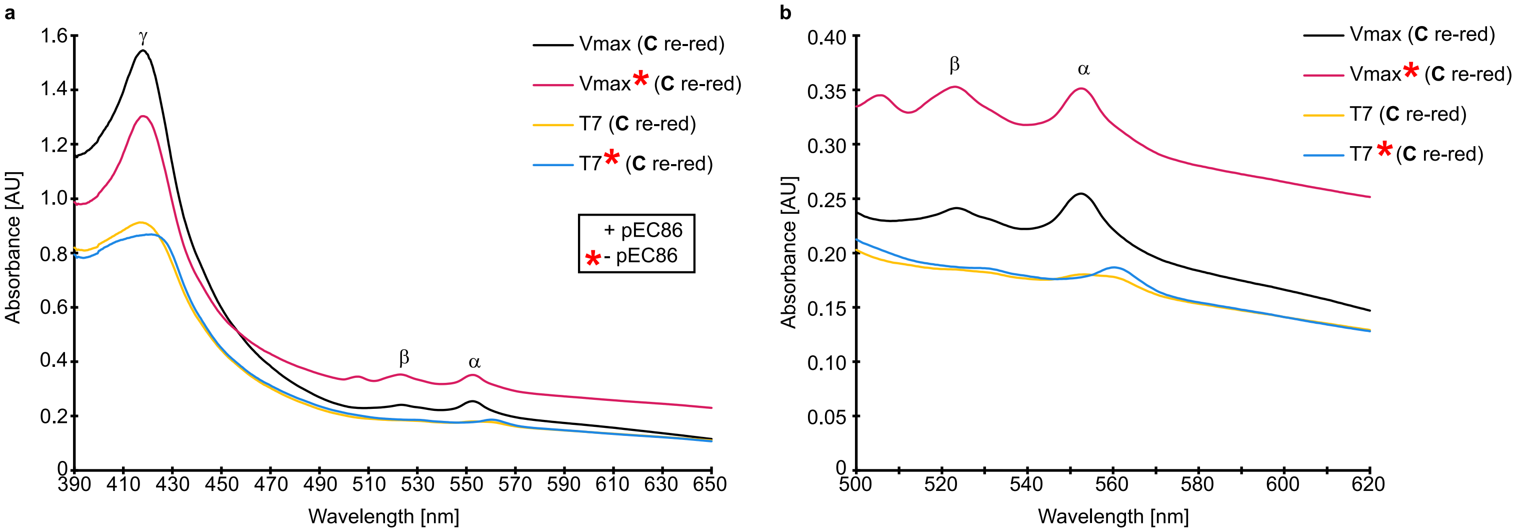

Supplementary Fig. 2. UV/Vis spectra of re-reduced (re-red) Cytc in the cytoplasmic fractions. Fresh cells were fractionated according to Petiti et al. [2]. A 100 µL sample was used for each measurement. Measurements were performed in TES buffer diluted 1:2 in water at pH 8.0. Strains without the pEC86 plasmid are labelled with a red star. Vmax X2 carrying the pEC86 plasmid is coloured in black, Vmax X2 without pEC86 in red, T7 Express with pEC86 in yellow, and T7 Express without pEC86 in blue. The three characteristic absorbance maxima of reduced haem c are labelled α (550 nm), β (525 nm), and γ (410 – 420 nm) respectively. Oxidation was achieved by the addition of up to 10 mM $Na_2[IrCl_6]$, and reduction by adding Na-dithionite and incubating samples on ice for 30 min. **a)** UV/Vis spectra of re-reduced c-type cytochromes in the cytoplasmic protein fraction in the range of 390 – 650 nm. **b)** UV/Vis spectra of re-reduced c-type cytochromes in the cytoplasmic protein fraction in the range of 500 – 620 nm.

Supplementary Figure 3: Growth curves

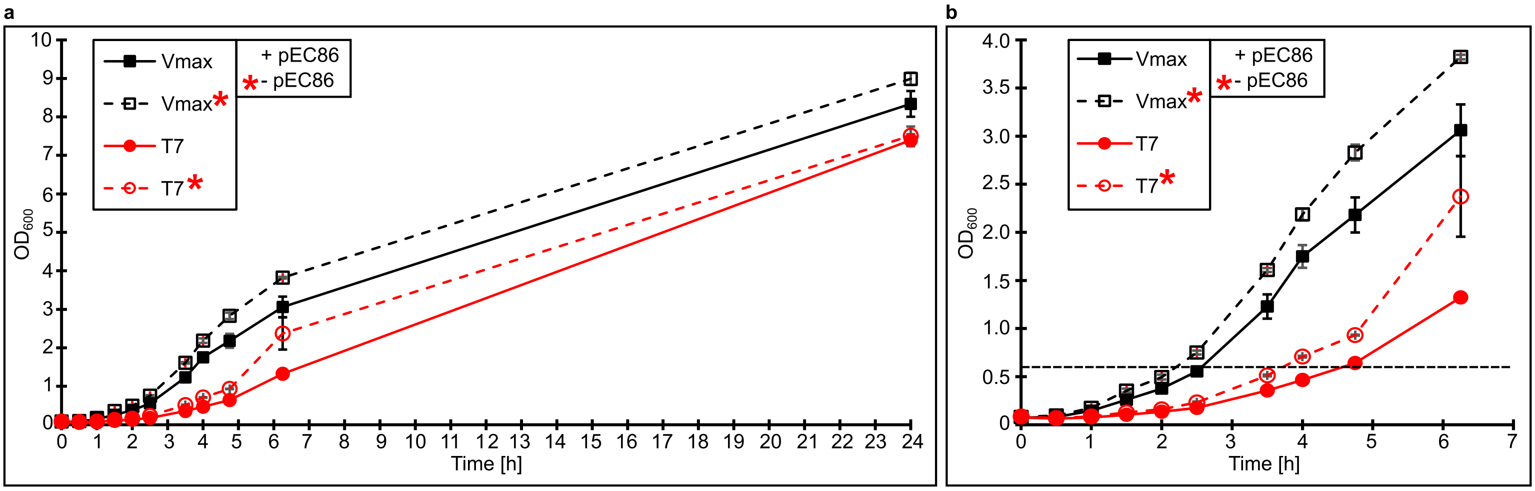

Supplementary Fig. 3. Growth curves of Vmax X2 and T7 Express during expression. All strains carried the pET16bP_cycA_cyc1_rus_cyc2 vector. Strains which did not carry the additional pEC86 vector are marked with a red star. Growth conditions are described in the manuscript. The optical density at 600 nm (OD_{600}) was measured with a Specord 50 Plus spectrometer (Analytik Jena) running WinAspect Plus (Analytik Jena). Growth medium was used for blanking and dilutions. Values are given as the mean of triplicates with the standard deviation as error bars. **a)** Growth curves for the entire duration of the expression. **b)** Growth curves during the initial growth phase. The horizontal dashed line marks the OD_{600} of 0.6 where cultures were transferred from 30 °C to 25 °C (as well as the point of induction for T7 Express cultures).

Supplementary Figure 4: pUC57_cycA vector map

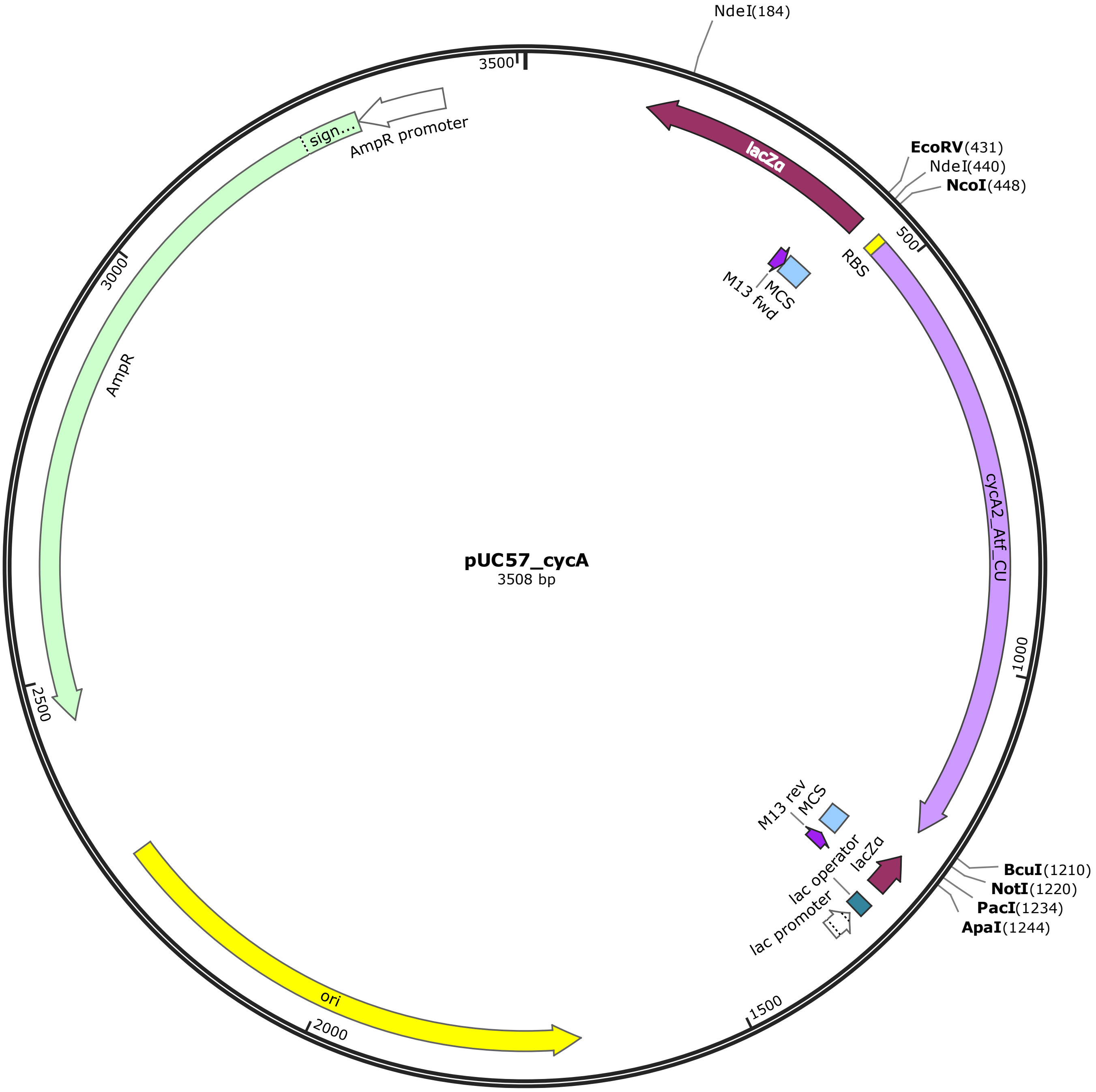

Supplementary Fig. 4. Vector map of the pUC57_cycA plasmid. The coding sequence for the c-type cytochrome CycA2 from *Acidithiobacillus ferrooxidans* CCM4253 was obtained from GenBank (accession number QKQP01000005.1; locus-tag DN052_09955) and codon-optimized for *Escherichia coli* K-12 (termed cycA2_Atf_CU). Restriction sites for *Eco*RV (*Eco*321I), *Nde*I, and *Nco*I as well as the ribosome binding site with linker from the pET16bP vector were added at the 5' end. Restriction sites for *Bcu*I (*Spe*I), *Not*I, *Pac*I, and *Apa*I were added at the 3' end. The optimized gene sequence was synthesized and cloned into the *Eco*RV/*Apa*I restriction site of a pUC57 plasmid by GeneCust (Boynes, France). Control sequencing was performed by GeneCust (Boynes, France). In-silico cloning and plasmid visualization was performed with SnapGene (Dotmatics).

Supplementary Figure 5: pUC57_cyc1 vector map

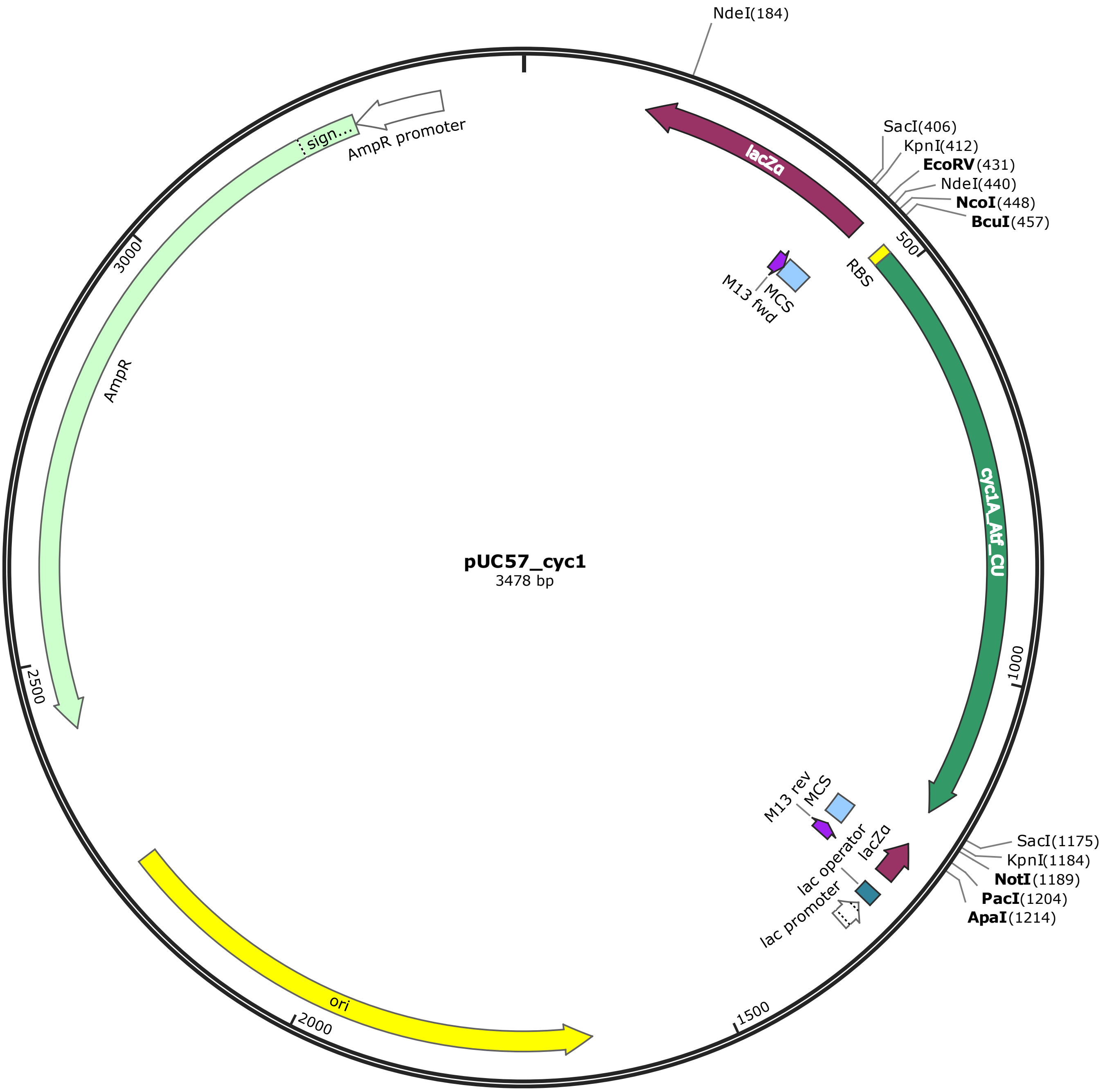

Supplementary Fig. 5. Vector map of the pUC57_cyc1 plasmid. The coding sequence for the c-type cytochrome Cyc1A from *At. ferrooxidans* CCM4253 was obtained from GenBank (accession number QKQP01000005.1; locus-tag DN052_11970) and codon-optimized for *E. coli* K-12 (termed cyc1A_Atf_CU). Restriction sites for *Eco*RV (*Eco*321I), *Nde*I, *Nco*I, and *Bcu*I (*Spe*I) as well as the ribosome binding site with linker from the pET16bP vector were added at the 5' end. Restriction sites for *Sac*I, *Kpn*I, *Not*I, *Pac*I, and *Apa*I were added at the 3' end. The optimized gene sequence was synthesized and cloned into the *Eco*RV/*Apa*I restriction site of a pUC57 plasmid by GeneCust (Boynes, France). Control sequencing was performed by GeneCust (Boynes, France). In-silico cloning and plasmid visualization was performed with SnapGene (Dotmatics).

Supplementary Figure 6: pUC57_rus vector map

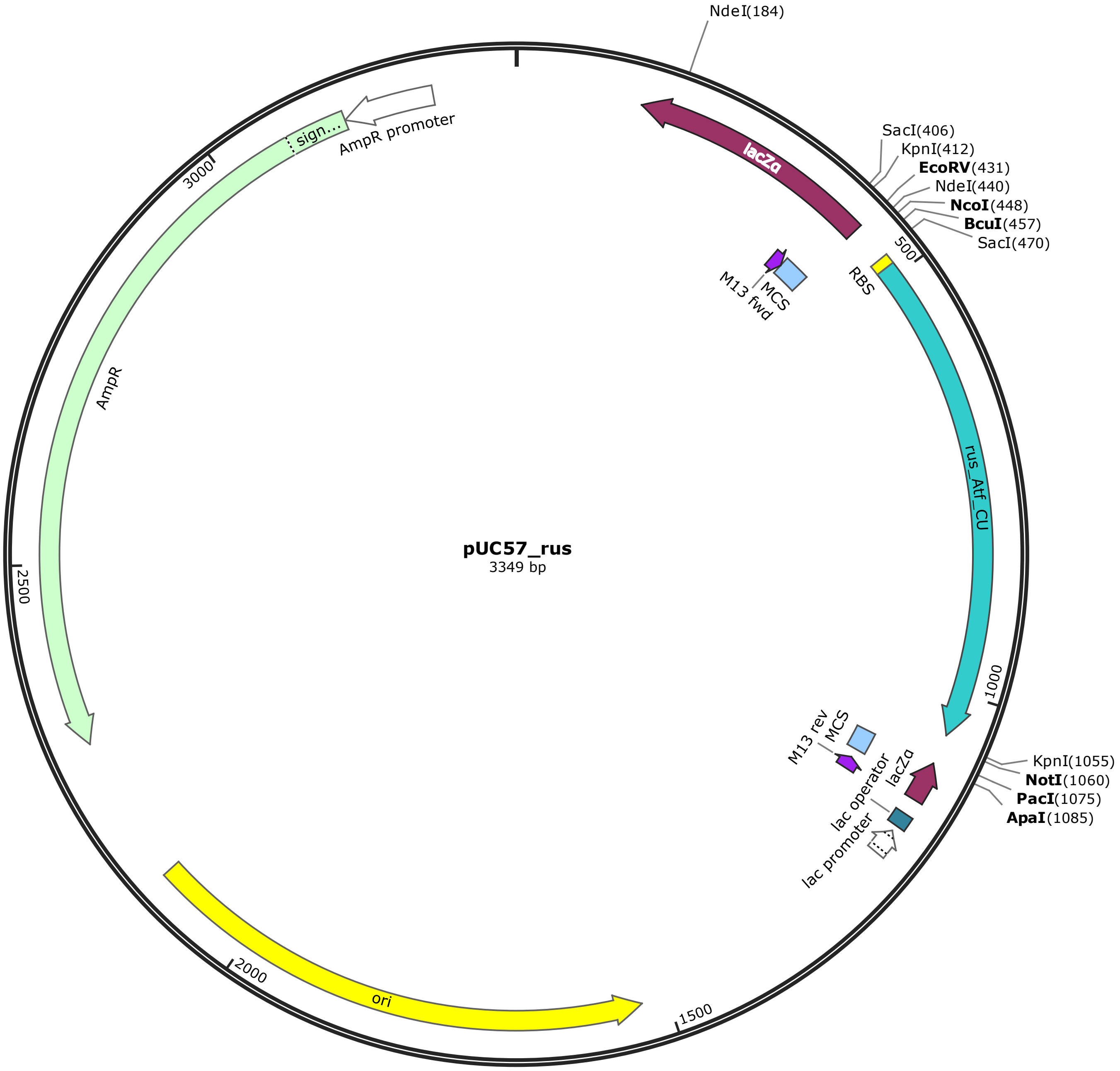

Supplementary Fig. 6. Vector map of the pUC57_rus plasmid. The coding sequence for the periplasmic blue copper protein rusticyanin (Rus) from *At. ferrooxidans* CCM4253 was obtained from GenBank (accession number QKQP01000005.1; locus-tag DN052_11940) and codon-optimized for *E. coli* K-12 (termed rus_Atf_CU). Restriction sites for *Eco*RV (*Eco*321I), *Nde*I, *Nco*I, *Bcu*I (*Spe*I), and *Sac*I as well as the ribosome binding site with linker from the pET16bP vector were added at the 5' end. Restriction sites for *Kpn*I, *Not*I, *Pac*I, and *Apa*I were added at the 3' end. The optimized gene sequence was synthesized and cloned into the *Eco*RV/*Apa*I restriction site of a pUC57 plasmid by GeneCust (Boynes, France). Control sequencing was performed by GeneCust (Boynes, France). In-silico cloning and plasmid visualization was performed with SnapGene (Dotmatics).

Supplementary Figure 7: pUC57_cyc2 vector map

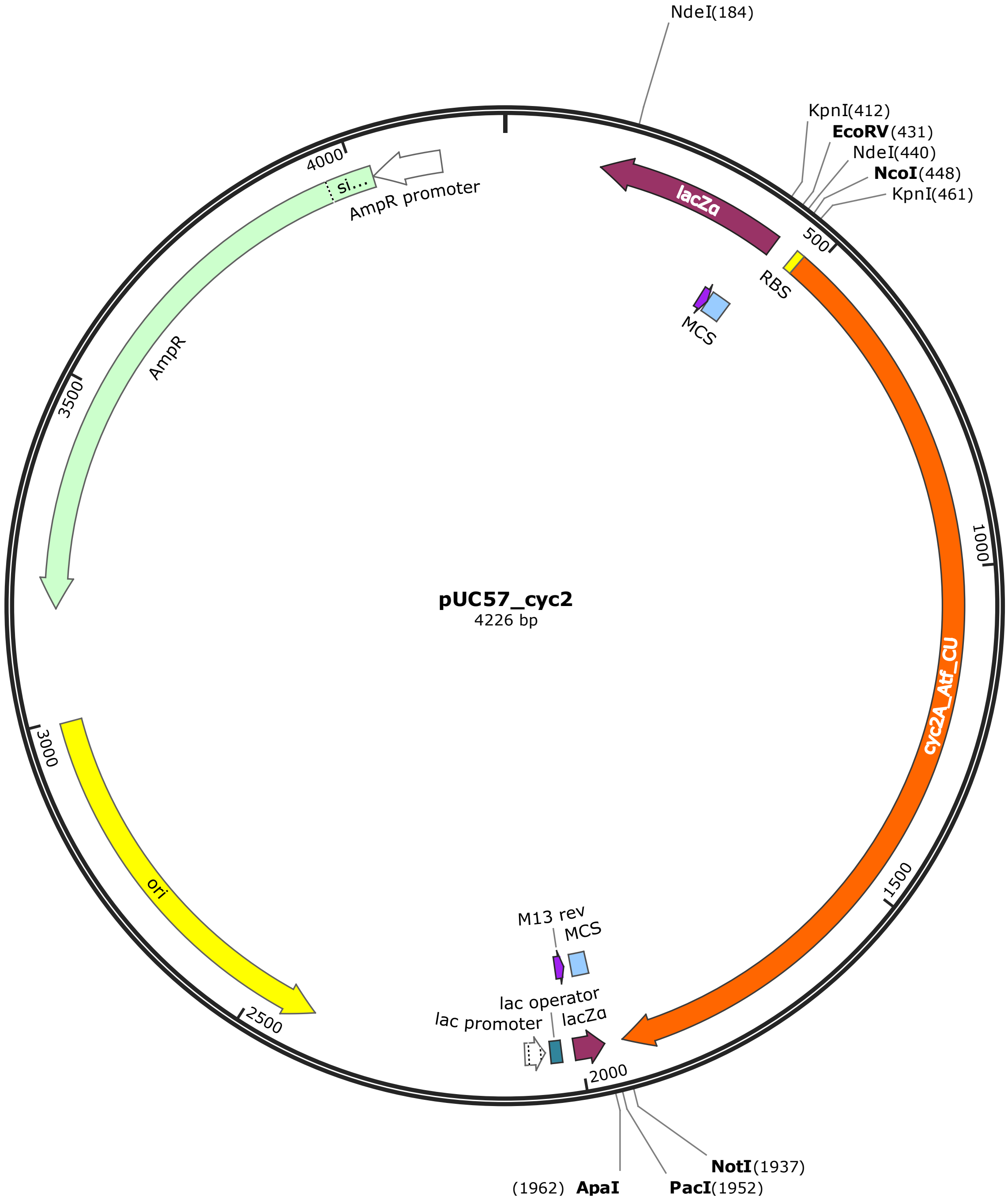

Supplementary Fig. 7. Vector map of the pUC57_cyc2 plasmid. The coding sequence for the c-type cytochrome Cyc2A from *At. ferrooxidans* CCM4253 was obtained from GenBank (accession number QKQP01000005.1; locus-tag DN052_11975) and codon-optimized for *E. coli* K-12 (termed cyc2A_Atf_CU). Restriction sites for *Eco*RV (*Eco*321I), *Nde*I, *Nco*I, and *Kpn*I as well as the ribosome binding site with linker from the pET16bP vector were added at the 5' end. Restriction sites for *Not*I, *Pac*I, and *Apa*I were added at the 3' end. The optimized gene sequence was synthesized and cloned into the *Eco*RV/*Apa*I restriction site of a pUC57 plasmid by GeneCust (Boynes, France). Control sequencing was performed by GeneCust (Boynes, France). In-silico cloning and plasmid visualization was performed with SnapGene (Dotmatics).

Supplementary Figure 8: pET16bP_cycA_cyc1_rus_cyc2 vector map

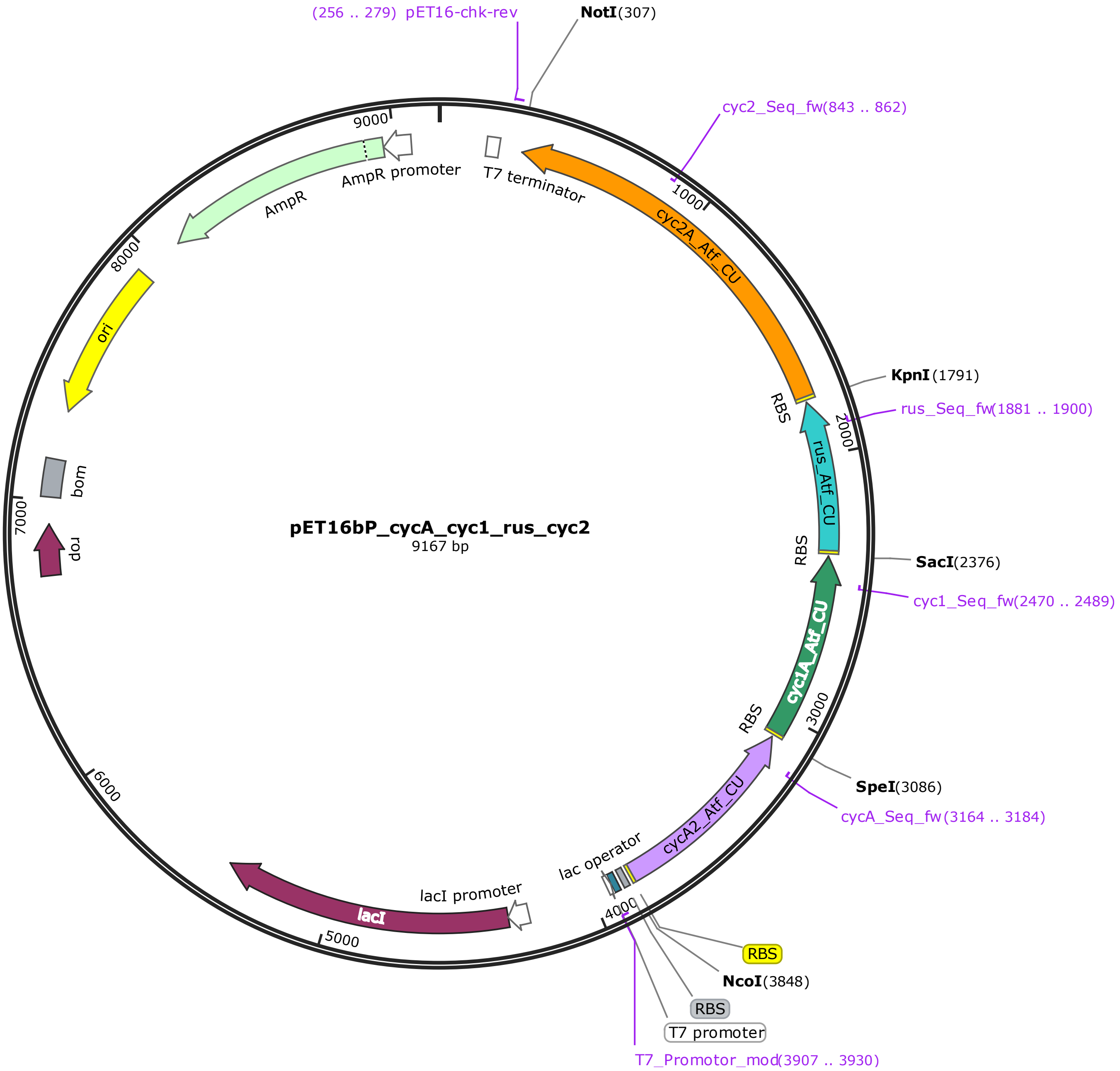

Supplementary Fig. 8. Vector map of the pET16bP_cycA_cyc1_rus_cyc2 plasmid. The pET16bP vector is a modified pET16b vector with an extended multiple cloning site. The four redox protein genes were cloned into the *Nco*I/*Not*I restriction site of the vector by restriction digestion and ligation of the individual genes. Connecting restriction sites are noted. Correct cloning was confirmed by sequencing (Eurofins). Sequencing primers are displayed in purple. Primer sequences can be found in Supplementary Table 1. In-silico cloning and plasmid visualization was performed with SnapGene (Dotmatics).

Supplementary Figure 9: Unprocessed images of the immunodetection of rusticyanin in cell compartments (Figure 2 a)

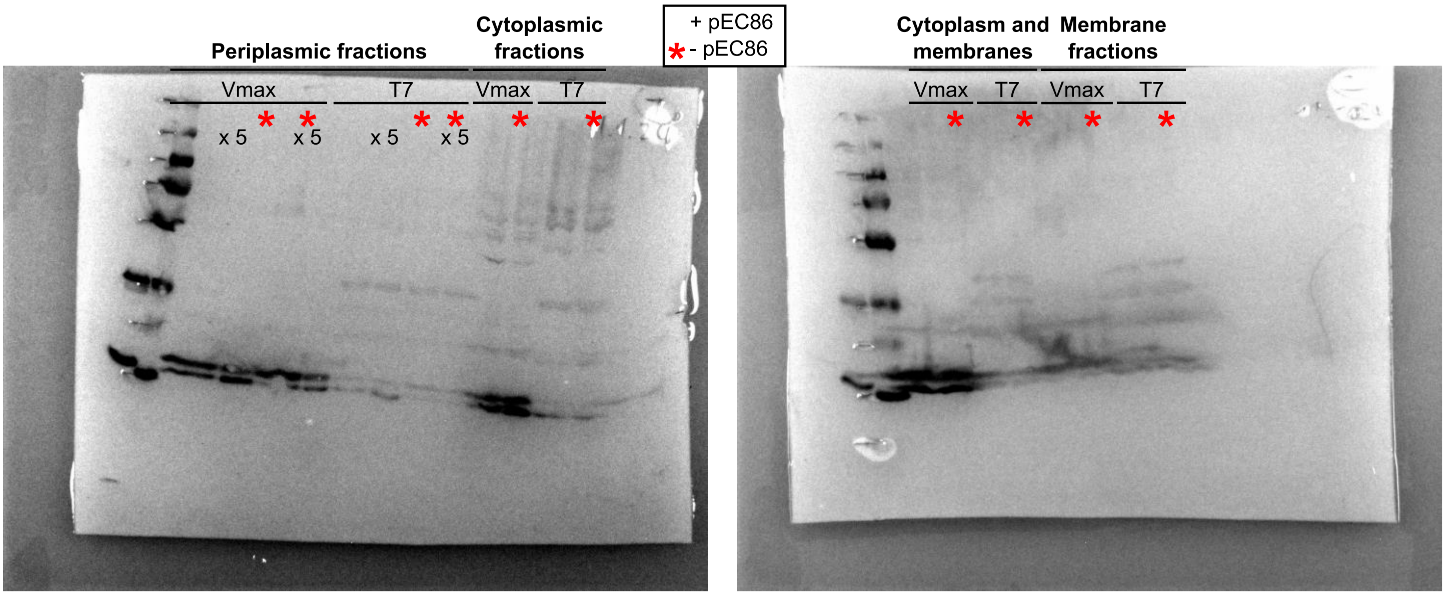

Supplementary Fig. 9. Unprocessed images of the immunodetection of rusticyanin (Rus) in cell compartments (Fig. 2 a). Fresh cells were fractionated according to Petiti et al. [2]. Protein concentrations of each fraction were determined with a BCA assay in triplicates. For unconcentrated fractions, 150 µg total protein was loaded onto SDS-PAGE gels, 300 µg total protein was loaded for the concentrated periplasmic fractions (x 5). All samples, gels, Western blots, and immunodetections were processed in parallel. Samples from cells without the pEC86 plasmid are marked with a red cross. A holo-Rus positive control (0.33 µg), isolated from *At. ferrooxidans*, was loaded left to the molecular weight marker. Holo-Rus possesses a molecular weight of 16.6 kDa and apo-Rus 19.9 kDa. Membranes were visualized with a ChemiDoc XRS+ gel imaging system (Bio-Rad) running ImageLab software (Bio-Rad).

Supplementary Figure 10: Unprocessed images of the TMBZ-stain for the detection of holo-c-type cytochromes in cell compartments (Figure 2 b)

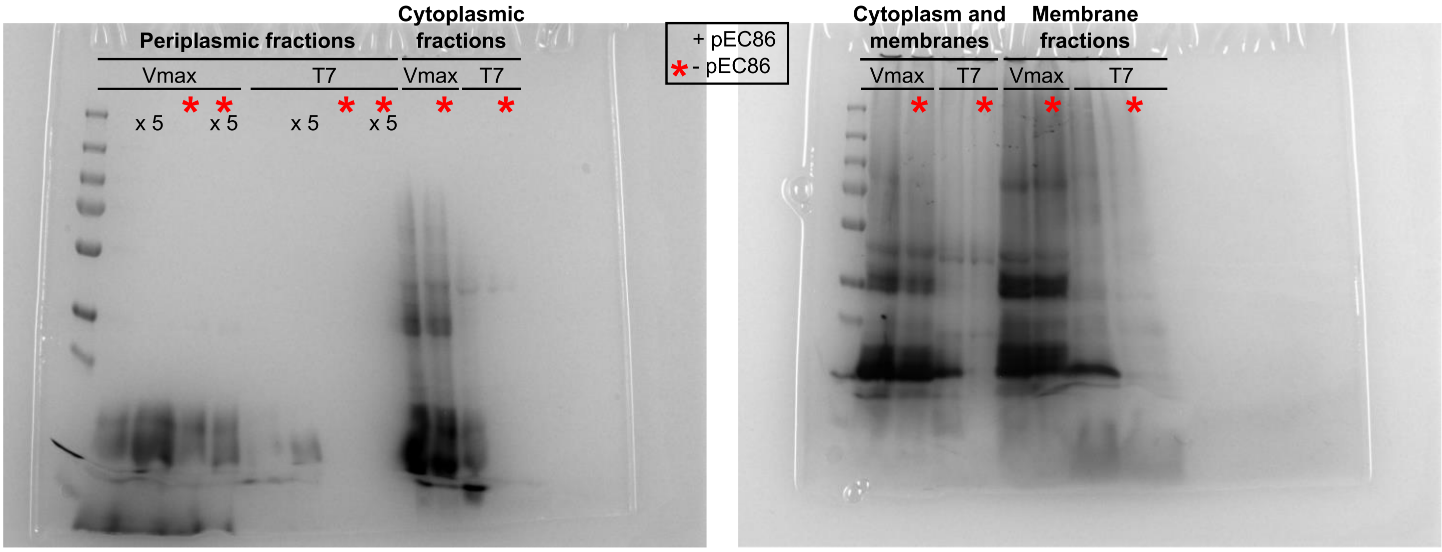

Supplementary Fig. 10. Unprocessed images of the 3,3',5,5'-tetramethylbenzidine (TMBZ) stain for the detection of holo-c-type cytochromes in cell compartments (Fig. 2 b). Fresh cells were fractionated according to Petiti et al. [2]. Protein concentrations of each fraction were determined with a BCA assay in triplicates. For unconcentrated fractions, 150 µg total protein was loaded onto SDS-PAGE gels, 300 µg total protein was loaded for the concentrated periplasmic fractions (x 5). All samples and gels were processed in parallel. TMBZ-staining was performed according to Thomas et al. [1]. Samples from cells without the pEC86 plasmid are marked with a red cross. The cytochromes possess the following molecular weights: (i) holo-Cyc1 20.0 kDa, (ii) holo-CycA 22.3 kDa, (iii) holo-Cyc2 49.3 kDa. Gels were visualized with a ChemiDoc XRS+ gel imaging system (Bio-Rad) running ImageLab software (Bio-Rad).

Supplementary Figure 11: Unprocessed images of the immunodetection of membrane-associated rusticyanin (Supplementary Figure 1 a)

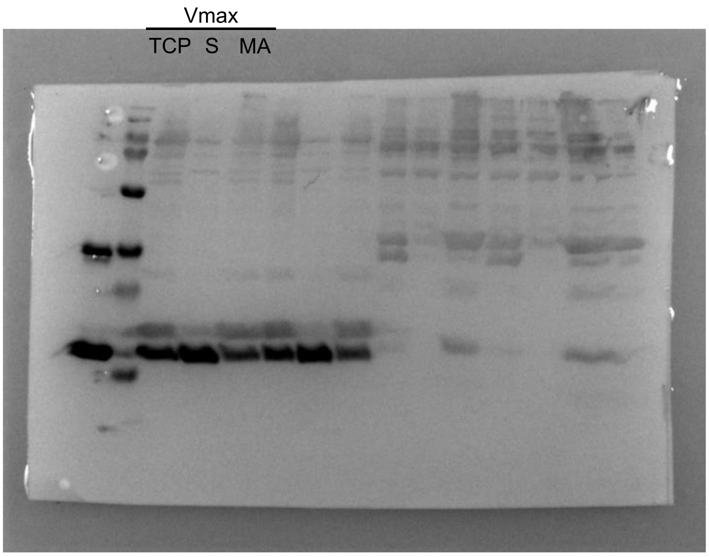

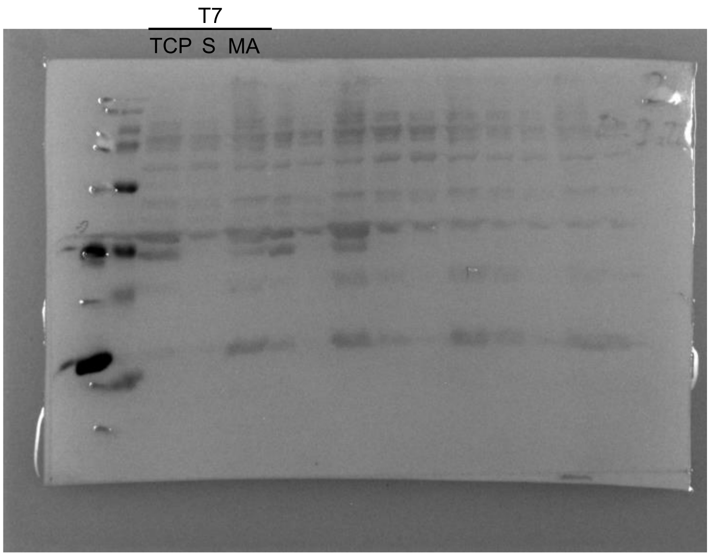

Supplementary Fig. 11. Unprocessed images of the immunodetection of membrane-associated rusticyanin (Supplementary Fig. 1 a). Fresh cells were pelleted to an OD_{600} of 10 and disrupted in a vibration mill after lyosozyme treatment (total volume 400 μL). 100 μL were collected immediately after cell disruption (total cellular proteins, TCP), mixed with 50 μL 6x Laemmli buffer and boiled at 95 °C for 5 min. After centrifugation (10,000 xg, 5 min, 4 °C) 100 μL were collected for SDS-PAGE and prepared accordingly (soluble proteins, S). Membrane-associated proteins (MA) were collected by adding 120 μL 1 M Na-acetate (pH 3.5) and prepared for SDS-PAGE accordingly. Samples used for immunodetection of Rus were diluted 1:3 with Laemmli buffer. Samples from Vmax X2 and T7 Express were detected on different membranes. All samples, gels, Western blots, and immunodetections were processed in parallel. A holo-Rus positive control (0.33 μg), isolated from *At. ferrooxidans*, was loaded left to the molecular weight marker. Holo-Rus possesses a molecular weight of 16.6 kDa and apo-Rus 19.9 kDa. Membranes were visualized with a ChemiDoc XRS+ gel imaging system (Bio-Rad) running ImageLab software (Bio-Rad).

Supplementary Figure 12: Unprocessed images of the TMBZ-stain of membrane-associated holo-c-type cytochromes (Supplementary Figure 1 b)

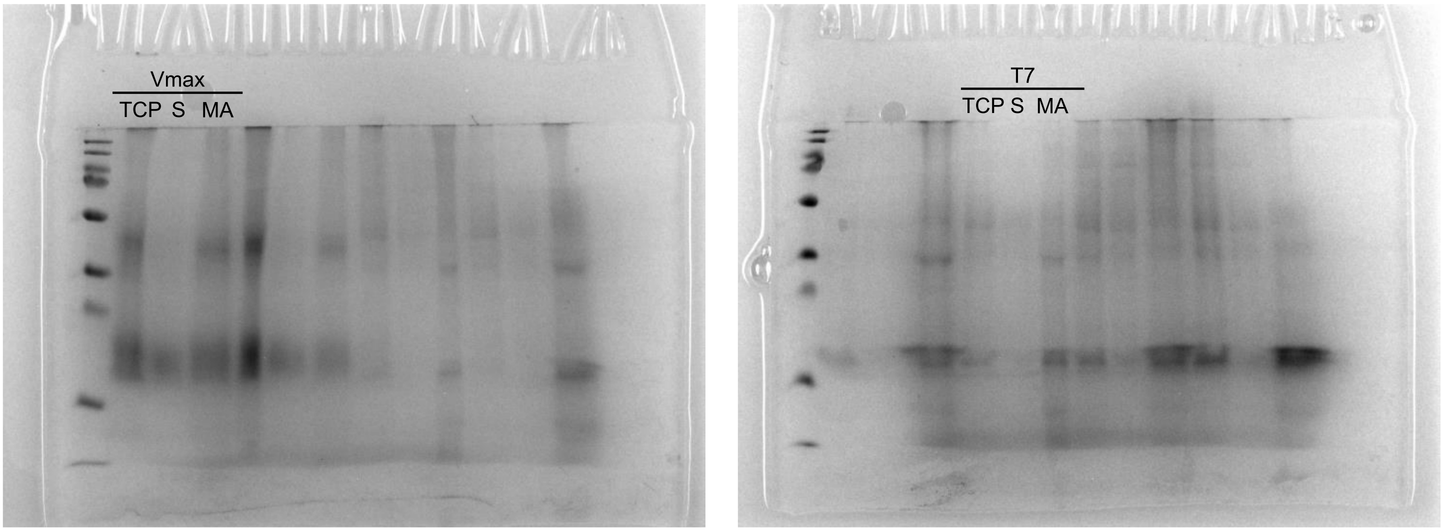

Supplementary Fig. 12. Unprocessed images of the TMBZ-stain of membrane-associated holo-c-type cytochromes (Supplementary Fig. 1 b). Fresh cells were pelleted to an OD_{600} of 10 and disrupted in a vibration mill after lyosozyme treatment (total volume 400 μL). 100 μL were collected immediately after cell disruption (total cellular proteins, TCP), mixed with 50 μL 6x Laemmli buffer and boiled at 95 °C for 5 min. After centrifugation (10,000 xg, 5 min, 4 °C) 100 μL were collected for SDS-PAGE and prepared accordingly (soluble proteins, S). Membrane-associated proteins (MA) were collected by adding 120 μL 1 M Na-acetate (pH 3.5) and prepared for SDS-PAGE accordingly. Samples from Vmax X2 and T7 Express were detected on different gels. All samples and gels were processed in parallel. TMBZ-staining was performed according to Thomas et al.[1]. The cytochromes possess the following molecular weights: (i) holo-Cyc1 20.0 kDa, (ii) holo-CycA 22.3 kDa, (iii) holo-Cyc2 49.3 kDa. Gels were visualized with a ChemiDoc XRS+ gel imaging system (Bio-Rad) running ImageLab software (Bio-Rad).

Supplementary Figure 13: All UV/Vis spectra of the periplasmic fractions

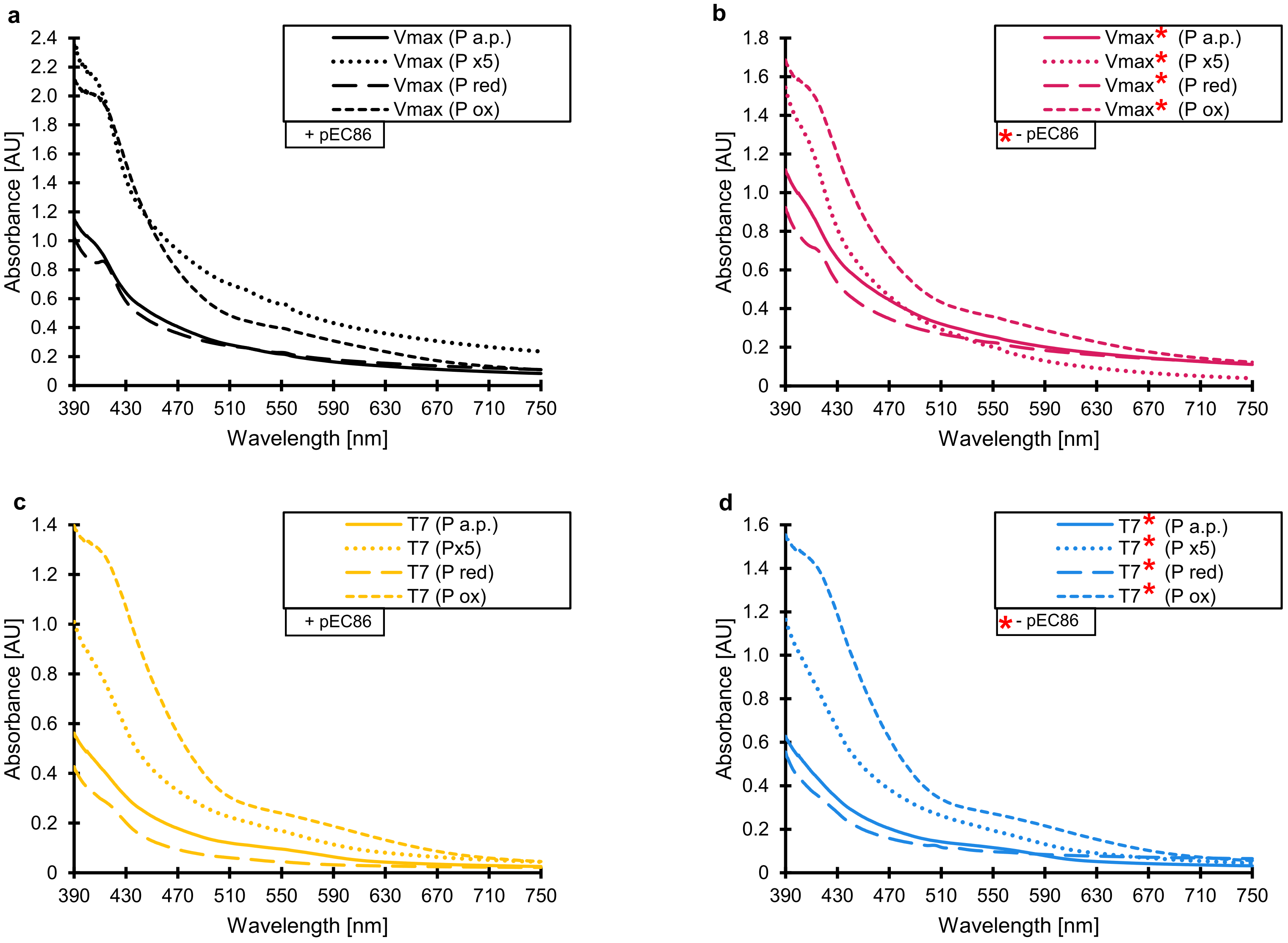

Supplementary Fig. 13. All UV/Vis spectra of the periplasmic fractions of Vmax X2 and T7 Express. Fresh cells were fractionated according to Petiti et al. [2]. A 100 µL sample was used for each measurement. Measurements were performed in TES buffer diluted 1:2 in water at pH 8.0. Strains without the pEC86 plasmid are labelled with a red star. Vmax X2 carrying the pEC86 plasmid is coloured in black, Vmax X2 without pEC86 in red, T7 Express with pEC86 in yellow, and T7 Express without pEC86 in blue. The three characteristic absorbance maxima of reduced haem c are labelled α (550 nm), β (525 nm), and γ (410 – 420 nm) respectively. Oxidation was achieved by the addition of up to 10 mM $Na_2[IrCl_6]$, and reduction by adding Na-dithionite and incubating samples on ice for 30 min. Legend: P – periplasmic fraction; a.p. – as prepared (diluted TES buffer); x5 – concentrated five times; red – reduced; ox – oxidized. **a)** Spectra of the periplasmic fraction of Vmax X2 + pEC86 + pET16_cycA_cyc1_rus_cyc2. **b)** Spectra of the periplasmic fraction of Vmax X2 + pET16_cycA_cyc1_rus_cyc2. **c)** Spectra of the periplasmic fraction of T7 Express + pEC86 + pET16_cycA_cyc1_rus_cyc2. **d)** Spectra of the periplasmic fraction of T7 Express + pET16_cycA_cyc1_rus_cyc2.

Supplementary Figure 14: All UV/Vis spectra of the cytoplasmic fractions

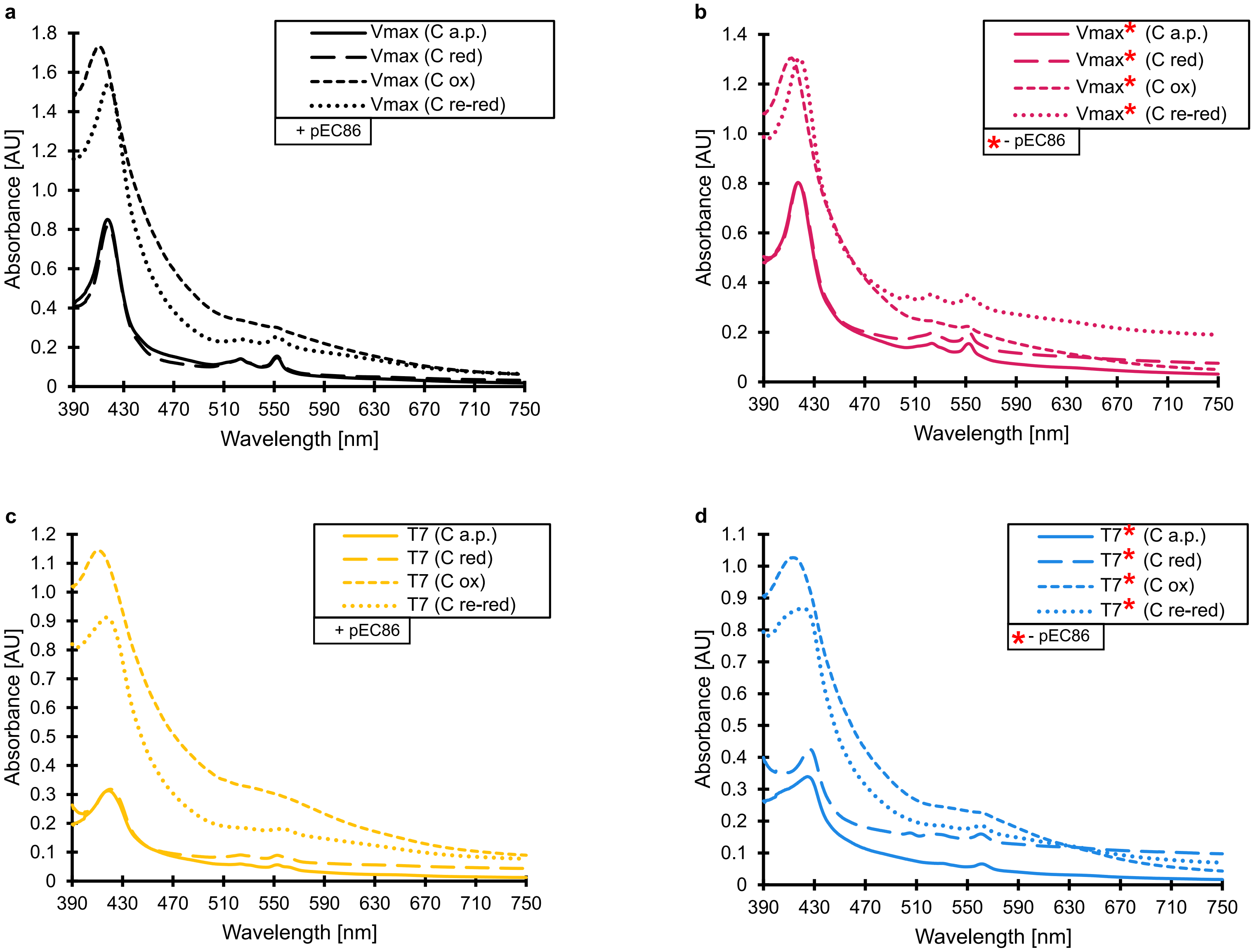

Supplementary Fig. 14. All UV/Vis spectra of the periplasmic fractions of Vmax X2 and T7 Express. Fresh cells were fractionated according to Petiti et al. [2]. A 100 μL sample was used for each measurement. Measurements were performed in TES buffer diluted 1:2 in water at pH 8.0. Strains without the pEC86 plasmid are labelled with a red star. Vmax X2 carrying the pEC86 plasmid is coloured in black, Vmax X2 without pEC86 in red, T7 Express with pEC86 in yellow, and T7 Express without pEC86 in blue. The three characteristic absorbance maxima of reduced haem c are labelled α (550 nm), β (525 nm), and γ (410 – 420 nm) respectively. Oxidation was achieved by the addition of up to 10 mM $Na_2[IrCl_6]$, and reduction by adding Na-dithionite and incubating samples on ice for 30 min. Legend: C – cytoplasmic fraction; a.p. – as prepared (diluted TES buffer); red – reduced; ox – oxidized; re-red – re-reduced by adding $Na_2[IrCl_6]$ to the oxidized sample. **a)** Spectra of the cytoplasmic fraction of Vmax X2 + pEC86 + pET16_cycA_cyc1_rus_cyc2. **b)** Spectra of the cytoplasmic fraction of Vmax X2 + pET16_cycA_cyc1_rus_cyc2. **c)** Spectra of the cytoplasmic fraction of T7 Express + pEC86 + pET16_cycA_cyc1_rus_cyc2. **d)** Spectra of the cytoplasmic fraction of T7 Express + pET16_cycA_cyc1_rus_cyc2.

Supplementary Notes

Supplementary Note 1: Original and optimized gene and protein sequences

All original gene and protein sequences of CycA2, Cyc1A, Rus, and Cyc2A from *At. ferrooxidans* CCM4253 were obtained from GenBank (accession number QKQP01000005.1). The following locus-tags were used for the original gene sequences:

- CycA2: DN052_09955
- Cyc1A: DN052_11970
- Rus: DN052_11940
- Cyc2A: DN052_11975

All gene sequences were codon-optimized for *E. coli* K-12 using JCat [3] (http://www.jcat.de/Start.jsp). Restriction sites for the following restriction endonucleases were excluded: *Eco*RV (*Eco*32I), *Nde*I, *Nco*I, *Bcu*I (*Spe*I), *Sac*I, *Aat*I, *Not*I, *Pac*I, *Kpn*I, and *Apa*I. Optimized gene sequences and the translated protein sequences are listed below:

CycA2

```
ATGCCGGCTCTGACCACCAAAGTTCCGCACATGCTGCAGAAAAAACCGCGTCTGTCTGTTATCCCGCTGTTCTCT
ATGGCTATGGTTATGGCTTCTGCTGCTTACGGTTCTCCGCTGGCTCAGACCGTTTCTGCTGCTCCGGCTGCTGCT
ACCGCTTCTGCTGTTTCTGCTACCGCTGCTACCGCTTCTGTTACCGCTTCTGGTATCCCGACCGTTGTTCAGTCT
ACCTGCATGGCTTGCCACGGTATGCAGGGTATCGCTGCTGACGGTGGTATGTTCCCGAACCTGGCTGGTCAGTGG
AAACCGTACCTGCTGCGTCAGCTGGACCACTTCAAAACCCACGTTCGTGCTGACCCGCAGTCTCCGATCATGTGG
GGTATGGCTGCTCCGCTGACCGCTGCTCAGATGCAGCAGGTTGCTGACTACTTCTCTTCTCAGAAACCGGCTTCT
GGTCACGTTTACGACCCGAAACTGGTTGCTGAAGGTAAAAAACTGTACTTCGGTGGTCTGCCGGACAAACACATG
CCGGCTTGCATGGCTTGCCACGGTGCTACCCTGGCTGGTCTGCCGCCGTACTTCCCGCGTCTGGCTGGTCAGAAA
CGTACCTACGTTATCAACCAGCTGACCTACTTCAAATCTGGTCAGCGTGTTGCTACCCACAAAGGTATCATGCAG
TACGTTGCTTCTCGTCTGAACCCGAAACAGATCACCGCTCTGGCTGCTTACATCCGTTCTCGTTAA
```

GC content: 55,2 %

CAI: 1,00

```
MPALTTKVPHMLQKKPRLSVIPLFSMAMVMASAAYGSPLAQTVSAAPAAATASAVSATAATASVTASGIPTVVQS
TCMACHGMQGIAADGGMFPNLAGQWKPYLLRQLDHFKTHVRADPQSPIMWGMAAPLTAAQMQQVADYFSSQKPAS
GHVYDPKLVAEGKKLYFGGLPDKHMPACMACHGATLAGLPPYFPRLAGQKRTYVINQLTYFKSGQRVATHKGIMQ
YVASRLNPKQITALAAYIRSR
```

Cyc1A

```
ATGACCACCTACCTGTCTCAGGACCGTCTGCGTAACAAAGAAAACGACACCATGACCCACCCGCACTCTAAAATG
TACAAATCTAAAACCTTCCTGCTGTTCTCTGCTCTGCTGCTGGTTGCTGGTCAGGCTTCTGCTGCTGTTGGTTCT
GCTGACGCTCCGGCTCCGTACCGTGTTTCTTCTGACTGCATGGTTTGCCACGGTATGACCGGTCGTGACACCCTG
TACCCGATCGTTCCGCGTCTGGCTGGTCAGCACAAATCTTACCTGGAAGCTCAGCTGAAAGCTTACAAAGACCAC
TCTCGTGCTGACCAGAACGGTGAAATCTACATGTGGCCGGTTGCTCAGGCTCTGGACTCTGCTAAAATCACCGCT
CTGGCTGACTACTTCAACGCTCAGAAACCGCCGATGCAGTCTTCTGGTATCAAACACGCTGGTGTTAAAGAAGGT
AAAGCTATCTTCAACCAGGGTGTTACCAACGAACAGATCCCGGCTTGCATGGAATGCCACGGTTCTGCTGGTCAG
GGTGCTGGTCCGTTCCCGCGTCTGGCTGGTCAGCGTTACGGTTACATCATCCAGCAGCTGACCTACTTCCACAAC
GGTACTCGTGTTAACACCCTGATGAACCAGATCGCTAAAAACATCACCGTTGCTCAGATGAAAGACGTTGCTGCT
TACCTGTCTTCTCTGTAA
```

GC content: 52,1 %

CAI: 1,0

MTTYLSQDRLRNKENDTMTHPHSKMYKSKTFLLFSALLLVAGQASAAVGSADAPAPYRVSSDCMVCHGMTGRDTL
YPIVPRLAGQHKSYLEAQLKAYKDHSRADQNGEIYMWPVAQALDSAKITALADYFNAQKPPMQSSGIKHAGVKEG
KAIFNQGVTNEQIPACMECHGSAGQGAGPFPRLAGQRYGYIIQQLTYFHNGTRVNTLMNQIAKNITVAQMKDVAA
YLSSL

Rus

ATGTACACCCAGAACACCATGAAAAAAAACTGGTACGTTACCGTTGGTGCTGCTGCTGCTCTGGCTGCTACCGTT
GGTATGGGTACTGCTATGGCTGGTACTCTGGACACCACCTGGAAAGAAGCTACCCTGCCGCAGGTTAAAGCTATG
CTGGAAAAAGACACCGGTAAAGTTTCTGGTGACACCGTTACCTACTCTGGTAAAACCGTTCACGTTGTTGCTGCT
GCTGTTCTGCCGGGTTTCCCGTTCCCGTCTTTCGAAGTTCACGACAAAAAAAACCCGACCCTGGAAATCCCGGCT
GGTGCTACCGTTGACGTTACCTTCATCAACACCAACAAAGGTTTCGGTCACTCTTTCGACATCACCAAAAAAGGT
CCGCCGTACGCTGTTATGCCGGTTATCGACCCGATCGTTGCTGGTACTGGTTTCTCTCCGGTTCCGAAAGACGGT
AAATTCGGTTACACCGACTTCACCTGGCACCCGACCGCTGGTACTTACTACTACGTTTGCCAGATCCCGGGTCAC
GCTGCTACCGGTATGTTCGGTAAAATCGTTGTTAAATAA

GC content: 50,7 %

CAI: 0,99

MYTQNTMKKNWYVTVGAAAALAATVGMGTAMAGTLDTTWKEATLPQVKAMLEKDTGKVSGDTVTYSGKTVHVVAA
AVLPGFPFPSFEVHDKKNPTLEIPAGATVDVTFINTNKGFGHSFDITKKGPPYAVMPVIDPIVAGTGFSPVPKDG
KFGYTDFTWHPTAGTYYYVCQIPGHAATGMFGKIVVK

Cyc2A

ATGGTTTCTTCTTCTGTTGGTTTCAAAAAAAAACGTCTGATCGTTGCTCTGGCTGCTGTTGGTGGTATGGCTCTG
TCTTCTGGTGCTTGGGCTCTGCCGTCTTTCGCTCGTCAGACCGGTTGGTCTTGCGCTGCTTGCCACACCTCTTAC
CCGCAGCTGACCCCGATGGGTCGTATGTTCAAACTGCTGGGTTTCACCACCACCAACCTGCAGCGTCAGCAGAAA
CTGCAGGCTAAATTCGGTAACTCTGTTGGTCTGCTGATCTCTCGTGTTTCTCAGTTCTCTATCTTCCTGCAGGCT
TCTGCTACCAACGTTGGTGGTGGTCAGGCTGTTTTCGGTCCGGGTAACTCTAACGCTGGTGCTTCTCCGAACAAC
AACGTTCAGTTCCCGCAGCAGGTTTCTCTGTTCTACGCTGGTGAAATCACCCCGCACATCGGTTCTTTCCTGCAC
CTGACCTACTCTGGTGGTGGTTCTGGTGCTGGTGCTGGTGGTTTCTCTTTCGACGACTCTTCTATCGTTTGGACC
CACCCGTGGAAACTGGGTACTAACAACCTGCTGGTTACCGGTGTTGACGTTAACAACACCCCGACCGCTATGGAC
CTGTGGAACACCACCCCGGACTGGCAGGCTCCGTTCTTCTCTTCTGACTACTCTTCTTGGGGTCACGTTCCGCAG
CCGTTCATCGAATCTTCTGCTGGTGCTGGTTACCCGCTGGCTGGTGTTGGTGTTTACGGTGCTGACATCTTCGGT
CCGAACCGTGCTAACTGGCTGTACGCTGACGCTGACGTTTACACCAACGGTCAGGGTACTCAGGTTAACCCGGTT
GGTGGTTTCACCGCTGCTGGTCCGCAGGGTCGTCTGTCTGGTGGTGCTCCGTACGTTCGTCTGGCTTACCAGCAC
GACTGGGGTGACTGGAACTGGGAAGTTGGTACTTTCGGTATGTGGTCTTCTGTTTACGACAACACCATCAACAAC
ACCCTGAACAAAGCTGGTGGTCCGATCGACACCTTCGACGACTACGACCTGGACACCCAGCTGCAGTGGCTGGAC
ACCAACGACAACAACAACGTTACCATCCGTGCTGCTTGGGTTAACGAACAGCAGCAGTTCGGTGCTGGTAACGTT
ATCTCTTCTAACTCTTCTGGTAACCTGAACTTCTTCAACATCAACGCTACCTACTGGTATCACGACCACTACGGT
ATCCAGGGTGGTTACCGTAACGTTTGGGGTTCTGCTAACCCGGGTCTGTACGGTACTACCTACACCAACTCTGGT
TCTCCGGACACCTCTAACGAATGGATCGAAGCTTCTTACCTGCCGTGGTGGAACACCCGTTTCTCTCTGCGTTAC
GTTGTTTACAACAAATTCAACGGTGTTGGTTCTGCTTCTTCTAACAACCTGGGTTACGGTGCTTCTGCTTACAAC
ACCCTGGAACTGCTGGCTTGGATCTCTTACTAA

GC content: 53,0 %

CAI: 0,99

MVSSSVGFKKKRLIVALAAVGGMALSSGAWALPSFARQTGWSCAACHTSYPQLTPMGRMFKLLGFTTTNLQRQQK
LQAKFGNSVGLLISRVSQFSIFLQASATNVGGGQAVFGPGNSNAGASPNNNVQFPQQVSLFYAGEITPHIGSFLH
LTYSGGGSGAGAGGFSFDDSSIVWTHPWKLGTNNLLVTGVDVNNTPTAMDLWNTTPDWQAPFFSSDYSSWGHVPQ
PFIESSAGAGYPLAGVGVYGADIFGPNRANWLYADADVYTNGQGTQVNPVGGFTAAGPQGRLSGGAPYVRLAYQH
DWGDWNWEVGTFGMWSSVYDNTINNTLNKAGGPIDTFDDYDLDTQLQWLDTNDNNNVTIRAAWVNEQQQFGAGNV
ISSNSSGNLNFFNINATYWYHDHYGIQGGYRNVWGSANPGLYGTTYTNSGSPDTSNEWIEASYLPWWNTRFSLRY
VVYNKFNGVGSASSNNLGYGASAYNTLELLAWISY

Supplementary Note 2: Prediction results from SignalP6.0 and ProtParam

Signal peptides for all protein sequences (Supplementary Note 1) were predicted with SignalP6.0 [4] (https://services.healthtech.dtu.dk/services/SignalP-6.0/) using the "Fast" setting. Protein properties were predicted for apo- and holo-proteins alike with ProtParam [5] (https://web.expasy.org/protparam/).

CycA2

A Sec/SPI signal peptide was predicted with the cleavage site at amino acid 36/37 (85.9%). Important predicted parameters for the apo- and holo-protein are listed below:

	**Apo-protein**	**Holo-protein**
**Amino acid number**	246	210
**Molecular weight [kDa]**	26.2	22.3
**pI**	9.95	9.78

Cyc1A

A Sec/SPI signal peptide was predicted with the cleavage site at amino acid 46/47 (95.73%). Important predicted parameters for the apo- and holo-protein are listed below:

	**Apo-protein**	**Holo-protein**
**Amino acid number**	230	184
**Molecular weight [kDa]**	25.2	20.0
**pI**	9.20	8.86

Rus

A Sec/SPI signal peptide was predicted with the cleavage site at amino acid 32/33 (96.92%). Important predicted parameters for the apo- and holo-protein are listed below:

	**Apo-protein**	**Holo-protein**
**Amino acid number**	187	155
**Molecular weight [kDa]**	19.9	16.6
**pI**	8.85	8.00

Cyc2A

A Sec/SPI signal peptide was predicted with the cleavage site at amino acid 31/32 (97.00%). Important predicted parameters for the apo- and holo-protein are listed below:

	**Apo-protein**	**Holo-protein**
**Amino acid number**	485	454
**Molecular weight [kDa]**	52.4	49.3
**pI**	5.54	4.99

Supplementary Note 3: Input sequences and results from chromosomal promoter predictions

Bacterial promoters were predicted with BPROM [6] (http://www.softberry.com/berry.phtml?topic=bprom&group=programs&subgroup=gfindb) using only the operon sequence with its upstream region as input. Genomic sequences for *Vibrio natriegens* ATCC 14048 (accession number NZ_CP016345 and NZ_CP016346) and *Escherichia coli* BL21(DE3) (accession number NZ_CP053602) were retrieved from GenBank. Results are listed below. All queries were entered in 5' – 3' direction. Regions are noted in brackets. Predicted promoter regions directly upstream of the operons are highlighted with red boxes.

E. coli *BL21(DE3)* ccmA-H, napFDAGHBC *complete genome bottom strand (2,186,096 .. 2,198,852)*

Length of sequence: 12757 bp

Threshold for promoters: 0.20

Number of predicted promoters: 25

```
Promoter Pos:  12689 LDF-  6.85
-10 box at pos.  12674 acgtaggcc Score    -6
-35 box at pos.  12654 ttcaat    Score    35
Promoter Pos:    209 LDF-  5.38
-10 box at pos.    194 aaatatatt Score    43
-35 box at pos.    176 gtgaat    Score    22
Promoter Pos:   6278 LDF-  4.12
-10 box at pos.   6263 gggtatgct Score    65
-35 box at pos.   6245 ttaaca    Score    43
Promoter Pos:   5654 LDF-  3.33
-10 box at pos.   5639 ggttattat Score    76
-35 box at pos.   5615 atcaaa    Score    10
Promoter Pos:   9948 LDF-  3.03
-10 box at pos.   9933 cggtatgtt Score    54
-35 box at pos.   9914 ttgcgt    Score    33
Promoter Pos:   5163 LDF-  2.61
-10 box at pos.   5148 atttacaat Score    65
-35 box at pos.   5129 atgtct    Score    18
Promoter Pos:   6854 LDF-  2.60
-10 box at pos.   6839 aagtaaaat Score    68
-35 box at pos.   6819 cagccg    Score   -17
Promoter Pos:  11945 LDF-  2.43
-10 box at pos.  11930 ggatatatt Score    59
-35 box at pos.  11909 gtgcag    Score    16
Promoter Pos:   7366 LDF-  2.24
-10 box at pos.   7351 tggtgtgct Score    30
-35 box at pos.   7328 ttgaca    Score    66
Promoter Pos:  11412 LDF-  2.22
-10 box at pos.  11396 cggtatcat Score    72
-35 box at pos.  11379 ttcctt    Score    28
Promoter Pos:    548 LDF-  2.12
-10 box at pos.    533 ctgtattaa Score    31
-35 box at pos.    515 ttgcac    Score    33
Promoter Pos:   1961 LDF-  1.72
-10 box at pos.   1946 ggtgatcct Score    26
```

```
-35 box at pos.   1926 tttacg    Score    41
Promoter Pos:   1595 LDF-  1.66
-10 box at pos.   1580 cggtatgtt Score    54
-35 box at pos.   1555 tgaaag    Score    -6
Promoter Pos:   8417 LDF-  1.10
-10 box at pos.   8402 cgttattcc Score    27
-35 box at pos.   8377 ttgtct    Score    48
Promoter Pos:   7854 LDF-  1.07
-10 box at pos.   7839 gatgaaaat Score    33
-35 box at pos.   7819 tttatt    Score    34
Promoter Pos:   8769 LDF-  1.06
-10 box at pos.   8754 cgctaaaag Score    39
-35 box at pos.   8735 gtgcag    Score    16
Promoter Pos:   3925 LDF-  0.96
-10 box at pos.   3910 cgccatatt Score    38
-35 box at pos.   3886 tggcct    Score    19
Promoter Pos:   9394 LDF-  0.93
-10 box at pos.   9378 ggcgataat Score    49
-35 box at pos.   9362 ttgtgg    Score    24
Promoter Pos:   4770 LDF-  0.77
-10 box at pos.   4755 ggtcattct Score    40
-35 box at pos.   4737 tcgcta    Score    16
Promoter Pos:   4335 LDF-  0.70
-10 box at pos.   4320 gggaacaat Score    39
-35 box at pos.   4298 ttaccg    Score    32
Promoter Pos:  10909 LDF-  0.69
-10 box at pos.  10894 cgctatcgt Score    42
-35 box at pos.  10873 ctgctg    Score    12
Promoter Pos:   1187 LDF-  0.65
-10 box at pos.   1172 ctttatgaa Score    28
-35 box at pos.   1152 atgaaa    Score    30
Promoter Pos:   2348 LDF-  0.65
-10 box at pos.   2333 cggcaaaat Score    57
-35 box at pos.   2308 tggtct    Score    11
Promoter Pos:  12290 LDF-  0.61
-10 box at pos.  12275 ggttatgct Score    64
-35 box at pos.  12251 ccgaaa    Score   -12
Promoter Pos:  10308 LDF-  0.57
-10 box at pos.  10293 ctctacgct Score    35
-35 box at pos.  10276 ttgatt    Score    53

Oligonucleotides from known TF binding sites:

For promoter at  12689:
       ihf:  CCGGCCTA at position   12632 Score -  35
       fis:  TGTAAATT at position   12648 Score -  15
      argR:  AATTCAAT at position   12652 Score -  12
       crp:  AATAAATT at position   12657 Score -   9
     rpoH3:  CTGATAAG at position   12682 Score -  17
      soxS:  GATAAGCG at position   12684 Score -   9
      metJ:  ATAAGCGT at position   12685 Score -  10
For promoter at    209:
      lexA:  TATATAAA at position     189 Score -  14
     argR2:  ATATAAAT at position     190 Score -  13
       fnr:  ATAAATAT at position     192 Score -   9
    rpoD18:  AAATATAT at position     194 Score -   7
       fis:  AAATGTGA at position     215 Score -  11
For promoter at   6278:
    rpoD15:  TTTTAACA at position    6243 Score -  10
       crp:  TTTGTGAG at position    6289 Score -  10
For promoter at   5654:
      nagC:  GAAATAAG at position    5630 Score -   8
```

```
No such sites for promoter at   9948
For promoter at   5163:
      tyrR:  TTTACAAT at position    5149 Score -   8
For promoter at   6854:
      nagC:  CATTTCAC at position    6854 Score -   6
No such sites for promoter at  11945
For promoter at   7366:
    rpoD17:  CAGCATAC at position    7363 Score -   6
No such sites for promoter at  11412
For promoter at    548:
       fis:  CTCATTTT at position     499 Score -   9
      fhlA:  TCATTTTC at position     500 Score -   7
       fur:  TAATGCTT at position     539 Score -   6
No such sites for promoter at   1961
For promoter at   1595:
      arcA:  AAAAGGGA at position    1562 Score -   8
For promoter at   8417:
    rpoD17:  CGCCTTTT at position    8369 Score -   7
No such sites for promoter at   7854
No such sites for promoter at   8769
For promoter at   3925:
     argR2:  CATATTTT at position    3913 Score -   8
No such sites for promoter at   9394
No such sites for promoter at   4770
No such sites for promoter at   4335
No such sites for promoter at  10909
For promoter at   1187:
      purR:  TGAGGAAA at position    1140 Score -   6
No such sites for promoter at   2348
No such sites for promoter at  12290
No such sites for promoter at  10308
```

V. natriegens *ATCC 14048* ccmA-I *chromosome1 bottom strand (2,315,980 .. 2,323,459)*

Length of sequence: 7480 bp

Threshold for promoters: 0.20

Number of predicted promoters: 20

```
Promoter Pos:   7433 LDF-  7.68
-10 box at pos.   7418 TTCTAAACT Score    64
-35 box at pos.   7397 TTGTTT    Score    40
Promoter Pos:   1230 LDF-  4.39
-10 box at pos.   1215 GGTTTTACT Score    42
-35 box at pos.   1195 TTGATA    Score    58
Promoter Pos:     61 LDF-  4.19
-10 box at pos.     46 CGGTAGGTT Score    37
-35 box at pos.     27 TTTAAT    Score    36
Promoter Pos:   4044 LDF-  4.10
-10 box at pos.   4029 GTTTATTCT Score    59
-35 box at pos.   4004 TTGCTT    Score    48
Promoter Pos:   6009 LDF-  3.67
-10 box at pos.   5994 TCGCAAAAT Score    47
-35 box at pos.   5972 TTGCTT    Score    48
Promoter Pos:    894 LDF-  3.43
-10 box at pos.    879 TACTATTAT Score    60
-35 box at pos.    857 TTAATA    Score    35
Promoter Pos:   3273 LDF-  3.21
-10 box at pos.   3257 TTTTATGTT Score    50
-35 box at pos.   3238 TTGTTG    Score    39
```

```
Promoter Pos:   4885 LDF-  3.14
-10 box at pos.   4870 CTTTACATT Score    46
-35 box at pos.   4847 TTGATC    Score    36
Promoter Pos:   1565 LDF-  3.14
-10 box at pos.   1550 TACTACGAT Score    42
-35 box at pos.   1533 TTAACC    Score    21
Promoter Pos:    422 LDF-  2.76
-10 box at pos.    407 CGGTATTAT Score    76
-35 box at pos.    382 TTACAG    Score    26
Promoter Pos:   5603 LDF-  2.49
-10 box at pos.   5588 CTTTACAAT Score    66
-35 box at pos.   5571 CTGATA    Score    23
Promoter Pos:   2098 LDF-  2.39
-10 box at pos.   2083 CTTTACCAT Score    48
-35 box at pos.   2061 TTTATA    Score    39
Promoter Pos:   7126 LDF-  1.75
-10 box at pos.   7111 CGTTAGCAT Score    54
-35 box at pos.   7086 ATGCCA    Score    31
Promoter Pos:   4382 LDF-  1.49
-10 box at pos.   4367 TGGTGATCT Score    25
-35 box at pos.   4345 TTGTCA    Score    53
Promoter Pos:   2908 LDF-  1.42
-10 box at pos.   2893 AGGTATTCT Score    65
-35 box at pos.   2876 GTGACC    Score    11
Promoter Pos:   2580 LDF-  1.35
-10 box at pos.   2565 CGTCAAGCT Score    30
-35 box at pos.   2547 GTGAAA    Score    27
Promoter Pos:   6315 LDF-  1.33
-10 box at pos.   6300 ATTTCGGAT Score    10
-35 box at pos.   6280 TTGAAA    Score    60
Promoter Pos:   5207 LDF-  1.10
-10 box at pos.   5191 GGGTAAACC Score    36
-35 box at pos.   5175 TTGAGT    Score    38
Promoter Pos:   3596 LDF-  0.87
-10 box at pos.   3581 TTCTACAAG Score    27
-35 box at pos.   3557 TTGATG    Score    52
Promoter Pos:   6720 LDF-  0.82
-10 box at pos.   6704 GGATATTAT Score    65
-35 box at pos.   6688 TTGAGC    Score    21

Oligonucleotides from known TF binding sites:

For promoter at   7433:
    rpoD16:  TTTTTATA at position    7400 Score -  13
       fis:  TATACTTA at position    7404 Score -  18
    rpoD17:  ATACTTAA at position    7405 Score -  13
    rpoD17:  TTTTATAG at position    7443 Score -  10
For promoter at   1230:
    rpoD15:  TCCTCTGT at position    1203 Score -   8
    rpoD17:  TTTTACTT at position    1217 Score -   8
For promoter at     61:
    rpoD16:  TTATAAAA at position       1 Score -   9
      ompR:  TTCTTTTT at position      20 Score -   8
      lexA:  TTTTTTTA at position      23 Score -  16
For promoter at   4044:
      soxS:  ATTTGTTA at position    4050 Score -  10
      dnaA:  TTGTTATC at position    4052 Score -   5
For promoter at   6009:
       ihf:  AAATAAAA at position    5999 Score -  13
      phoB:  AATAAAAG at position    6000 Score -   8
For promoter at    894:
       fis:  AAAAATAA at position     839 Score -   9
```

```
       ihf:  AAATAAAA at position     841 Score -  13
For promoter at   3273:
     flhCD:  GGGCTTTT at position    3267 Score -   9
For promoter at   4885:
      metJ:  CTTTACAT at position    4870 Score -  14
      ompR:  TTTACATT at position    4871 Score -  11
For promoter at   1565:
      arcA:  TTAACTAA at position    1523 Score -  12
    rpoD17:  CCAAATAG at position    1537 Score -   8
     rpoH2:  AAATAATG at position    1576 Score -   5
       fur:  ATAATGAT at position    1578 Score -   6
No such sites for promoter at    422
For promoter at   5603:
      tyrR:  TTTACAAT at position    5589 Score -   8
For promoter at   2098:
    rpoD16:  TTTTTATA at position    2059 Score -  13
No such sites for promoter at   7126
For promoter at   4382:
      purR:  GAAACGAG at position    4332 Score -   6
      phoB:  TGTCATCA at position    4346 Score -   5
No such sites for promoter at   2908
For promoter at   2580:
    rpoD17:  AAAAATAG at position    2555 Score -   9
No such sites for promoter at   6315
For promoter at   5207:
    rpoD17:  CAAGAGGG at position    5150 Score -   7
No such sites for promoter at   3596
No such sites for promoter at   6720
```

V. natriegens *ATCC 14048* napFDAB *chromosome 2 bottom strand (1,611,395 ... 1,616,456)*

Length of sequence: 5062 bp

Threshold for promoters: 0.20

Number of predicted promoters: 13

```
Promoter Pos:   2292 LDF-  4.33
-10 box at pos.   2277 cggtaaaat Score    83
-35 box at pos.   2255 tttaca    Score    47
Promoter Pos:    220 LDF-  3.86
-10 box at pos.    205 ggctaagct Score    52
-35 box at pos.    186 tttatt    Score    34
Promoter Pos:   4961 LDF-  3.61
-10 box at pos.   4946 tgggataat Score    58
-35 box at pos.   4923 atgcat    Score    20
Promoter Pos:   4561 LDF-  3.37
-10 box at pos.   4546 ctttagaat Score    65
-35 box at pos.   4526 tcgtat    Score     5
Promoter Pos:   1104 LDF-  2.65
-10 box at pos.   1089 tatgaaaat Score    36
-35 box at pos.   1069 ttccca    Score    41
Promoter Pos:   1949 LDF-  2.63
-10 box at pos.   1934 cggtaaact Score    73
-35 box at pos.   1914 ctacat    Score    -8
Promoter Pos:   4158 LDF-  2.37
-10 box at pos.   4142 tatgaaaat Score    36
-35 box at pos.   4126 gtgcta    Score    20
Promoter Pos:   3285 LDF-  2.00
-10 box at pos.   3270 tggtaccat Score    60
-35 box at pos.   3251 ttcttg    Score    19
```

```
Promoter Pos:   3675 LDF-  1.47
-10 box at pos.   3660 tgaaaaaat Score    39
-35 box at pos.   3639 ttaaag    Score    31
Promoter Pos:   2599 LDF-  1.36
-10 box at pos.   2584 ggttaccgt Score    32
-35 box at pos.   2564 ttaaca    Score    43
Promoter Pos:   1539 LDF-  1.01
-10 box at pos.   1524 cggtatgtt Score    54
-35 box at pos.   1499 tagaga    Score     6
Promoter Pos:   2937 LDF-  0.74
-10 box at pos.   2922 acgtgaact Score    20
-35 box at pos.   2905 ttccct    Score    36
Promoter Pos:    610 LDF-  0.59
-10 box at pos.    594 cggtgatat Score    31
-35 box at pos.    578 ttgaat    Score    55

Oligonucleotides from known TF binding sites:

No such sites for promoter at   2292
For promoter at    220:
      phoB:  TTTATTAA at position     186 Score -   8
      metR:  CAAATTTT at position     225 Score -   8
For promoter at   4961:
    rpoD15:  TTTTGTTT at position    4965 Score -   9
    rpoD17:  TTTGTTTT at position    4966 Score -   9
For promoter at   4561:
    rpoD16:  TAGAATGC at position    4549 Score -  13
For promoter at   1104:
    rpoD17:  CCAAATTG at position    1072 Score -   6
    rpoD18:  AATTGAGG at position    1075 Score -  11
No such sites for promoter at   1949
For promoter at   4158:
    rpoD17:  ATACTTAA at position    4149 Score -  13
    rpoD19:  TACTTAAA at position    4150 Score -   8
    rpoD17:  TTGGAATA at position    4163 Score -   8
No such sites for promoter at   3285
No such sites for promoter at   3675
No such sites for promoter at   2599
No such sites for promoter at   1539
For promoter at   2937:
    rpoD17:  TTCCCTGT at position    2905 Score -   6
    rpoD17:  AACTAAAC at position    2927 Score -   7
No such sites for promoter at    610
```

V. natriegens *ATCC 14048* napGH *chromosome 2 bottom strand (975,950 .. 978,054)*

Length of sequence: 2105 bp

Threshold for promoters: 0.20

Number of predicted promoters: 6

```
Promoter Pos:    194 LDF-  5.48
-10 box at pos.    179 ttttatact Score    76
-35 box at pos.    162 ttgaaa    Score    60
Promoter Pos:   2085 LDF-  4.47
-10 box at pos.   2070 ggctatgat Score    69
-35 box at pos.   2049 ttgcga    Score    38
Promoter Pos:   1626 LDF-  2.68
-10 box at pos.   1611 aggtacgat Score    57
-35 box at pos.   1588 ttgatg    Score    52
```

```
Promoter Pos:    937 LDF-  2.66
-10 box at pos.    922 cgctaagct Score    51
-35 box at pos.    897 ttcaca    Score    46
Promoter Pos:   1323 LDF-  1.19
-10 box at pos.   1308 gggtaatct Score    60
-35 box at pos.   1285 gggcca    Score    -9
Promoter Pos:    629 LDF-  0.54
-10 box at pos.    614 ccatacgat Score    32
-35 box at pos.    589 ttgtgg    Score    24

Oligonucleotides from known TF binding sites:

For promoter at    194:
    rpoD16:  TTTTTATA at position     178 Score -  13
For promoter at   2085:
      soxS:  ATTTGTTA at position    2055 Score -  10
No such sites for promoter at   1626
For promoter at    937:
      dnaA:  ATTCACAA at position     896 Score -   5
For promoter at   1323:
      purR:  CGTTTTTT at position    1299 Score -   8
No such sites for promoter at    629
```

V. natriegens *ATCC 14048* napC *chromosome 2 top strand (1,303,543 .. 1,304,928)*

Length of sequence: 1386 bp

Threshold for promoters: 0.20

Number of predicted promoters: 3

```
Promoter Pos:     98 LDF-  7.43
-10 box at pos.     83 atttatttt Score    47
-35 box at pos.     66 ttgcat    Score    50
Promoter Pos:    575 LDF-  2.36
-10 box at pos.    560 aggcaaaat Score    56
-35 box at pos.    542 ttacca    Score    38
Promoter Pos:    971 LDF-  1.62
-10 box at pos.    959 ggttaaact Score    73
-35 box at pos.    931 ttgtca    Score    53

Oligonucleotides from known TF binding sites:

For promoter at     98:
      argR:  TTTTTTAT at position      43 Score -  13
    rpoD16:  TTTTTATA at position      44 Score -  13
       crp:  GTTCAATT at position      56 Score -   6
    rpoD16:  TTATAAAA at position      72 Score -   9
     argR2:  TTTATTTT at position      84 Score -   7
    rpoD17:  ATTTTGTA at position      87 Score -   9
    rpoD15:  TTTTGTAA at position      88 Score -  11
No such sites for promoter at    575
No such sites for promoter at    971
```

Supplementary Tables

Supplementary Table 1: Strains, plasmids, and primers

Name	Description	Reference
**Plasmids**		
pUC57	Cloning vector with ampicillin resistance marker	GeneCust (Boynes, France)
pET16bP	Modified pET16b vector with extended multiple cloning site and ampicillin resistance marker; Utilizes the T7 expression system	U. Wehemeyer (unpublished); sequence available in Supplementary Data 1
pEC86	Expression vector containing the *ccmA-H* genes from *E. coli* K-12 under the control of a *tet* promoter	Culture Collection of Switzerland (CCOS891)
pUC57_cycA	pUC57 vector containing the optimized *cycA2* gene from *At. ferrooxidans* CCM4253 in the *Eco*RV/*Apa*I restriction site	This study; GeneCust (Boynes, France)
pUC57_cyc1	pUC57 vector containing the optimized *cyc1A* gene from *At. ferrooxidans* CCM4253 in the *Eco*RV/*Apa*I restriction site	This study; GeneCust (Boynes, France)
pUC57_rus	pUC57 vector containing the optimized *rus* gene from *At. ferrooxidans* CCM4253 in the *Eco*RV/*Apa*I restriction site	This study; GeneCust (Boynes, France)
pUC57_cyc2	pUC57 vector containing the optimized *cyc2A* gene from *At. ferrooxidans* CCM4253 in the *Eco*RV/*Apa*I restriction site	This study; GeneCust (Boynes, France)
pET16bP_cycA_cyc1_rus_cyc2	pET16bP vector containing the *cycA2*, *cyc1A*, *rus*, and *cyc2A* genes from *At. ferrooxidans* CCM4253 in the *Nco*I/*Not*I restriction site.	This study
**Primers**		
pET16-chk-rev	Reverse sequencing primer for the pET16bP vector<br>5'- CAGCTTCCTTTCGGGCTTTGTTAG -3'	U. Wehemeyer (unpublished)

Name	Description	Reference
T7_Promoter_mod	Forward sequencing primer for the pET16bP vector<br>5'- CTCACTATAGGGGAATTGTGAGCG -3'	This study; Eurofins
cycA_Seq_fw	Forward sequencing primer; binds near the 3' end of codon-optimized *cycA2*<br>5'- GTTGCTACCCACAAAGGTATC -3'	This study; Eurofins
cyc1_Seq_fw	Forward sequencing primer; binds near the 3' end of codon-optimized *cyc1A*<br>5'- GCTGACCTACTTCCACAACG -3'	This study; Eurofins
rus_Seq_fw	Forward sequencing primer; binds near the 3' end of codon-optimized *rus*<br>5'- GGTTACACCGACTTCACCTG -3'	This study; Eurofins
cyc2_Seq_fw	Forward sequencing primer; binds near the 3' end of codon-optimized *cyc2A*<br>5'- GACTGGAACTGGGAAGTTGG -3'	This study; Eurofins
**Strains**		
E. coli NEB5α	DH5α derivative; high efficiency cloning strain	New England BioLabs
E. coli T7 Express	BL21(DE3) derivative; Allows protein expression under a T7 promoter	New England BioLabs
V. natriegens VmaxX2	Derived from *V. natriegens* ATCC 14048; Allows protein expression under a T7 promoter	TelesisBio
T7 Express + pEC86 + pET16bP_cycA_cyc1_rus_cyc2	*E. coli* T7 Express carrying the pEC86 and pET16bP_cycA_cyc1_rus_cyc2 vectors	This study
T7 Express + pET16bP_cycA_cyc1_rus_cyc2	*E. coli* T7 Express carrying the pET16bP_cycA_cyc1_rus_cyc2 vector	This study
Vmax X2 + pEC86 + pET16bP_cycA_cyc1_rus_cyc2	*V. natriegens* Vmax X2 carrying the pEC86 and pET16bP_cycA_cyc1_rus_cyc2 vectors	This study
Vmax X2 + pET16bP_cycA_cyc1_rus_cyc2	*V. natriegens* Vmax X2 carrying the pET16bP_cycA_cyc1_rus_cyc2 vector	This study

Supplementary Table 2: Antibiotic concentrations used for selection

	NEB5α	T7 Express	Vmax X2	
			Solid media	Liquid media
**Ampicillin**	100 μg mL^{-1}	100 μg mL^{-1}	50 μg mL^{-1}	100 μg mL^{-1}
**Chloramphenicol**	33 μg mL^{-1}	33 μg mL^{-1}	12.5 μg mL^{-1}	25 μg mL^{-1}

Supplementary Table 3: Media recipes

	LB	LB + v2 salts	e2xYT	e2xYT + v2 salts	e2xYT based ZYM-5052	e2xYT based ZYM-5052 + v2 salts	SOC	SOC + v2 salts
**Tryptone / peptone**	5 g L^{-1}	5 g L^{-1}	20 g L^{-1}	20 g L^{-1}	20 g L^{-1}	20 g L^{-1}	20 g L^{-1}	20 g L^{-1}
**Yeast extract**	10 g L^{-1}	10 g L^{-1}	35 g L^{-1}	35 g L^{-1}	35 g L^{-1}	35 g L^{-1}	5 g L^{-1}	5 g L^{-1}
**NaCl**	10 g L^{-1}	10 g L^{-1}	5 g L^{-1}	5 g L^{-1}	-	-	0.5 g L^{-1}	0.5 g L^{-1}
**Agar-agar**	(15 g L^{-1})	(15 g L^{-1})	-	-	-	-	-	-
**20x v2 salts**	-	1x	-	1x	-	1x	-	1x
**25x M**	-	-	-	-	1x	1x	-	-
**50x 5052**	-	-	-	-	1x	1x	-	-
**500x trace metals**	-	-	-	-	0.2x	0.2x	-	-
**KCl**	-	-	-	-	-	-	2.5 mM	2.5 mM
**$MgSO_4 \cdot 7\ H_2O$**	-	-	-	-	-	-	10 mM	10 mM
**Glucose**	-	-	-	-	-	-	20 mM	20 mM
**pH**	7.0	7.0	7.0	7.0	7.0	7.0	7.0	7.4

LB and LB + v2 salts plates with appropriate antibiotics were used for selection after heat shock transformation and revitalization of cryo stocks. SOC and SOC + v2 salts media were used for recovery after heat shock transformation. Liquid LB and LB + v2 salts media were used for propagation of selected clones. Cryo stocks were kept in LB or LB + v2 salts medium containing 25% (v/v) glycerol at – 80 °C. All other media were used for protein production as described in the Methods section.

20x v2salts

Contained 4.08 M NaCl, 84 mM KCl, and 462.8 mM $MgCl_2 \cdot 6\ H_2O$ and was supplemented to all media used for *V. natriegens* Vmax X2 according to Weinstock et al. [7].

25x M

Contained 625 mM $Na_2HPO_4 \cdot 2\ H_2O$, 625 mM KH_2PO_4, 1.25 M NH_4Cl, and 125 mM Na_2SO_4 and was used for autoinduction media according to Studier [8].

50x 5052

Contained 25% (v/v) glycerol, 2.5% (w/v) glucose, and 10% (w/v) lactose and was used for autoinduction media according to Studier [8].

500x trace metals

Contained the following according to Studier [8]:

25 mM $FeCl_3$

10 mM $CaCl_2$

5 mM $MnCl_2$

5 mM $ZnSO_4$

1 mM $CoCl_2$

1 mM $CuCl_2$

1 mM $NiCl_2$

1 mM Na_2MoO_4

1 mM Na_2SeO_3 and

1 mM H_3BO_3 in ≈ 60 mM HCl

Supplementary Table 4: Biomass production in 50 mL cultures (cell wet weight)

Cells of a 50 mL culture were centrifuged at 5,000 xg for 5 min at 4 °C and the supernatant was discarded. The cell wet weight is given as the mean from quadruplicates with the standard deviation.

Strain	Cell wet weight [g]
Vmax X2 + pEC86 + pET16bP_cycA_cyc1_rus_cyc2	1.43 ± 0.19
Vmax X2 + pET16bP_cycA_cyc1_rus_cyc2	1.48 ± 0.04
T7 Express + pEC86 + pET16bP_cycA_cyc1_rus_cyc2	0.92 ± 0.03
T7 Express + pET16bP_cycA_cyc1_rus_cyc2	0.93 ± 0.08

Supplementary Table 5: BCA assay results

The total protein concentration of each fraction was determined in a 96-well plate with the Pierce BCA protein assay kit (Thermo Scientific) using triplicates according to the manufacturer's instructions. Calibration was performed according to the manufacturer's instructions with bovine serum albumin (BSA) in 1: diluted TES buffer in a 96-well plate using triplicates. TES buffer diluted 1:2 with water was used as blank for all measurements.

The following calibration equation was determined in the BSA concentration range of 0 – 750 μg mL^{-1}:

$$A_{562\,nm} = 6.59 \cdot 10^{-4} \cdot c_{protein}[\mu g\ mL^{-1}] \qquad R^2 = 9.862 \cdot 10^{-1}$$

Protein concentrations in the different fractions are given as the mean of triplicates with the standard deviation.

**Strain**	**Total protein concentration in fraction [mg mL^{-1}]**			
	**Periplasm (P)**	**Cytoplasm (C)**	**Cytoplasm + membranes (C+M)**	**Membranes (M)**
**Vmax X2 + pEC86 + pET16bP_cycA_cyc1_rus_cyc2**	12.5 ± 0.2	22.0 ± 0.3	19.1 ± 0.3	13.1 ± 2.0
**Vmax X2 + pET16bP_cycA_cyc1_rus_cyc2**	10.9 ± 0.3	22.4 ± 0.7	19.8 ± 0.7	16.4 ± 0.8
**T7 Express + pEC86 + pET16bP_cycA_cyc1_rus_cyc2**	7.2 ± 0.5	12.8 ± 0.6	20.5 ± 0.2	9.0 ± 0.8
**T7 Express + pET16bP_cycA_cyc1_rus_cyc2**	8.7 ± 0.9	21.0 ± 2.3	22.7 ± 1.0	9.5 ± 0.5

Supplementary Data

Supplementary Data 1: Plasmid sequences

Sequences correspond to the top strand in 5' to 3' direction.

pUC57_cycA sequence

TCGCGCGTTTCGGTGATGACGGTGAAAACCTCTGACACATGCAGCTCCCGGAGACGGTCACAGCTTGTCTGTAAG
CGGATGCCGGGAGCAGACAAGCCCGTCAGGGCGCGTCAGCGGGTGTTGGCGGGTGTCGGGGCTGGCTTAACTATG
CGGCATCAGAGCAGATTGTACTGAGAGTGCACCATATGCGGTGTGAAATACCGCACAGATGCGTAAGGAGAAAAT
ACCGCATCAGGCGCCATTCGCCATTCAGGCTGCGCAACTGTTGGGAAGGGCGATCGGTGCGGGCCTCTTCGCTAT
TACGCCAGCTGGCGAAAGGGGGATGTGCTGCAAGGCGATTAAGTTGGGTAACGCCAGGGTTTTCCCAGTCACGAC
GTTGTAAAACGACGGCCAGTGAATTCGAGCTCGGTACCTCGCGAATGCATCTAGATATCACTGCATATGTAACCA
TGGTATAAGGAGATATATATGCCGGCTCTGACCACCAAAGTTCCGCACATGCTGCAGAAAAAACCGCGTCTGTCT
GTTATCCCGCTGTTCTCTATGGCTATGGTTATGGCTTCTGCTGCTTACGGTTCTCCGCTGGCTCAGACCGTTTCT
GCTGCTCCGGCTGCTGCTACCGCTTCTGCTGTTTCTGCTACCGCTGCTACCGCTTCTGTTACCGCTTCTGGTATC
CCGACCGTTGTTCAGTCTACCTGCATGGCTTGCCACGGTATGCAGGGTATCGCTGCTGACGGTGGTATGTTCCCG
AACCTGGCTGGTCAGTGGAAACCGTACCTGCTGCGTCAGCTGGACCACTTCAAAACCCACGTTCGTGCTGACCCG
CAGTCTCCGATCATGTGGGGTATGGCTGCTCCGCTGACCGCTGCTCAGATGCAGCAGGTTGCTGACTACTTCTCT
TCTCAGAAACCGGCTTCTGGTCACGTTTACGACCCGAAACTGGTTGCTGAAGGTAAAAAACTGTACTTCGGTGGT
CTGCCGGACAAACACATGCCGGCTTGCATGGCTTGCCACGGTGCTACCCTGGCTGGTCTGCCGCCGTACTTCCCG
CGTCTGGCTGGTCAGAAACGTACCTACGTTATCAACCAGCTGACCTACTTCAAATCTGGTCAGCGTGTTGCTACC
CACAAAGGTATCATGCAGTACGTTGCTTCTCGTCTGAACCCGAAACAGATCACCGCTCTGGCTGCTTACATCCGT
TCTCGTTAAACTAGTACTGCGGCCGCACTTTAATTAATGGGGCCCGTCGACTGCAGAGGCCTGCATGCAAGCTTG
GCGTAATCATGGTCATAGCTGTTTCCTGTGTGAAATTGTTATCCGCTCACAATTCCACACAACATACGAGCCGGA
AGCATAAAGTGTAAAGCCTGGGGTGCCTAATGAGTGAGCTAACTCACATTAATTGCGTTGCGCTCACTGCCCGCT
TTCCAGTCGGGAAACCTGTCGTGCCAGCTGCATTAATGAATCGGCCAACGCGCGGGGAGAGGCGGTTTGCGTATT
GGGCGCTCTTCCGCTTCCTCGCTCACTGACTCGCTGCGCTCGGTCGTTCGGCTGCGGCGAGCGGTATCAGCTCAC
TCAAAGGCGGTAATACGGTTATCCACAGAATCAGGGGATAACGCAGGAAAGAACATGTGAGCAAAAGGCCAGCAA
AAGGCCAGGAACCGTAAAAAGGCCGCGTTGCTGGCGTTTTTCCATAGGCTCCGCCCCCCTGACGAGCATCACAAA
AATCGACGCTCAAGTCAGAGGTGGCGAAACCCGACAGGACTATAAAGATACCAGGCGTTTCCCCCTGGAAGCTCC
CTCGTGCGCTCTCCTGTTCCGACCCTGCCGCTTACCGGATACCTGTCCGCCTTTCTCCCTTCGGGAAGCGTGGCG
CTTTCTCATAGCTCACGCTGTAGGTATCTCAGTTCGGTGTAGGTCGTTCGCTCCAAGCTGGGCTGTGTGCACGAA
CCCCCCGTTCAGCCCGACCGCTGCGCCTTATCCGGTAACTATCGTCTTGAGTCCAACCCGGTAAGACACGACTTA
TCGCCACTGGCAGCAGCCACTGGTAACAGGATTAGCAGAGCGAGGTATGTAGGCGGTGCTACAGAGTTCTTGAAG
TGGTGGCCTAACTACGGCTACACTAGAAGAACAGTATTTGGTATCTGCGCTCTGCTGAAGCCAGTTACCTTCGGA
AAAAGAGTTGGTAGCTCTTGATCCGGCAAACAAACCACCGCTGGTAGCGGTGGTTTTTTTGTTTGCAAGCAGCAG
ATTACGCGCAGAAAAAAAGGATCTCAAGAAGATCCTTTGATCTTTTCTACGGGGTCTGACGCTCAGTGGAACGAA
AACTCACGTTAAGGGATTTTGGTCATGAGATTATCAAAAAGGATCTTCACCTAGATCCTTTTAAATTAAAAATGA
AGTTTTAAATCAATCTAAAGTATATATGAGTAAACTTGGTCTGACAGTTACCAATGCTTAATCAGTGAGGCACCT
ATCTCAGCGATCTGTCTATTTCGTTCATCCATAGTTGCCTGACTCCCCGTCGTGTAGATAACTACGATACGGGAG
GGCTTACCATCTGGCCCCAGTGCTGCAATGATACCGCGAGACCCACGCTCACCGGCTCCAGATTTATCAGCAATA
AACCAGCCAGCCGGAAGGGCCGAGCGCAGAAGTGGTCCTGCAACTTTATCCGCCTCCATCCAGTCTATTAATTGT

TGCCGGGAAGCTAGAGTAAGTAGTTCGCCAGTTAATAGTTTGCGCAACGTTGTTGCCATTGCTACAGGCATCGTG
GTGTCACGCTCGTCGTTTGGTATGGCTTCATTCAGCTCCGGTTCCCAACGATCAAGGCGAGTTACATGATCCCCC
ATGTTGTGCAAAAAAGCGGTTAGCTCCTTCGGTCCTCCGATCGTTGTCAGAAGTAAGTTGGCCGCAGTGTTATCA
CTCATGGTTATGGCAGCACTGCATAATTCTCTTACTGTCATGCCATCCGTAAGATGCTTTTCTGTGACTGGTGAG
TACTCAACCAAGTCATTCTGAGAATAGTGTATGCGGCGACCGAGTTGCTCTTGCCCGGCGTCAATACGGGATAAT
ACCGCGCCACATAGCAGAACTTTAAAAGTGCTCATCATTGGAAAACGTTCTTCGGGGCGAAAACTCTCAAGGATC
TTACCGCTGTTGAGATCCAGTTCGATGTAACCCACTCGTGCACCCAACTGATCTTCAGCATCTTTTACTTTCACC
AGCGTTTCTGGGTGAGCAAAAACAGGAAGGCAAAATGCCGCAAAAAAGGGAATAAGGGCGACACGGAAATGTTGA
ATACTCATACTCTTCCTTTTTCAATATTATTGAAGCATTTATCAGGGTTATTGTCTCATGAGCGGATACATATTT
GAATGTATTTAGAAAAATAAACAAATAGGGGTTCCGCGCACATTTCCCCGAAAAGTGCCACCTGACGTCTAAGAA
ACCATTATTATCATGACATTAACCTATAAAAATAGGCGTATCACGAGGCCCTTTCGTC

pUC57_cyc1 sequence

TCGCGCGTTTCGGTGATGACGGTGAAAACCTCTGACACATGCAGCTCCCGGAGACGGTCACAGCTTGTCTGTAAG
CGGATGCCGGGAGCAGACAAGCCCGTCAGGGCGCGTCAGCGGGTGTTGGCGGGTGTCGGGGCTGGCTTAACTATG
CGGCATCAGAGCAGATTGTACTGAGAGTGCACCATATGCGGTGTGAAATACCGCACAGATGCGTAAGGAGAAAAT
ACCGCATCAGGCGCCATTCGCCATTCAGGCTGCGCAACTGTTGGGAAGGGCGATCGGTGCGGGCCTCTTCGCTAT
TACGCCAGCTGGCGAAAGGGGGATGTGCTGCAAGGCGATTAAGTTGGGTAACGCCAGGGTTTTCCCAGTCACGAC
GTTGTAAAACGACGGCCAGTGAATTCGAGCTCGGTACCTCGCGAATGCATCTAGATATCACTGCATATGACTCCA
TGGTAAACTAGTACTAAGGAGATATATATGACCACCTACCTGTCTCAGGACCGTCTGCGTAACAAAGAAAACGAC
ACCATGACCCACCCGCACTCTAAAATGTACAAATCTAAAACCTTCCTGCTGTTCTCTGCTCTGCTGCTGGTTGCT
GGTCAGGCTTCTGCTGCTGTTGGTTCTGCTGACGCTCCGGCTCCGTACCGTGTTTCTTCTGACTGCATGGTTTGC
CACGGTATGACCGGTCGTGACACCCTGTACCCGATCGTTCCGCGTCTGGCTGGTCAGCACAAATCTTACCTGGAA
GCTCAGCTGAAAGCTTACAAAGACCACTCTCGTGCTGACCAGAACGGTGAAATCTACATGTGGCCGGTTGCTCAG
GCTCTGGACTCTGCTAAAATCACCGCTCTGGCTGACTACTTCAACGCTCAGAAACCGCCGATGCAGTCTTCTGGT
ATCAAACACGCTGGTGTTAAAGAAGGTAAAGCTATCTTCAACCAGGGTGTTACCAACGAACAGATCCCGGCTTGC
ATGGAATGCCACGGTTCTGCTGGTCAGGGTGCTGGTCCGTTCCCGCGTCTGGCTGGTCAGCGTTACGGTTACATC
ATCCAGCAGCTGACCTACTTCCACAACGGTACTCGTGTTAACACCCTGATGAACCAGATCGCTAAAAACATCACC
GTTGCTCAGATGAAAGACGTTGCTGCTTACCTGTCTTCTCTGTAAGAGCTCACTGGTACCACGCGGCCGCACTGT
TAATTAATGGGGCCCGTCGACTGCAGAGGCCTGCATGCAAGCTTGGCGTAATCATGGTCATAGCTGTTTCCTGTG
TGAAATTGTTATCCGCTCACAATTCCACACAACATACGAGCCGGAAGCATAAAGTGTAAAGCCTGGGGTGCCTAA
TGAGTGAGCTAACTCACATTAATTGCGTTGCGCTCACTGCCCGCTTTCCAGTCGGGAAACCTGTCGTGCCAGCTG
CATTAATGAATCGGCCAACGCGCGGGGAGAGGCGGTTTGCGTATTGGGCGCTCTTCCGCTTCCTCGCTCACTGAC
TCGCTGCGCTCGGTCGTTCGGCTGCGGCGAGCGGTATCAGCTCACTCAAAGGCGGTAATACGGTTATCCACAGAA
TCAGGGGATAACGCAGGAAAGAACATGTGAGCAAAAGGCCAGCAAAAGGCCAGGAACCGTAAAAAGGCCGCGTTG
CTGGCGTTTTTCCATAGGCTCCGCCCCCCTGACGAGCATCACAAAAATCGACGCTCAAGTCAGAGGTGGCGAAAC
CCGACAGGACTATAAAGATACCAGGCGTTTCCCCCTGGAAGCTCCCTCGTGCGCTCTCCTGTTCCGACCCTGCCG
CTTACCGGATACCTGTCCGCCTTTCTCCCTTCGGGAAGCGTGGCGCTTTCTCATAGCTCACGCTGTAGGTATCTC
AGTTCGGTGTAGGTCGTTCGCTCCAAGCTGGGCTGTGTGCACGAACCCCCCGTTCAGCCCGACCGCTGCGCCTTA
TCCGGTAACTATCGTCTTGAGTCCAACCCGGTAAGACACGACTTATCGCCACTGGCAGCAGCCACTGGTAACAGG
ATTAGCAGAGCGAGGTATGTAGGCGGTGCTACAGAGTTCTTGAAGTGGTGGCCTAACTACGGCTACACTAGAAGA
ACAGTATTTGGTATCTGCGCTCTGCTGAAGCCAGTTACCTTCGGAAAAAGAGTTGGTAGCTCTTGATCCGGCAAA

CAAACCACCGCTGGTAGCGGTGGTTTTTTTGTTTGCAAGCAGCAGATTACGCGCAGAAAAAAAGGATCTCAAGAA
GATCCTTTGATCTTTTCTACGGGGTCTGACGCTCAGTGGAACGAAAACTCACGTTAAGGGATTTTGGTCATGAGA
TTATCAAAAAGGATCTTCACCTAGATCCTTTTAAATTAAAAATGAAGTTTTAAATCAATCTAAAGTATATATGAG
TAAACTTGGTCTGACAGTTACCAATGCTTAATCAGTGAGGCACCTATCTCAGCGATCTGTCTATTTCGTTCATCC
ATAGTTGCCTGACTCCCCGTCGTGTAGATAACTACGATACGGGAGGGCTTACCATCTGGCCCCAGTGCTGCAATG
ATACCGCGAGACCCACGCTCACCGGCTCCAGATTTATCAGCAATAAACCAGCCAGCCGGAAGGGCCGAGCGCAGA
AGTGGTCCTGCAACTTTATCCGCCTCCATCCAGTCTATTAATTGTTGCCGGGAAGCTAGAGTAAGTAGTTCGCCA
GTTAATAGTTTGCGCAACGTTGTTGCCATTGCTACAGGCATCGTGGTGTCACGCTCGTCGTTTGGTATGGCTTCA
TTCAGCTCCGGTTCCCAACGATCAAGGCGAGTTACATGATCCCCCATGTTGTGCAAAAAAGCGGTTAGCTCCTTC
GGTCCTCCGATCGTTGTCAGAAGTAAGTTGGCCGCAGTGTTATCACTCATGGTTATGGCAGCACTGCATAATTCT
CTTACTGTCATGCCATCCGTAAGATGCTTTTCTGTGACTGGTGAGTACTCAACCAAGTCATTCTGAGAATAGTGT
ATGCGGCGACCGAGTTGCTCTTGCCCGGCGTCAATACGGGATAATACCGCGCCACATAGCAGAACTTTAAAAGTG
CTCATCATTGGAAAACGTTCTTCGGGGCGAAAACTCTCAAGGATCTTACCGCTGTTGAGATCCAGTTCGATGTAA
CCCACTCGTGCACCCAACTGATCTTCAGCATCTTTTACTTTCACCAGCGTTTCTGGGTGAGCAAAAACAGGAAGG
CAAAATGCCGCAAAAAAGGGAATAAGGGCGACACGGAAATGTTGAATACTCATACTCTTCCTTTTTCAATATTAT
TGAAGCATTTATCAGGGTTATTGTCTCATGAGCGGATACATATTTGAATGTATTTAGAAAAATAAACAAATAGGG
GTTCCGCGCACATTTCCCCGAAAAGTGCCACCTGACGTCTAAGAAACCATTATTATCATGACATTAACCTATAAA
AATAGGCGTATCACGAGGCCCTTTCGTC

pUC57_rus sequence

TCGCGCGTTTCGGTGATGACGGTGAAAACCTCTGACACATGCAGCTCCCGGAGACGGTCACAGCTTGTCTGTAAG
CGGATGCCGGGAGCAGACAAGCCCGTCAGGGCGCGTCAGCGGGTGTTGGCGGGTGTCGGGGCTGGCTTAACTATG
CGGCATCAGAGCAGATTGTACTGAGAGTGCACCATATGCGGTGTGAAATACCGCACAGATGCGTAAGGAGAAAAT
ACCGCATCAGGCGCCATTCGCCATTCAGGCTGCGCAACTGTTGGGAAGGGCGATCGGTGCGGGCCTCTTCGCTAT
TACGCCAGCTGGCGAAAGGGGGATGTGCTGCAAGGCGATTAAGTTGGGTAACGCCAGGGTTTTCCCAGTCACGAC
GTTGTAAAACGACGGCCAGTGAATTCGAGCTCGGTACCTCGCGAATGCATCTAGATATCACTGCATATGACTCCA
TGGACTACTAGTACTGAGCTCTGAAGGAGATATATAATGTACACCCAGAACACCATGAAAAAAAACTGGTACGTT
ACCGTTGGTGCTGCTGCTGCTCTGGCTGCTACCGTTGGTATGGGTACTGCTATGGCTGGTACTCTGGACACCACC
TGGAAAGAAGCTACCCTGCCGCAGGTTAAAGCTATGCTGGAAAAAGACACCGGTAAAGTTTCTGGTGACACCGTT
ACCTACTCTGGTAAAACCGTTCACGTTGTTGCTGCTGCTGTTCTGCCGGGTTTCCCGTTCCCGTCTTTCGAAGTT
CACGACAAAAAAAACCCGACCCTGGAAATCCCGGCTGGTGCTACCGTTGACGTTACCTTCATCAACACCAACAAA
GGTTTCGGTCACTCTTTCGACATCACCAAAAAAGGTCCGCCGTACGCTGTTATGCCGGTTATCGACCCGATCGTT
GCTGGTACTGGTTTCTCTCCGGTTCCGAAAGACGGTAAATTCGGTTACACCGACTTCACCTGGCACCCGACCGCT
GGTACTTACTACTACGTTTGCCAGATCCCGGGTCACGCTGCTACCGGTATGTTCGGTAAAATCGTTGTTAAATAA
GGTACCACGCGGCCGCACTGTTAATTAATGGGGCCCGTCGACTGCAGAGGCCTGCATGCAAGCTTGGCGTAATCA
TGGTCATAGCTGTTTCCTGTGTGAAATTGTTATCCGCTCACAATTCCACACAACATACGAGCCGGAAGCATAAAG
TGTAAAGCCTGGGGTGCCTAATGAGTGAGCTAACTCACATTAATTGCGTTGCGCTCACTGCCCGCTTTCCAGTCG
GGAAACCTGTCGTGCCAGCTGCATTAATGAATCGGCCAACGCGCGGGGAGAGGCGGTTTGCGTATTGGGCGCTCT
TCCGCTTCCTCGCTCACTGACTCGCTGCGCTCGGTCGTTCGGCTGCGGCGAGCGGTATCAGCTCACTCAAAGGCG
GTAATACGGTTATCCACAGAATCAGGGGATAACGCAGGAAAGAACATGTGAGCAAAAGGCCAGCAAAAGGCCAGG
AACCGTAAAAAGGCCGCGTTGCTGGCGTTTTTCCATAGGCTCCGCCCCCCTGACGAGCATCACAAAAATCGACGC
TCAAGTCAGAGGTGGCGAAACCCGACAGGACTATAAAGATACCAGGCGTTTCCCCCTGGAAGCTCCCTCGTGCGC

TCTCCTGTTCCGACCCTGCCGCTTACCGGATACCTGTCCGCCTTTCTCCCTTCGGGAAGCGTGGCGCTTTCTCAT
AGCTCACGCTGTAGGTATCTCAGTTCGGTGTAGGTCGTTCGCTCCAAGCTGGGCTGTGTGCACGAACCCCCCGTT
CAGCCCGACCGCTGCGCCTTATCCGGTAACTATCGTCTTGAGTCCAACCCGGTAAGACACGACTTATCGCCACTG
GCAGCAGCCACTGGTAACAGGATTAGCAGAGCGAGGTATGTAGGCGGTGCTACAGAGTTCTTGAAGTGGTGGCCT
AACTACGGCTACACTAGAAGAACAGTATTTGGTATCTGCGCTCTGCTGAAGCCAGTTACCTTCGGAAAAAGAGTT
GGTAGCTCTTGATCCGGCAAACAAACCACCGCTGGTAGCGGTGGTTTTTTTGTTTGCAAGCAGCAGATTACGCGC
AGAAAAAAAGGATCTCAAGAAGATCCTTTGATCTTTTCTACGGGGTCTGACGCTCAGTGGAACGAAAACTCACGT
TAAGGGATTTTGGTCATGAGATTATCAAAAAGGATCTTCACCTAGATCCTTTTAAATTAAAAATGAAGTTTTAAA
TCAATCTAAAGTATATATGAGTAAACTTGGTCTGACAGTTACCAATGCTTAATCAGTGAGGCACCTATCTCAGCG
ATCTGTCTATTTCGTTCATCCATAGTTGCCTGACTCCCCGTCGTGTAGATAACTACGATACGGGAGGGCTTACCA
TCTGGCCCCAGTGCTGCAATGATACCGCGAGACCCACGCTCACCGGCTCCAGATTTATCAGCAATAAACCAGCCA
GCCGGAAGGGCCGAGCGCAGAAGTGGTCCTGCAACTTTATCCGCCTCCATCCAGTCTATTAATTGTTGCCGGGAA
GCTAGAGTAAGTAGTTCGCCAGTTAATAGTTTGCGCAACGTTGTTGCCATTGCTACAGGCATCGTGGTGTCACGC
TCGTCGTTTGGTATGGCTTCATTCAGCTCCGGTTCCCAACGATCAAGGCGAGTTACATGATCCCCCATGTTGTGC
AAAAAAGCGGTTAGCTCCTTCGGTCCTCCGATCGTTGTCAGAAGTAAGTTGGCCGCAGTGTTATCACTCATGGTT
ATGGCAGCACTGCATAATTCTCTTACTGTCATGCCATCCGTAAGATGCTTTTCTGTGACTGGTGAGTACTCAACC
AAGTCATTCTGAGAATAGTGTATGCGGCGACCGAGTTGCTCTTGCCCGGCGTCAATACGGGATAATACCGCGCCA
CATAGCAGAACTTTAAAAGTGCTCATCATTGGAAAACGTTCTTCGGGGCGAAAACTCTCAAGGATCTTACCGCTG
TTGAGATCCAGTTCGATGTAACCCACTCGTGCACCCAACTGATCTTCAGCATCTTTTACTTTCACCAGCGTTTCT
GGGTGAGCAAAAACAGGAAGGCAAAATGCCGCAAAAAAGGGAATAAGGGCGACACGGAAATGTTGAATACTCATA
CTCTTCCTTTTTCAATATTATTGAAGCATTTATCAGGGTTATTGTCTCATGAGCGGATACATATTTGAATGTATT
TAGAAAAATAAACAAATAGGGGTTCCGCGCACATTTCCCCGAAAAGTGCCACCTGACGTCTAAGAAACCATTATT
ATCATGACATTAACCTATAAAAATAGGCGTATCACGAGGCCCTTTCGTC

pUC57_cyc2 sequence

TCGCGCGTTTCGGTGATGACGGTGAAAACCTCTGACACATGCAGCTCCCGGAGACGGTCACAGCTTGTCTGTAAG
CGGATGCCGGGAGCAGACAAGCCCGTCAGGGCGCGTCAGCGGGTGTTGGCGGGTGTCGGGGCTGGCTTAACTATG
CGGCATCAGAGCAGATTGTACTGAGAGTGCACCATATGCGGTGTGAAATACCGCACAGATGCGTAAGGAGAAAAT
ACCGCATCAGGCGCCATTCGCCATTCAGGCTGCGCAACTGTTGGGAAGGGCGATCGGTGCGGGCCTCTTCGCTAT
TACGCCAGCTGGCGAAAGGGGGATGTGCTGCAAGGCGATTAAGTTGGGTAACGCCAGGGTTTTCCCAGTCACGAC
GTTGTAAAACGACGGCCAGTGAATTCGAGCTCGGTACCTCGCGAATGCATCTAGATATCACTGCATATGACTCCA
TGGACTGGTACCACAAGGAGATATAAAATGGTTTCTTCTTCTGTTGGTTTCAAAAAAAAACGTCTGATCGTTGCT
CTGGCTGCTGTTGGTGGTATGGCTCTGTCTTCTGGTGCTTGGGCTCTGCCGTCTTTCGCTCGTCAGACCGGTTGG
TCTTGCGCTGCTTGCCACACCTCTTACCCGCAGCTGACCCCGATGGGTCGTATGTTCAAACTGCTGGGTTTCACC
ACCACCAACCTGCAGCGTCAGCAGAAACTGCAGGCTAAATTCGGTAACTCTGTTGGTCTGCTGATCTCTCGTGTT
TCTCAGTTCTCTATCTTCCTGCAGGCTTCTGCTACCAACGTTGGTGGTGGTCAGGCTGTTTTCGGTCCGGGTAAC
TCTAACGCTGGTGCTTCTCCGAACAACAACGTTCAGTTCCCGCAGCAGGTTTCTCTGTTCTACGCTGGTGAAATC
ACCCCGCACATCGGTTCTTTCCTGCACCTGACCTACTCTGGTGGTGGTTCTGGTGCTGGTGCTGGTGGTTTCTCT
TTCGACGACTCTTCTATCGTTTGGACCCACCCGTGGAAACTGGGTACTAACAACCTGCTGGTTACCGGTGTTGAC
GTTAACAACACCCCGACCGCTATGGACCTGTGGAACACCACCCCGGACTGGCAGGCTCCGTTCTTCTCTTCTGAC
TACTCTTCTTGGGGTCACGTTCCGCAGCCGTTCATCGAATCTTCTGCTGGTGCTGGTTACCCGCTGGCTGGTGTT
GGTGTTTACGGTGCTGACATCTTCGGTCCGAACCGTGCTAACTGGCTGTACGCTGACGCTGACGTTTACACCAAC

GGTCAGGGTACTCAGGTTAACCCGGTTGGTGGTTTCACCGCTGCTGGTCCGCAGGGTCGTCTGTCTGGTGGTGCT

CCGTACGTTCGTCTGGCTTACCAGCACGACTGGGGTGACTGGAACTGGGAAGTTGGTACTTTCGGTATGTGGTCT

TCTGTTTACGACAACACCATCAACAACACCCTGAACAAAGCTGGTGGTCCGATCGACACCTTCGACGACTACGAC

CTGGACACCCAGCTGCAGTGGCTGGACACCAACGACAACAACAACGTTACCATCCGTGCTGCTTGGGTTAACGAA

CAGCAGCAGTTCGGTGCTGGTAACGTTATCTCTTCTAACTCTTCTGGTAACCTGAACTTCTTCAACATCAACGCT

ACCTACTGGTATCACGACCACTACGGTATCCAGGGTGGTTACCGTAACGTTTGGGGTTCTGCTAACCCGGGTCTG

TACGGTACTACCTACACCAACTCTGGTTCTCCGGACACCTCTAACGAATGGATCGAAGCTTCTTACCTGCCGTGG

TGGAACACCCGTTTCTCTCTGCGTTACGTTGTTTACAACAAATTCAACGGTGTTGGTTCTGCTTCTTCTAACAAC

CTGGGTTACGGTGCTTCTGCTTACAACACCCTGGAACTGCTGGCTTGGATCTCTTACTAAGCGGCCGCACTGTTA

ATTAATGGGGCCCGTCGACTGCAGAGGCCTGCATGCAAGCTTGGCGTAATCATGGTCATAGCTGTTTCCTGTGTG

AAATTGTTATCCGCTCACAATTCCACACAACATACGAGCCGGAAGCATAAAGTGTAAAGCCTGGGGTGCCTAATG

AGTGAGCTAACTCACATTAATTGCGTTGCGCTCACTGCCCGCTTTCCAGTCGGGAAACCTGTCGTGCCAGCTGCA

TTAATGAATCGGCCAACGCGCGGGGAGAGGCGGTTTGCGTATTGGGCGCTCTTCCGCTTCCTCGCTCACTGACTC

GCTGCGCTCGGTCGTTCGGCTGCGGCGAGCGGTATCAGCTCACTCAAAGGCGGTAATACGGTTATCCACAGAATC

AGGGGATAACGCAGGAAAGAACATGTGAGCAAAAGGCCAGCAAAAGGCCAGGAACCGTAAAAAGGCCGCGTTGCT

GGCGTTTTTCCATAGGCTCCGCCCCCCTGACGAGCATCACAAAAATCGACGCTCAAGTCAGAGGTGGCGAAACCC

GACAGGACTATAAAGATACCAGGCGTTTCCCCCTGGAAGCTCCCTCGTGCGCTCTCCTGTTCCGACCCTGCCGCT

TACCGGATACCTGTCCGCCTTTCTCCCTTCGGGAAGCGTGGCGCTTTCTCATAGCTCACGCTGTAGGTATCTCAG

TTCGGTGTAGGTCGTTCGCTCCAAGCTGGGCTGTGTGCACGAACCCCCCGTTCAGCCCGACCGCTGCGCCTTATC

CGGTAACTATCGTCTTGAGTCCAACCCGGTAAGACACGACTTATCGCCACTGGCAGCAGCCACTGGTAACAGGAT

TAGCAGAGCGAGGTATGTAGGCGGTGCTACAGAGTTCTTGAAGTGGTGGCCTAACTACGGCTACACTAGAAGAAC

AGTATTTGGTATCTGCGCTCTGCTGAAGCCAGTTACCTTCGGAAAAAGAGTTGGTAGCTCTTGATCCGGCAAACA

AACCACCGCTGGTAGCGGTGGTTTTTTTGTTTGCAAGCAGCAGATTACGCGCAGAAAAAAAGGATCTCAAGAAGA

TCCTTTGATCTTTTCTACGGGGTCTGACGCTCAGTGGAACGAAAACTCACGTTAAGGGATTTTGGTCATGAGATT

ATCAAAAAGGATCTTCACCTAGATCCTTTTAAATTAAAAATGAAGTTTTAAATCAATCTAAAGTATATATGAGTA

AACTTGGTCTGACAGTTACCAATGCTTAATCAGTGAGGCACCTATCTCAGCGATCTGTCTATTTCGTTCATCCAT

AGTTGCCTGACTCCCCGTCGTGTAGATAACTACGATACGGGAGGGCTTACCATCTGGCCCCAGTGCTGCAATGAT

ACCGCGAGACCCACGCTCACCGGCTCCAGATTTATCAGCAATAAACCAGCCAGCCGGAAGGGCCGAGCGCAGAAG

TGGTCCTGCAACTTTATCCGCCTCCATCCAGTCTATTAATTGTTGCCGGGAAGCTAGAGTAAGTAGTTCGCCAGT

TAATAGTTTGCGCAACGTTGTTGCCATTGCTACAGGCATCGTGGTGTCACGCTCGTCGTTTGGTATGGCTTCATT

CAGCTCCGGTTCCCAACGATCAAGGCGAGTTACATGATCCCCCATGTTGTGCAAAAAAGCGGTTAGCTCCTTCGG

TCCTCCGATCGTTGTCAGAAGTAAGTTGGCCGCAGTGTTATCACTCATGGTTATGGCAGCACTGCATAATTCTCT

TACTGTCATGCCATCCGTAAGATGCTTTTCTGTGACTGGTGAGTACTCAACCAAGTCATTCTGAGAATAGTGTAT

GCGGCGACCGAGTTGCTCTTGCCCGGCGTCAATACGGGATAATACCGCGCCACATAGCAGAACTTTAAAAGTGCT

CATCATTGGAAAACGTTCTTCGGGGCGAAAACTCTCAAGGATCTTACCGCTGTTGAGATCCAGTTCGATGTAACC

CACTCGTGCACCCAACTGATCTTCAGCATCTTTTACTTTCACCAGCGTTTCTGGGTGAGCAAAAACAGGAAGGCA

AAATGCCGCAAAAAAGGGAATAAGGGCGACACGGAAATGTTGAATACTCATACTCTTCCTTTTTCAATATTATTG

AAGCATTTATCAGGGTTATTGTCTCATGAGCGGATACATATTTGAATGTATTTAGAAAAATAAACAAATAGGGGT

TCCGCGCACATTTCCCCGAAAAGTGCCACCTGACGTCTAAGAAACCATTATTATCATGACATTAACCTATAAAAA

TAGGCGTATCACGAGGCCCTTTCGTC

pET16bP sequence

Lower case letters indicate the altered multiple cloning site.

```
TAATGCGGTAGTTTATCACAGTTAAATTGCTAACGCAGTCAGGCACCGTGTATGAAATCTAACAATGCGCTCATC
GTCATCCTCGGCACCGTCACCCTGGATGCTGTAGGCATAGGCTTGGTTATGCCGGTACTGCCGGGCCTCTTGCGG
GATATCCGGATATAGTTCCTCCTTTCAGCAAAAAACCCCTCAAGACCCGTTTAGAGGCCCCAAGGGGTTATGCTA
GTTATTGCTCAGCGGTGGCAGCAGCCAACTCAGCTTCCTTTCGGGCTTTGTTAGCAGCCggatccacgcgtaact
taagtgcggccgcggtaccgggcccgtcgactctagagctctgcaggcatgcgatatccatatgACGACCTTCGA
TATGGCCGCTGCTGTGATGATGATGATGATGATGATGATGATGGCCCATGGTATATCTCCTTCTTAAAGTTAAAC
AAAATTATTTCTAGAGGGGAATTGTTATCCGCTCACAATTCCCCTATAGTGAGTCGTATTAATTTCGCGGATCG
AGATCTCGATCCTCTACGCCGGACGCATCGTGGCCGGCATCACCGGCGCCACAGGTGCGGTTGCTGGCGCCTATA
TCGCCGACATCACCGATGGGGAAGATCGGGCTCGCCACTTCGGGCTCATGAGCGCTTGTTTCGGCGTGGGTATGG
TGGCAGGCCCCGTGGCCGGGGGACTGTTGGGCGCCATCTCCTTGCATGCACCATTCCTTGCGGCGGCGGTGCTCA
ACGGCCTCAACCTACTACTGGGCTGCTTCCTAATGCAGGAGTCGCATAAGGGAGAGCGTCGAGATCCCGGACACC
ATCGAATGGCGCAAAACCTTTCGCGGTATGGCATGATAGCGCCCGGAAGAGAGTCAATTCAGGGTGGTGAATGTG
AAACCAGTAACGTTATACGATGTCGCAGAGTATGCCGGTGTCTCTTATCAGACCGTTTCCCGCGTGGTGAACCAG
GCCAGCCACGTTTCTGCGAAAACGCGGGAAAAAGTGGAAGCGGCGATGGCGGAGCTGAATTACATTCCCAACCGC
GTGGCACAACAACTGGCGGGCAAACAGTCGTTGCTGATTGGCGTTGCCACCTCCAGTCTGGCCCTGCACGCGCCG
TCGCAAATTGTCGCGGCGATTAAATCTCGCGCCGATCAACTGGGTGCCAGCGTGGTGGTGTCGATGGTAGAACGA
AGCGGCGTCGAAGCCTGTAAAGCGGCGGTGCACAATCTTCTCGCGCAACGCGTCAGTGGGCTGATCATTAACTAT
CCGCTGGATGACCAGGATGCCATTGCTGTGGAAGCTGCCTGCACTAATGTTCCGGCGTTATTTCTTGATGTCTCT
GACCAGACACCCATCAACAGTATTATTTTCTCCCATGAAGACGGTACGCGACTGGGCGTGGAGCATCTGGTCGCA
TTGGGTCACCAGCAAATCGCGCTGTTAGCGGGCCCATTAAGTTCTGTCTCGGCGCGTCTGCGTCTGGCTGGCTGG
CATAAATATCTCACTCGCAATCAAATTCAGCCGATAGCGGAACGGGAAGGCGACTGGAGTGCCATGTCCGGTTTT
CAACAAACCATGCAAATGCTGAATGAGGGCATCGTTCCCACTGCGATGCTGGTTGCCAACGATCAGATGGCGCTG
GGCGCAATGCGCGCCATTACCGAGTCCGGGCTGCGCGTTGGTGCGGATATCTCGGTAGTGGGATACGACGATACC
GAAGACAGCTCATGTTATATCCCGCCGTTAACCACCATCAAACAGGATTTTCGCCTGCTGGGGCAAACCAGCGTG
GACCGCTTGCTGCAACTCTCTCAGGGCCAGGCGGTGAAGGGCAATCAGCTGTTGCCCGTCTCACTGGTGAAAAGA
AAAACCACCCTGGCGCCCAATACGCAAACCGCCTCTCCCCGCGCGTTGGCCGATTCATTAATGCAGCTGGCACGA
CAGGTTTCCCGACTGGAAAGCGGGCAGTGAGCGCAACGCAATTAATGTAAGTTAGCTCACTCATTAGGCACCGGG
ATCTCGACCGATGCCCTTGAGAGCCTTCAACCCAGTCAGCTCCTTCCGGTGGGCGCGGGGCATGACTATCGTCGC
CGCACTTATGACTGTCTTCTTTATCATGCAACTCGTAGGACAGGTGCCGGCAGCGCTCTGGGTCATTTTCGGCGA
GGACCGCTTTCGCTGGAGCGCGACGATGATCGGCCTGTCGCTTGCGGTATTCGGAATCTTGCACGCCCTCGCTCA
AGCCTTCGTCACTGGTCCCGCCACCAAACGTTTCGGCGAGAAGCAGGCCATTATCGCCGGCATGGCGGCCGACGC
GCTGGGCTACGTCTTGCTGGCGTTCGCGACGCGAGGCTGGATGGCCTTCCCCATTATGATTCTTCTCGCTTCCGG
CGGCATCGGGATGCCCGCGTTGCAGGCCATGCTGTCCAGGCAGGTAGATGACGACCATCAGGGACAGCTTCAAGG
ATCGCTCGCGGCTCTTACCAGCCTAACTTCGATCACTGGACCGCTGATCGTCACGGCGATTTATGCCGCCTCGGC
GAGCACATGGAACGGGTTGGCATGGATTGTAGGCGCCGCCCTATACCTTGTCTGCCTCCCCGCGTTGCGTCGCGG
TGCATGGAGCCGGGCCACCTCGACCTGAATGGAAGCCGGCGGCACCTCGCTAACGGATTCACCACTCCAAGAATT
GGAGCCAATCAATTCTTGCGGAGAACTGTGAATGCGCAAACCAACCCTTGGCAGAACATATCCATCGCGTCCGCC
ATCTCCAGCAGCCGCACGCGGCGCATCTCGGGCAGCGTTGGGTCCTGGCCACGGGTGCGCATGATCGTGCTCCTG
TCGTTGAGGACCCGGCTAGGCTGGCGGGGTTGCCTTACTGGTTAGCAGAATGAATCACCGATACGCGAGCGAACG
```

TGAAGCGACTGCTGCTGCAAAACGTCTGCGACCTGAGCAACAACATGAATGGTCTTCGGTTTCCGTGTTTCGTAA
AGTCTGGAAACGCGGAAGTCAGCGCCCTGCACCATTATGTTCCGGATCTGCATCGCAGGATGCTGCTGGCTACCC
TGTGGAACACCTACATCTGTATTAACGAAGCGCTGGCATTGACCCTGAGTGATTTTTCTCTGGTCCCGCCGCATC
CATACCGCCAGTTGTTTACCCTCACAACGTTCCAGTAACCGGGCATGTTCATCATCAGTAACCCGTATCGTGAGC
ATCCTCTCTCGTTTCATCGGTATCATTACCCCCATGAACAGAAATCCCCCTTACACGGAGGCATCAGTGACCAAA
CAGGAAAAAACCGCCCTTAACATGGCCCGCTTTATCAGAAGCCAGACATTAACGCTTCTGGAGAAACTCAACGAG
CTGGACGCGGATGAACAGGCAGACATCTGTGAATCGCTTCACGACCACGCTGATGAGCTTTACCGCAGCTGCCTC
GCGCGTTTCGGTGATGACGGTGAAAACCTCTGACACATGCAGCTCCCGGAGACGGTCACAGCTTGTCTGTAAGCG
GATGCCGGGAGCAGACAAGCCCGTCAGGGCGCGTCAGCGGGTGTTGGCGGGTGTCGGGGCGCAGCCATGACCCAG
TCACGTAGCGATAGCGGAGTGTATACTGGCTTAACTATGCGGCATCAGAGCAGATTGTACTGAGAGTGCACCATA
TATGCGGTGTGAAATACCGCACAGATGCGTAAGGAGAAAATACCGCATCAGGCGCTCTTCCGCTTCCTCGCTCAC
TGACTCGCTGCGCTCGGTCGTTCGGCTGCGGCGAGCGGTATCAGCTCACTCAAAGGCGGTAATACGGTTATCCAC
AGAATCAGGGGATAACGCAGGAAAGAACATGTGAGCAAAAGGCCAGCAAAAGGCCAGGAACCGTAAAAAGGCCGC
GTTGCTGGCGTTTTTCCATAGGCTCCGCCCCCCTGACGAGCATCACAAAAATCGACGCTCAAGTCAGAGGTGGCG
AAACCCGACAGGACTATAAAGATACCAGGCGTTTCCCCCTGGAAGCTCCCTCGTGCGCTCTCCTGTTCCGACCCT
GCCGCTTACCGGATACCTGTCCGCCTTTCTCCCTTCGGGAAGCGTGGCGCTTTCTCATAGCTCACGCTGTAGGTA
TCTCAGTTCGGTGTAGGTCGTTCGCTCCAAGCTGGGCTGTGTGCACGAACCCCCCGTTCAGCCCGACCGCTGCGC
CTTATCCGGTAACTATCGTCTTGAGTCCAACCCGGTAAGACACGACTTATCGCCACTGGCAGCAGCCACTGGTAA
CAGGATTAGCAGAGCGAGGTATGTAGGCGGTGCTACAGAGTTCTTGAAGTGGTGGCCTAACTACGGCTACACTAG
AAGGACAGTATTTGGTATCTGCGCTCTGCTGAAGCCAGTTACCTTCGGAAAAAGAGTTGGTAGCTCTTGATCCGG
CAAACAAACCACCGCTGGTAGCGGTGGTTTTTTTGTTTGCAAGCAGCAGATTACGCGCAGAAAAAAAGGATCTCA
AGAAGATCCTTTGATCTTTTCTACGGGGTCTGACGCTCAGTGGAACGAAAACTCACGTTAAGGGATTTTGGTCAT
GAGATTATCAAAAAGGATCTTCACCTAGATCCTTTTAAATTAAAAATGAAGTTTTAAATCAATCTAAAGTATATA
TGAGTAAACTTGGTCTGACAGTTACCAATGCTTAATCAGTGAGGCACCTATCTCAGCGATCTGTCTATTTCGTTC
ATCCATAGTTGCCTGACTCCCCGTCGTGTAGATAACTACGATACGGGAGGGCTTACCATCTGGCCCCAGTGCTGC
AATGATACCGCGAGACCCACGCTCACCGGCTCCAGATTTATCAGCAATAAACCAGCCAGCCGGAAGGGCCGAGCG
CAGAAGTGGTCCTGCAACTTTATCCGCCTCCATCCAGTCTATTAATTGTTGCCGGGAAGCTAGAGTAAGTAGTTC
GCCAGTTAATAGTTTGCGCAACGTTGTTGCCATTGCTGCAGGCATCGTGGTGTCACGCTCGTCGTTTGGTATGGC
TTCATTCAGCTCCGGTTCCCAACGATCAAGGCGAGTTACATGATCCCCCATGTTGTGCAAAAAAGCGGTTAGCTC
CTTCGGTCCTCCGATCGTTGTCAGAAGTAAGTTGGCCGCAGTGTTATCACTCATGGTTATGGCAGCACTGCATAA
TTCTCTTACTGTCATGCCATCCGTAAGATGCTTTTCTGTGACTGGTGAGTACTCAACCAAGTCATTCTGAGAATA
GTGTATGCGGCGACCGAGTTGCTCTTGCCCGGCGTCAACACGGGATAATACCGCGCCACATAGCAGAACTTTAAA
AGTGCTCATCATTGGAAAACGTTCTTCGGGGCGAAAACTCTCAAGGATCTTACCGCTGTTGAGATCCAGTTCGAT
GTAACCCACTCGTGCACCCAACTGATCTTCAGCATCTTTTACTTTCACCAGCGTTTCTGGGTGAGCAAAAACAGG
AAGGCAAAATGCCGCAAAAAAGGGAATAAGGGCGACACGGAAATGTTGAATACTCATACTCTTCCTTTTTCAATA
TTATTGAAGCATTTATCAGGGTTATTGTCTCATGAGCGGATACATATTTGAATGTATTTAGAAAAATAAACAAAT
AGGGGTTCCGCGCACATTTCCCCGAAAAGTGCCACCTGACGTCTAAGAAACCATTATTATCATGACATTAACCTA
TAAAAATAGGCGTATCACGAGGCCCTTTCGTCTTCAAGAA

pET16bP_cycA_cyc1_rus_cyc2 sequence

Lower case letters indicate the altered multiple cloning site.

TAATGCGGTAGTTTATCACAGTTAAATTGCTAACGCAGTCAGGCACCGTGTATGAAATCTAACAATGCGCTCATC
GTCATCCTCGGCACCGTCACCCTGGATGCTGTAGGCATAGGCTTGGTTATGCCGGTACTGCCGGGCCTCTTGCGG
GATATCCGGATATAGTTCCTCCTTTCAGCAAAAAACCCCTCAAGACCCGTTTAGAGGCCCCAAGGGGTTATGCTA
GTTATTGCTCAGCGGTGGCAGCAGCCAACTCAGCTTCCTTTCGGGCTTTGTTAGCAGCCggatccacgcgtaact
taagtgcGGCCGCTTAGTAAGAGATCCAAGCCAGCAGTTCCAGGGTGTTGTAAGCAGAAGCACCGTAACCCAGGT
TGTTAGAAGAAGCAGAACCAACACCGTTGAATTTGTTGTAAACAACGTAACGCAGAGAGAAACGGGTGTTCCACC
ACGGCAGGTAAGAAGCTTCGATCCATTCGTTAGAGGTGTCCGGAGAACCAGAGTTGGTGTAGGTAGTACCGTACA
GACCCGGGTTAGCAGAACCCCAAACGTTACGGTAACCACCCTGGATACCGTAGTGGTCGTGATACCAGTAGGTAG
CGTTGATGTTGAAGAAGTTCAGGTTACCAGAAGAGTTAGAAGAGATAACGTTACCAGCACCGAACTGCTGCTGTT
CGTTAACCCAAGCAGCACGGATGGTAACGTTGTTGTTGTCGTTGGTGTCCAGCCACTGCAGCTGGGTGTCCAGGT
CGTAGTCGTCGAAGGTGTCGATCGGACCACCAGCTTTGTTCAGGGTGTTGTTGATGGTGTTGTCGTAAACAGAAG
ACCACATACCGAAAGTACCAACTTCCCAGTTCCAGTCACCCCAGTCGTGCTGGTAAGCCAGACGAACGTACGGAG
CACCACCAGACAGACGACCCTGCGGACCAGCAGCGGTGAAACCACCAACCGGGTTAACCTGAGTACCCTGACCGT
TGGTGTAAACGTCAGCGTCAGCGTACAGCCAGTTAGCACGGTTCGGACCGAAGATGTCAGCACCGTAAACACCAA
CACCAGCCAGCGGGTAACCAGCACCAGCAGAAGATTCGATGAACGGCTGCGGAACGTGACCCCAAGAAGAGTAGT
CAGAAGAGAAGAACGGAGCCTGCCAGTCCGGGGTGGTGTTCCACAGGTCCATAGCGGTCGGGGTGTTGTTAACGT
CAACACCGGTAACCAGCAGGTTGTTAGTACCCAGTTTCCACGGGTGGGTCCAAACGATAGAAGAGTCGTCGAAAG
AGAAACCACCAGCACCAGCACCAGAACCACCACCAGAGTAGGTCAGGTGCAGGAAAGAACCGATGTGCGGGGTGA
TTTCACCAGCGTAGAACAGAGAAACCTGCTGCGGGAACTGAACGTTGTTGTTCGGAGAAGCACCAGCGTTAGAGT
TACCCGGACCGAAAACAGCCTGACCACCACCAACGTTGGTAGCAGAAGCCTGCAGGAAGATAGAGAACTGAGAAA
CACGAGAGATCAGCAGACCAACAGAGTTACCGAATTTAGCCTGCAGTTTCTGCTGACGCTGCAGGTTGGTGGTGG
TGAAACCCAGCAGTTTGAACATACGACCCATCGGGGTCAGCTGCGGGTAAGAGGTGTGGCAAGCAGCGCAAGACC
AACCGGTCTGACGAGCGAAAGACGGCAGAGCCCAAGCACCAGAAGACAGAGCCATACCACCAACAGCAGCCAGAG
CAACGATCAGACGTTTTTTTTTGAAACCAACAGAAGAAGAAACCATTTTATATCTCCTTGTGGTACCTTATTTAA
CAACGATTTTACCGAACATACCGGTAGCAGCGTGACCCGGGATCTGGCAAACGTAGTAGTAAGTACCAGCGGTCG
GGTGCCAGGTGAAGTCGGTGTAACCGAATTTACCGTCTTTCGGAACCGGAGAGAAACCAGTACCAGCAACGATCG
GGTCGATAACCGGCATAACAGCGTACGGCGGACCTTTTTTGGTGATGTCGAAAGAGTGACCGAAACCTTTGTTGG
TGTTGATGAAGGTAACGTCAACGGTAGCACCAGCCGGGATTTCCAGGGTCGGGTTTTTTTTGTCGTGAACTTCGA
AAGACGGGAACGGGAAACCCGGCAGAACAGCAGCAGCAACAACGTGAACGGTTTTACCAGAGTAGGTAACGGTGT
CACCAGAAACTTTACCGGTGTCTTTTTCCAGCATAGCTTTAACCTGCGGCAGGGTAGCTTCTTTCCAGGTGGTGT
CCAGAGTACCAGCCATAGCAGTACCCATACCAACGGTAGCAGCCAGAGCAGCAGCAGCACCAACGGTAACGTACC
AGTTTTTTTTCATGGTGTTCTGGGTGTACATTATATATCTCCTTCAGAGCTCTTACAGAGAAGACAGGTAAGCAG
CAACGTCTTTCATCTGAGCAACGGTGATGTTTTTAGCGATCTGGTTCATCAGGGTGTTAACACGAGTACCGTTGT
GGAAGTAGGTCAGCTGCTGGATGATGTAACCGTAACGCTGACCAGCCAGACGCGGGAACGGACCAGCACCCTGAC
CAGCAGAACCGTGGCATTCCATGCAAGCCGGGATCTGTTCGTTGGTAACACCCTGGTTGAAGATAGCTTTACCTT
CTTTAACACCAGCGTGTTTGATACCAGAAGACTGCATCGGCGGTTTCTGAGCGTTGAAGTAGTCAGCCAGAGCGG
TGATTTTAGCAGAGTCCAGAGCCTGAGCAACCGGCCACATGTAGATTTCACCGTTCTGGTCAGCACGAGAGTGGT
CTTTGTAAGCTTTCAGCTGAGCTTCCAGGTAAGATTTGTGCTGACCAGCCAGACGCGGAACGATCGGGTACAGGG
TGTCACGACCGGTCATACCGTGGCAAACCATGCAGTCAGAAGAAACACGGTACGGAGCCGGAGCGTCAGCAGAAC

CAACAGCAGCAGAAGCCTGACCAGCAACCAGCAGCAGAGCAGAGAACAGCAGGAAGGTTTTAGATTTGTACATTT
TAGAGTGCGGGTGGGTCATGGTGTCGTTTTCTTTGTTACGCAGACGGTCCTGAGACAGGTAGGTGGTCATATATA
TCTCCTTAGTACTAGTTTAACGAGAACGGATGTAAGCAGCCAGAGCGGTGATCTGTTTCGGGTTCAGACGAGAAG
CAACGTACTGCATGATACCTTTGTGGGTAGCAACACGCTGACCAGATTTGAAGTAGGTCAGCTGGTTGATAACGT
AGGTACGTTTCTGACCAGCCAGACGCGGGAAGTACGGCGGCAGACCAGCCAGGGTAGCACCGTGGCAAGCCATGC
AAGCCGGCATGTGTTTGTCCGGCAGACCACCGAAGTACAGTTTTTACCTTCAGCAACCAGTTTCGGGTCGTAAA
CGTGACCAGAAGCCGGTTTCTGAGAAGAGAAGTAGTCAGCAACCTGCTGCATCTGAGCAGCGGTCAGCGGAGCAG
CCATACCCCACATGATCGGAGACTGCGGGTCAGCACGAACGTGGGTTTTGAAGTGGTCCAGCTGACGCAGCAGGT
ACGGTTTCCACTGACCAGCCAGGTTCGGGAACATACCACCGTCAGCAGCGATACCCTGCATACCGTGGCAAGCCA
TGCAGGTAGACTGAACAACGGTCGGGATACCAGAAGCGGTAACAGAAGCGGTAGCAGCGGTAGCAGAAACAGCAG
AAGCGGTAGCAGCAGCCGGAGCAGCAGAAACGGTCTGAGCCAGCGGAGAACCGTAAGCAGCAGAAGCCATAACCA
TAGCCATAGAGAACAGCGGGATAACAGACAGACGCGGTTTTTCTGCAGCATGTGCGGAACTTTGGTGGTCAGAG
CCGGCATATATATCTCCTTATACCATGGTATATCTCCTTCTTAAAGTTAAACAAAATTATTTCTAGAGGGGAATT
GTTATCCGCTCACAATTCCCCTATAGTGAGTCGTATTAATTTCGCGGGATCGAGATCTCGATCCTCTACGCCGGA
CGCATCGTGGCCGGCATCACCGGCGCCACAGGTGCGGTTGCTGGCGCCTATATCGCCGACATCACCGATGGGGAA
GATCGGGCTCGCCACTTCGGGCTCATGAGCGCTTGTTTCGGCGTGGGTATGGTGGCAGGCCCCGTGGCCGGGGGA
CTGTTGGGCGCCATCTCCTTGCATGCACCATTCCTTGCGGCGGCGGTGCTCAACGGCCTCAACCTACTACTGGGC
TGCTTCCTAATGCAGGAGTCGCATAAGGGAGAGCGTCGAGATCCCGGACACCATCGAATGGCGCAAAACCTTTCG
CGGTATGGCATGATAGCGCCCGGAAGAGAGTCAATTCAGGGTGGTGAATGTGAAACCAGTAACGTTATACGATGT
CGCAGAGTATGCCGGTGTCTCTTATCAGACCGTTTCCCGCGTGGTGAACCAGGCCAGCCACGTTTCTGCGAAAAC
GCGGGAAAAAGTGGAAGCGGCGATGGCGGAGCTGAATTACATTCCCAACCGCGTGGCACAACAACTGGCGGGCAA
ACAGTCGTTGCTGATTGGCGTTGCCACCTCCAGTCTGGCCCTGCACGCGCCGTCGCAAATTGTCGCGGCGATTAA
ATCTCGCGCCGATCAACTGGGTGCCAGCGTGGTGGTGTCGATGGTAGAACGAAGCGGCGTCGAAGCCTGTAAAGC
GGCGGTGCACAATCTTCTCGCGCAACGCGTCAGTGGGCTGATCATTAACTATCCGCTGGATGACCAGGATGCCAT
TGCTGTGGAAGCTGCCTGCACTAATGTTCCGGCGTTATTTCTTGATGTCTCTGACCAGACACCCATCAACAGTAT
TATTTTCTCCCATGAAGACGGTACGCGACTGGGCGTGGAGCATCTGGTCGCATTGGGTCACCAGCAAATCGCGCT
GTTAGCGGGCCCATTAAGTTCTGTCTCGGCGCGTCTGCGTCTGGCTGGCTGGCATAAATATCTCACTCGCAATCA
AATTCAGCCGATAGCGGAACGGGAAGGCGACTGGAGTGCCATGTCCGGTTTTCAACAAACCATGCAAATGCTGAA
TGAGGGCATCGTTCCCACTGCGATGCTGGTTGCCAACGATCAGATGGCGCTGGGCGCAATGCGCGCCATTACCGA
GTCCGGGCTGCGCGTTGGTGCGGATATCTCGGTAGTGGGATACGACGATACCGAAGACAGCTCATGTTATATCCC
GCCGTTAACCACCATCAAACAGGATTTTCGCCTGCTGGGGCAAACCAGCGTGGACCGCTTGCTGCAACTCTCTCA
GGGCCAGGCGGTGAAGGGCAATCAGCTGTTGCCCGTCTCACTGGTGAAAAGAAAAACCACCCTGGCGCCCAATAC
GCAAACCGCCTCTCCCCGCGCGTTGGCCGATTCATTAATGCAGCTGGCACGACAGGTTTCCCGACTGGAAAGCGG
GCAGTGAGCGCAACGCAATTAATGTAAGTTAGCTCACTCATTAGGCACCGGGATCTCGACCGATGCCCTTGAGAG
CCTTCAACCCAGTCAGCTCCTTCCGGTGGGCGCGGGGCATGACTATCGTCGCCGCACTTATGACTGTCTTCTTTA
TCATGCAACTCGTAGGACAGGTGCCGGCAGCGCTCTGGGTCATTTTCGGCGAGGACCGCTTTCGCTGGAGCGCGA
CGATGATCGGCCTGTCGCTTGCGGTATTCGGAATCTTGCACGCCCTCGCTCAAGCCTTCGTCACTGGTCCCGCCA
CCAAACGTTTCGGCGAGAAGCAGGCCATTATCGCCGGCATGGCGGCCGACGCGCTGGGCTACGTCTTGCTGGCGT
TCGCGACGCGAGGCTGGATGGCCTTCCCCATTATGATTCTTCTCGCTTCCGGCGGCATCGGGATGCCCGCGTTGC
AGGCCATGCTGTCCAGGCAGGTAGATGACGACCATCAGGGACAGCTTCAAGGATCGCTCGCGGCTCTTACCAGCC
TAACTTCGATCACTGGACCGCTGATCGTCACGGCGATTTATGCCGCCTCGGCGAGCACATGGAACGGGTTGGCAT
GGATTGTAGGCGCCGCCCTATACCTTGTCTGCCTCCCCGCGTTGCGTCGCGGTGCATGGAGCCGGGCCACCTCGA

CCTGAATGGAAGCCGGCGGCACCTCGCTAACGGATTCACCACTCCAAGAATTGGAGCCAATCAATTCTTGCGGAG
AACTGTGAATGCGCAAACCAACCCTTGGCAGAACATATCCATCGCGTCCGCCATCTCCAGCAGCCGCACGCGGCG
CATCTCGGGCAGCGTTGGGTCCTGGCCACGGGTGCGCATGATCGTGCTCCTGTCGTTGAGGACCCGGCTAGGCTG
GCGGGGTTGCCTTACTGGTTAGCAGAATGAATCACCGATACGCGAGCGAACGTGAAGCGACTGCTGCTGCAAAAC
GTCTGCGACCTGAGCAACAACATGAATGGTCTTCGGTTTCCGTGTTTCGTAAAGTCTGGAAACGCGGAAGTCAGC
GCCCTGCACCATTATGTTCCGGATCTGCATCGCAGGATGCTGCTGGCTACCCTGTGGAACACCTACATCTGTATT
AACGAAGCGCTGGCATTGACCCTGAGTGATTTTTCTCTGGTCCCGCCGCATCCATACCGCCAGTTGTTTACCCTC
ACAACGTTCCAGTAACCGGGCATGTTCATCATCAGTAACCCGTATCGTGAGCATCCTCTCTCGTTTCATCGGTAT
CATTACCCCCATGAACAGAAATCCCCCTTACACGGAGGCATCAGTGACCAAACAGGAAAAAACCGCCCTTAACAT
GGCCCGCTTTATCAGAAGCCAGACATTAACGCTTCTGGAGAAACTCAACGAGCTGGACGCGGATGAACAGGCAGA
CATCTGTGAATCGCTTCACGACCACGCTGATGAGCTTTACCGCAGCTGCCTCGCGCGTTTCGGTGATGACGGTGA
AAACCTCTGACACATGCAGCTCCCGGAGACGGTCACAGCTTGTCTGTAAGCGGATGCCGGGAGCAGACAAGCCCG
TCAGGGCGCGTCAGCGGGTGTTGGCGGGTGTCGGGGCGCAGCCATGACCCAGTCACGTAGCGATAGCGGAGTGTA
TACTGGCTTAACTATGCGGCATCAGAGCAGATTGTACTGAGAGTGCACCATATATGCGGTGTGAAATACCGCACA
GATGCGTAAGGAGAAAATACCGCATCAGGCGCTCTTCCGCTTCCTCGCTCACTGACTCGCTGCGCTCGGTCGTTC
GGCTGCGGCGAGCGGTATCAGCTCACTCAAAGGCGGTAATACGGTTATCCACAGAATCAGGGGATAACGCAGGAA
AGAACATGTGAGCAAAAGGCCAGCAAAAGGCCAGGAACCGTAAAAAGGCCGCGTTGCTGGCGTTTTTCCATAGGC
TCCGCCCCCCTGACGAGCATCACAAAAATCGACGCTCAAGTCAGAGGTGGCGAAACCCGACAGGACTATAAAGAT
ACCAGGCGTTTCCCCCTGGAAGCTCCCTCGTGCGCTCTCCTGTTCCGACCCTGCCGCTTACCGGATACCTGTCCG
CCTTTCTCCCTTCGGGAAGCGTGGCGCTTTCTCATAGCTCACGCTGTAGGTATCTCAGTTCGGTGTAGGTCGTTC
GCTCCAAGCTGGGCTGTGTGCACGAACCCCCCGTTCAGCCCGACCGCTGCGCCTTATCCGGTAACTATCGTCTTG
AGTCCAACCCGGTAAGACACGACTTATCGCCACTGGCAGCAGCCACTGGTAACAGGATTAGCAGAGCGAGGTATG
TAGGCGGTGCTACAGAGTTCTTGAAGTGGTGGCCTAACTACGGCTACACTAGAAGGACAGTATTTGGTATCTGCG
CTCTGCTGAAGCCAGTTACCTTCGGAAAAAGAGTTGGTAGCTCTTGATCCGGCAAACAAACCACCGCTGGTAGCG
GTGGTTTTTTTGTTTGCAAGCAGCAGATTACGCGCAGAAAAAAAGGATCTCAAGAAGATCCTTTGATCTTTTCTA
CGGGGTCTGACGCTCAGTGGAACGAAAACTCACGTTAAGGGATTTTGGTCATGAGATTATCAAAAAGGATCTTCA
CCTAGATCCTTTTAAATTAAAAATGAAGTTTTAAATCAATCTAAAGTATATATGAGTAAACTTGGTCTGACAGTT
ACCAATGCTTAATCAGTGAGGCACCTATCTCAGCGATCTGTCTATTTCGTTCATCCATAGTTGCCTGACTCCCCG
TCGTGTAGATAACTACGATACGGGAGGGCTTACCATCTGGCCCCAGTGCTGCAATGATACCGCGAGACCCACGCT
CACCGGCTCCAGATTTATCAGCAATAAACCAGCCAGCCGGAAGGGCCGAGCGCAGAAGTGGTCCTGCAACTTTAT
CCGCCTCCATCCAGTCTATTAATTGTTGCCGGGAAGCTAGAGTAAGTAGTTCGCCAGTTAATAGTTTGCGCAACG
TTGTTGCCATTGCTGCAGGCATCGTGGTGTCACGCTCGTCGTTTGGTATGGCTTCATTCAGCTCCGGTTCCCAAC
GATCAAGGCGAGTTACATGATCCCCCATGTTGTGCAAAAAAGCGGTTAGCTCCTTCGGTCCTCCGATCGTTGTCA
GAAGTAAGTTGGCCGCAGTGTTATCACTCATGGTTATGGCAGCACTGCATAATTCTCTTACTGTCATGCCATCCG
TAAGATGCTTTTCTGTGACTGGTGAGTACTCAACCAAGTCATTCTGAGAATAGTGTATGCGGCGACCGAGTTGCT
CTTGCCCGGCGTCAACACGGGATAATACCGCGCCACATAGCAGAACTTTAAAAGTGCTCATCATTGGAAAACGTT
CTTCGGGGCGAAAACTCTCAAGGATCTTACCGCTGTTGAGATCCAGTTCGATGTAACCCACTCGTGCACCCAACT
GATCTTCAGCATCTTTTACTTTCACCAGCGTTTCTGGGTGAGCAAAAACAGGAAGGCAAAATGCCGCAAAAAAGG
GAATAAGGGCGACACGGAAATGTTGAATACTCATACTCTTCCTTTTTCAATATTATTGAAGCATTTATCAGGGTT
ATTGTCTCATGAGCGGATACATATTTGAATGTATTTAGAAAAATAAACAAATAGGGGTTCCGCGCACATTTCCCC
GAAAAGTGCCACCTGACGTCTAAGAAACCATTATTATCATGACATTAACCTATAAAAATAGGCGTATCACGAGGC
CCTTTCGTCTTCAAGAA

References

1. Thomas, P. E., Ryan, D. & Levin, W. An improved staining procedure for the detection of the peroxidase activity of cytochrome P-450 on sodium dodecyl sulfate polyacrylamide gels. *Analytical Biochemistry* **75,** 168–176 (1976).
2. Petiti, M., Houot, L. & Duché, D. Cell Fractionation. *Methods in molecular biology (Clifton, N.J.)* **1615,** 59–64 (2017).
3. Grote, A. *et al.* JCat: a novel tool to adapt codon usage of a target gene to its potential expression host. *Nucleic Acids Res* **33,** W526-31 (2005).
4. Teufel, F. *et al.* SignalP 6.0 predicts all five types of signal peptides using protein language models. *Nature biotechnology* **40,** 1023–1025 (2022).
5. Gasteiger, E. *et al.* Protein Identification and Analysis Tools on the ExPASy Server in *The Proteomics Protocols Handbook* (Humana Press, 2005), pp. 571–607.
6. Solovyev, V. & Salamov, A. Automatic Annotation of Microbial Genomes and Metagenomic Sequences in *Metagenomics and its Applications in Agriculture, Biomedicine and Environmental Studies* (2011), pp. 61–78.
7. Weinstock, M. T., Hesek, E. D., Wilson, C. M. & Gibson, D. G. *Vibrio natriegens* as a fast-growing host for molecular biology. *Nat Methods* **13,** 849–851 (2016).
8. Studier, F. W. Protein production by auto-induction in high density shaking cultures. *Protein Expression and Purification* **41,** 207–234 (2005).
